# Half a Century of Research on Posttraumatic Stress Disorder: A Scientometric Analysis

**DOI:** 10.2174/1570159X22666230927143106

**Published:** 2023-09-28

**Authors:** Michel Sabé, Chaomei Chen, Wissam El-Hage, Arnaud Leroy, Guillaume Vaiva, Silvia Monari, Natacha Premand, Javier Bartolomei, Stefano Caiolo, Andreas Maercker, Robert H. Pietrzak, Marylène Cloître, Stefan Kaiser, Marco Solmi

**Affiliations:** 1 Division of Adult Psychiatry, Department of Psychiatry, University Hospitals of Geneva, 2, Chemin du Petit-Bel-Air, CH-1226, Thonex, Switzerland;; 2 College of Computing & Informatics, Drexel University, Philadelphia, PA, USA;; 3 CHRU de Tours, Clinique Psychiatrique Universitaire, Centre Régional de Psychotraumatologie CVL, 37540 Saint-Cyr-sur-Loire, France; UMR 1253, iBrain, INSERM, Université de Tours, 37000 Tours, France;; 4 Univ Lille, INSERM, Lille Neuroscience & Cognition Centre (U-1172), Plasticity & SubjectivitY Team, CHU Lille, Fontan Hospital, General Psychiatry Department & Centre National de Ressources et Résilience Pour les Psychotraumatismes (CN2R Lille - Paris), 59000 Lille, France;; 5 CNRS UMR 9193-PsyCHIC-SCALab, & CHU Lille, Department of Psychiatry, Univ. Lille, F-59000, Lille, France;; 6 Department of Neuroscience (DNS), University of Padova, Padua, Italy;; 7 Division of Psychopathology and Clinical Intervention, University of Zurich, Zurich, Switzerland;; 8 Department of Psychiatry, Yale University School of Medicine, New Haven, CT, USA;; 9 US Department of Veterans Affairs National Center for Posttraumatic Stress Disorder, VA Connecticut Health Care System, West Haven, Connecticut, USA;; 10 National Center for PTSD Dissemination and Training Division, VA Palo Alto Health Care System, USA; and Department of Psychiatry and Behavioral Sciences, Stanford University, USA;; 11 Department of Psychiatry, University of Ottawa, Ontario, Canada;; 12 Department of Mental Health, The Ottawa Hospital, Ontario, Canada;; 13 Ottawa Hospital Research Institute (OHRI) Clinical Epidemiology Program University of Ottawa, Ontario, Ottawa;; 14 School of Epidemiology and Public Health, Faculty of Medicine, University of Ottawa, Ottawa, Canada;; 15 Department of Child and Adolescent Psychiatry, Charité Universit¨atsmedizin, Berlin, Germany

**Keywords:** Trauma, stress, evidence synthesis, neuroimaging, bibliometric, CiteSpace

## Abstract

We conducted a scientometric analysis to outline clinical research on posttraumatic stress disorder (PTSD). Our primary objective was to perform a broad-ranging scientometric analysis to evaluate key themes and trends over the past decades. Our secondary objective was to measure research network performance. We conducted a systematic search in the Web of Science Core Collection up to 15 August 2022 for publications on PTSD. We identified 42,170 publications published between 1945 and 2022. We used CiteSpace to retrieve the co-cited reference network (1978-2022) that presented significant modularity and mean silhouette scores, indicating highly credible clusters (Q = 0.915, S = 0.795). Four major trends of research were identified: ‘war veterans and refugees’, ‘treatment of PTSD/neuroimaging’, ‘evidence syntheses’, and ‘somatic symptoms of PTSD’. The largest cluster of research concerned evidence synthesis for genetic predisposition and environmental exposures leading to PTSD occurrence. Research on war-related trauma has shifted from battlefield-related in-person exposure trauma to drone operator trauma and is being out published by civilian-related trauma research, such as the ‘COVID-19’ pandemic impact, ‘postpartum’, and ‘grief disorder’. The focus on the most recent trends in the research revealed a burst in the ‘treatment of PTSD’ with the development of Mhealth, virtual reality, and psychedelic drugs. The collaboration networks reveal a central place for the USA research network, and although relatively isolated, a recent surge of publications from China was found. Compared to other psychiatric disorders, we found a lack of high-quality randomized controlled trials for pharmacological and nonpharmacological treatments. These results can inform funding agencies and future research.

## 1 INTRODUCTION

Posttraumatic stress disorder (PTSD) is a trauma or stress-related disorder developed by some individuals exposed to a stressful, frightening, or distressing event. PTSD presents a 5-10% lifetime prevalence in high-income countries [1], is twice as common in women as in men, and is associated with subsequent chronic physical conditions and morbidity [2]. Subjects with PTSD can present involuntarily re‐experiencing aspects of the traumatic event in the form of vivid intrusive memories, flashbacks, or trauma-related nightmares, with important functional impairment [3].

Ancient Greek and Mesopotamian texts already referred to PTSD as a dramatic consequence of battlefields, such as “ghost-induced mutism with vivid nightmares” [4]. Global attention to the impact of traumatic events on health has been brought by Holocaust survivor advocacy groups, as well as from research on Vietnam War veterans [5]. More recently, the advent of mechanized warfare conflicts has given birth to terms such as “soldier's heart”, “shell shock,” and “war neurosis” [6]. Nevertheless, modern epidemiology has revealed that the civilian population carries most of the burden of PTSD, with approximately half of the trauma experiences occurring during childhood (sexual assault, physical abuse, community violence, disasters) [7, 8], which are associated with significant alterations in functioning [9]. In 1980, the American Psychiatric Association (APA) added PTSD to the third edition of its Diagnostic and Statistical Manual of Mental Disorders (DSM-3) [10]. The current classification system DSM-5 defines PTSD based on symptoms of persistent re-experiencing of traumatic memories, avoidance of stimuli reminiscent of the traumatic event, negative alterations in cognition and mood, and alterations in arousal persisting for at least one month [11]. Over the last decades, the increased number of scientific publications has led modern medicine to describe altered brain circuitry in PTSD, such as a hyperactive amygdala response and a hyporesponse of the medial prefrontal cortex (mPFC) and hippocampus [12]. Despite thousands of systematic reviews and meta-analyses, such tremendous growth of publications requires new approaches to review and analyze trends within knowledge domains [13]. Scientometric analysis can be defined as a “quantitative study of science, communication in science, and science policy” [14]. This field based on bibliometrics, a quantitative method of citation and content analysis, is quickly evolving to synthesize evidence by informing on past and actual influence of research papers and their interconnections and by visualizing content across and within publications [15]. Witnessing the importance of these novel evidence synthesis methods, we identified a few bibliometric studies on PTSD focusing on specific topics, such as the use of mobile technologies and apps in combination with traditional treatment for PTSD [16] or the link between PTSD and the COVID-19 pandemic [17]. However, only one scientometric analysis was found, with a specific focus on military medicine through 2017 [18].

To the best of our knowledge, this is the first broad and comprehensive scientometric analysis conducted on PTSD, offering the opportunity to expand and ameliorate future research in the field, as well as inform country policies.

## 2 METHODS

### 2.1 Objectives

Our primary objective was to produce a comprehensive scientometric analysis of how to research trends on PTSD have evolved over decades by using a network of co-cited references and co-occurring keywords.

Our secondary objective was to provide clinicians and researchers with a measure of the collaborative network and a performance analysis (countries, institutions, authors, references, keywords, and journals). This study is based on a preregistered protocol, available on osf.io (link), and on a previously published scientometric analysis [19].

### 2.2 Search Strategy and Data Collection

We searched the Web of Science Core Collection (WOSCC) on July 1^st^, 2022, using a set of medical subject headings terms, and keywords: PTSD, psychological trauma, acute stress disorders, or traumatic stress disorder. The full search terms and strategy can be found on the preregistered protocol. Of importance, we excluded animal studies to focus on clinical research. WOSCC was selected, as it is one of the most informative databases in bibliometric analyses [20]. We limited the database source to the Science Citation Index Expanded, and we restrained publication types to articles, reviews, and proceedings papers, with no limitations on language, time, or populations. Irrelevant Web of Science categories were excluded (*e.g*., fisheries). The database that included full references and citations of retrieved articles was extracted in tag-delimited plain text files. All highly cited articles according to WOSCC were inspected. Duplicates were eliminated with CiteSpace. We detail all extraction processes and report all reasons for exclusion in our flowchart (Supplementary Fig. **S1**).

### 2.3 Data Analysis

Bibliometrix R packages (3.1.4) [21] were used to analyze information on publication growth, authors, and journals. Bibliometric outcomes included citation counts, co-citations, and co-occurrences. Citation counts are the number of direct citations to a publication. A co-citation is a specific form of document coupling, defined as the frequency with which two documents are cited together [22], and co-occurrence is a measure of the statistical correlation between the common co-occurrence of nodes (*e.g*., words) from a text. For each network, nodes can be different units of measure (*e.g*., authors, countries, institutions, journals, words). In co-cited reference networks, the nodes are highly co-cited papers, which extend from a single-slide equivalent to multiple-slice network analysis, *i.e*., a time series of networks to more effectively detect critical transitions over time. In this work, we retrieved the co-cited references, co-cited co-authors, co-cited institutions, and country networks as co-occurrence keyword networks using CiteSpace, a software designed for scientometric analysis [13].

CiteSpace uses a variety of metrics to generate network analyses, with structural (betweenness centrality, modularity, silhouette score) and temporal metrics (citation burstness). In graph theory, betweenness centrality is a measure of centrality in a graph, which is the extent to which a certain vertex lies on the shortest paths between other vertices. A high betweenness centrality score indicates a bridge-spanning role in a network [23]. The modularity score (Q) measures the strength of the division of a network into clusters, with the Q value ranging from 0 to +1. Values greater than 0.3 indicate a significant community structure. The silhouette score (S) evaluates the goodness of fit of a clustering technique, with S values ranging from -1 to +1. Values greater than 0.3, 0.5, or 0.7 indicate that the network is considered homogenous, reasonable, or highly credible, respectively. For both metrics, values close to 1 indicate an optimal clustering process. If possible, we will conduct a structural variation analysis to detect transformative papers, which are papers with the strongest modularity divergence scores, revealing novel connections.

The likelihood ratio test (*p* < 0.001) was used by CiteSpace to extract cluster labels from noun phrases of the keyword lists of co-cited articles in each cluster. Co-cited articles of each cluster were closely inspected to validate cluster labels, which were modified if needed.

For temporal metrics, burstiness is the intermittent increase and decrease in the activity or frequency of an event [24]. Finally, the sigma score combines both structural and temporal properties, namely, betweenness centrality and citation burst. To optimize the measure of citation performance, we used the g-index, an alternative to the h-index that gives more weight to highly cited articles [25].

All CiteSpace parameters used can be found in Supplementary Information 1.

## 3 RESULTS

### 3.1 Analysis of Publication Outputs, Major Journals, and Growth Trend Predictions

We retrieved 48,170 publications, which were reduced to 42,170 after data filtering, accumulating 1,0260,276 references from 35,333 articles and 6,837 reviews (Supplementary Fig. **S1**). These retrieved articles were available in 20 different languages, but 95.8% of the articles were in English.

‘War neuroses after psychological trauma’ published in 1945 by Gillespie RD was the first article identified [26]. For the 1948-2022 time period, 115,261 different authors were identified, the average number of co-authors per document was 5.51, and the average number of citations per document was 4 citations per year (Supplementary Fig. **S2**).

The number of publications was relatively stable from 1945 to 1980, with an average of 0.7 publications per year, and then exponentially increased, with an annual growth rate of 19.2% from 1980 to 2021; there were 3 publications in 1980, 45 in 1990, 418 in 2000 and 4,009 in 2021 (Supplementary Fig. **S3**). The top three journals, gathering the most important number of publications, were JAMA (21,805 articles), the American Journal of Psychiatry (17,713), and the Journal of Trauma and Stress (14,027) (Supplementary Table **S1**). The majority of articles were published in leading psychiatry and neuroscience journals (Supplementary Fig. **S4**).

### 3.2 Analysis of Co-citation References

#### 3.2.1 Clusters of Research and Most Cited Papers

The co-cited reference network reveals research trend evolutions using the structure and dynamic walkthrough of co-citation clusters. The time period was reduced by CiteSpace to 1978-2022 after the retrieval of empty intervals. The co-cited reference network that constitutes a single constellation of 21 clusters is shown in Fig. (**1**), and the time map visualization is shown in Fig. (**2**). The modularity score was significant (Q = 0.795), and the mean silhouette score suggested highly credible clusters (S = 0.915).

Based on the largest connected component of the network, these clusters organized from the largest (#0) to the smallest (#20) revealed four distinct major research trends. The first and most massive trend concerns the follow-up of a specific population, ‘war veterans and refugees’ (clusters #15, #1, #3, #11, #10, #17, #20, #4, and #14). The second trend regroups ‘treatment of PTSD’ (#8, #13, and #18) and ‘neuroimaging’ (#6, #9, #5, and #2), and the third is ‘evidence synthesis’ (#5, #0, and #7), and the last is a minor trend on ‘somatic symptoms of PTSD’ (#16, #19, and #12). The highlight of burstness with the detail of each cluster is represented in Supplementary Fig. (**S5**). A.B and as a video available on osf.io (link).

We subsequently detailed the cluster numbers (#), with the corresponding labels, cluster silhouette score (S), size (N), mean year (Y) of co-cited articles, and the most representative reference for each trend.

The first trend on the follow-up of a specific population, ‘war veterans and refugees’, started with the oldest identified cluster of the network, cluster #15 on ‘PTSD/Vietnam war’ (0.993; 80; 1982) [27], which continued with cluster #1 ‘PTSD review/Vietnam war’ (0.943; 342; 1989) [28]. The follow-up of war veterans continued for distinct conflict with #3 ‘population survey/Persian Gulf War’ (0.897; 317; 1996), #11 ‘terrorism 9/11/Afghanistan war’(0.92; 164; 2007) [1], #10 ‘veterans/Iraq war’ (0.944; 194; 2011) [29] and more recently war refugee, #17 ‘war refugee/Syria’(0.992; 57; 2019) [30], and #20 ‘psychosis/childhood trauma’ (0.995; 9; 2007) [31]. Finally, two recent and isolated clusters were found: one cluster on intensive care unit survivors, #14 ‘intensive care unit’ (0.971; 85; 2012) [32], and one on the COVID-19 pandemic, #4 ‘COVID-19’ (0.977; 236; 2020) [33].

The second major trend of research concerns treatments and neuroimaging of PTSD, starting with pharmacological treatment of PTSD, #8 ‘pharmacological treatment’(0.897; 233; 2002) [34], mainly co-citing research on antidepressants, cluster #13 ‘PTSD treatment’(0.919; 100; 2009) [35], with research on prazosin, beta blocker and cognitive behavioral therapy (CBT). These trends also have specific clusters on nonpharmacological treatments of PTSD, cluster #18 ‘noninvasive brain stimulation (NIBS)’(0.999; 14; 2000) [36], with a co-citation dynamic walkthrough to clusters of neuroimaging research. The latter starts with cluster #6 ‘affective neuroscience/magnetic resonance imaging (MRI)/ positron emission tomography (PET)’(0.914; 241; 2000) [37] and cluster #9 ‘functional neuroimaging’(0.908; 220; 2007) [38].

Co-citation dynamics merge this major trend with the latest massive trend on evidence synthesis, with cluster #5‘evidence-synthesis/fMRI’(0.863; 252; 2015) [39], and a massive cluster on the neuroscience of PTSD, #2 ‘evidence synthesis/neuroscience’(0.881; 317; 2016) [12]. More recently, this trend has developed in the most important cluster of the network, cluster #0 ‘evidence synthesis/gene-environment’ (0.842; 346; 2014) [40] and #7 ‘evidence synthesis/psychometric evaluation’(0.947; 240; 2017) [41].

Furthermore, one minor trend in somatic symptoms of PTSD was found, with clusters #16 ‘somatic symptoms/gulf war’ (0.974; 66; 1999) [42] and #19 ‘sleep disturbance/Vietnam veterans’ (0.999; 14; 1999) [43]. This trend shares co-citation dynamics with one last cluster on neuroendocrine aspects of PTSD with cluster #12 ‘hypothalamic-pituitary-adrenal axis (HPA)’ (0.932; 119; 2006) [44]. The timeline map visualization of the co-cited network reported in Fig. (**2**) shows that clusters #0 ‘gene-environmentenvironment’, #2 ‘PTSD mechanisms’, #4 ‘COVID-19’, #5 ‘evidence synthesis/fMRI’, #7 ‘evidence synthesis/psychometric evaluation’ and #17 ‘war refugee/Syria’ are currently active.

To retrieve the latest trends of research, we examined the 2020-2022 co-cited reference network (Supplementary Fig. **S6.A. B. C**). In addition to previously identified clusters, the most active clusters were those of the trend on ‘treatments of PTSD’, including both psychological approaches for PTSD with #9 ‘treatments/mHealth/virtual reality’ (0.981; 24; 2020) [45] and psychedelic treatment for PTSD, in particular 3,4-methylenedioxymethamphetamine (MDMA), ‘psychedelics’ (0.996; 38; 2021) [46]. Furthermore, for the trend in a specific population (“war veterans and refugees”), the distinct cluster on COVID-19 previously identified forms a unique novel trend composed of clusters related to COVID-19, with #2 ‘COVID-19/health care workers’ (0.749; 153; 2021) [47] and #4 ‘long covid’ (0.905; 85; 2021) [48]. Finally, this trend is also enriched with novel emerging clusters #6 ‘Complex PTSD/postpartum PTSD’(0.871; 75; 2021) [49] and #12 ‘grief disorders’(0.996; 3; 2020) [50].

#### 3.2.2 Most Co-cited Papers, Burstness Analysis, and Transformative Papers

The top 10 most co-cited papers are reported in Table **1**. The top three co-cited papers were the Blevins and colleagues (2015) PTSD checklist for DSM-V [41], the Bovin and colleagues (2016) paper on psychometric properties of the PTSD checklist for the DSM-V [51], and the Lai and colleagues (2020) cross-sectional study on mental health outcomes among health care workers during the COVID-19 pandemic [47].

The burstness analysis conducted for both co-cited reference networks also permits us to expose landmark literature. For the 1978-2022 network, the top three most recent co-cited papers with the most important burst strength were three papers on the COVID-19 pandemic [47, 51, 52] (Supplementary Table **S2.L**).

Furthermore, we conducted a structural variation analysis for January 2020 to June 2022 time period that focused on novel boundary-spanning connections to detect the best candidates for transformative papers (Supplementary Table **S3**). The top three transformative papers presenting the strongest modularity divergence scores that introduced novel connections were reported byZhang and colleagues in 2022 paper on the genetic correlation between PTSD and depressive phenotypes [53], the Ettorre and colleagues 2020 review, and the Chatziottofis cross-sectional study, both on the impact of the COVID-19 pandemic on the mental health of health care workers [54, 55].

### 3.3 Analysis of Co-occurrence Keywords

**Fig. (3):**
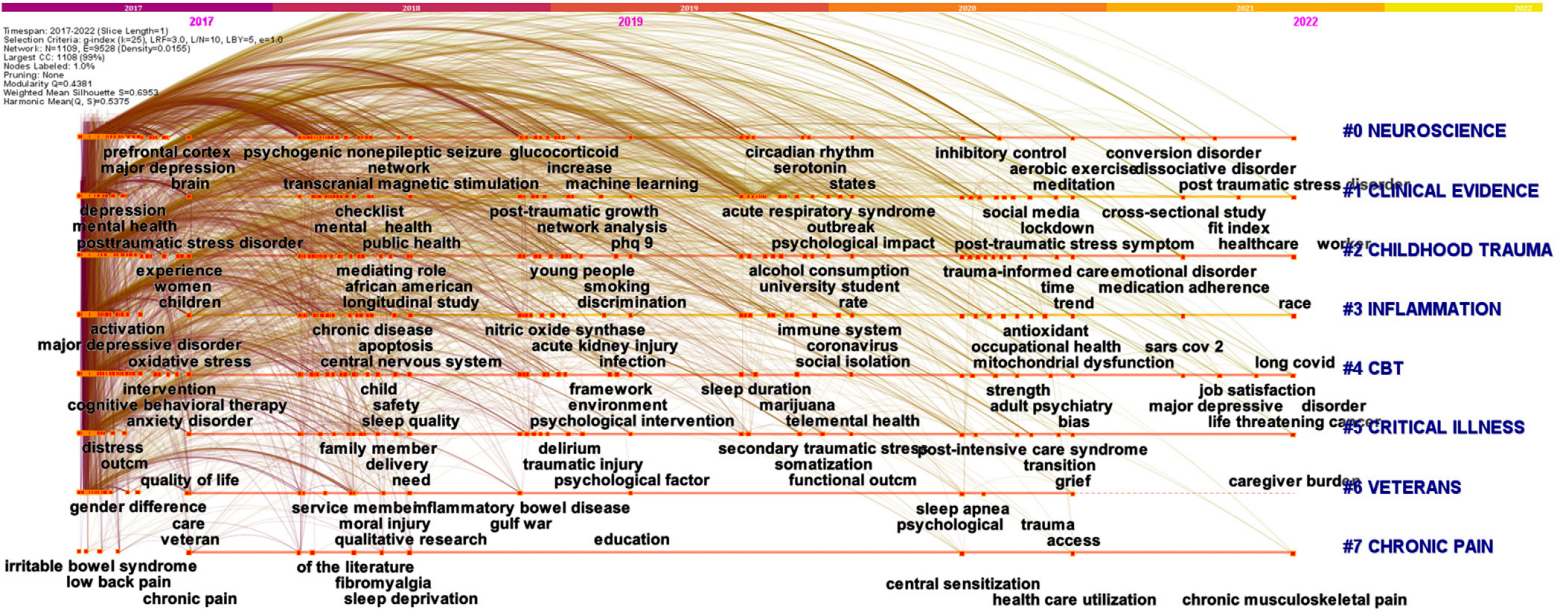
Co-occurrence keywords network (2017-2022 period). The nodes represent keywords, and the colors show the average year of publication for each node. The size of a node is proportional to the burstness of keyword co-occurrence. The co-occurrence network is weighted on total link strength across different keyword nodes and scored on the average publication years. The clusters are labeled in blue at the far right of the timeline maps.

The co-occurrence keyword network helps to uncover meaningful knowledge components and insights based on the patterns and strength of links among keywords that appear in the literature. We retrieved the co-occurrence network for the last five years (2017-2022 time period) to reveal the latest trends of research (Fig. **3**). The modularity score was significant (Q = 0.438), and the mean silhouette score suggested highly credible clusters (S = 0.695). Eight different clusters were identified, and the top three clusters were cluster #0 ‘neuroscience’ (0.71; 236; 2017), #1 ‘clinical evidence’ (0.677; 214; 2018), and #2 ‘childhood trauma’ (0.6; 194; 2018). The burstness analysis revealed that the top three terms with the most important strength of burst mainly concerned the COVID-19 pandemic, including ‘psychological impact’ (30.62), ‘acute respiratory syndrome’ (27.03), and ‘sar’ (23.6) (Supplementary Table **S2. J**).

### 3.4 Analysis of Cooperation Across Countries, Institutions, and Authors

We identified 115 different author countries in our dataset. The three countries gathering the most important number of articles were the USA (n = 20,156), the United Kingdom (n = 4,518), and Germany (n = 3,334). China was in the 6^th^ position, with exponential growth in the number of papers published in the last 10 years (n = 2,243) (Supplementary Table **S4**).

**Fig. (4):**
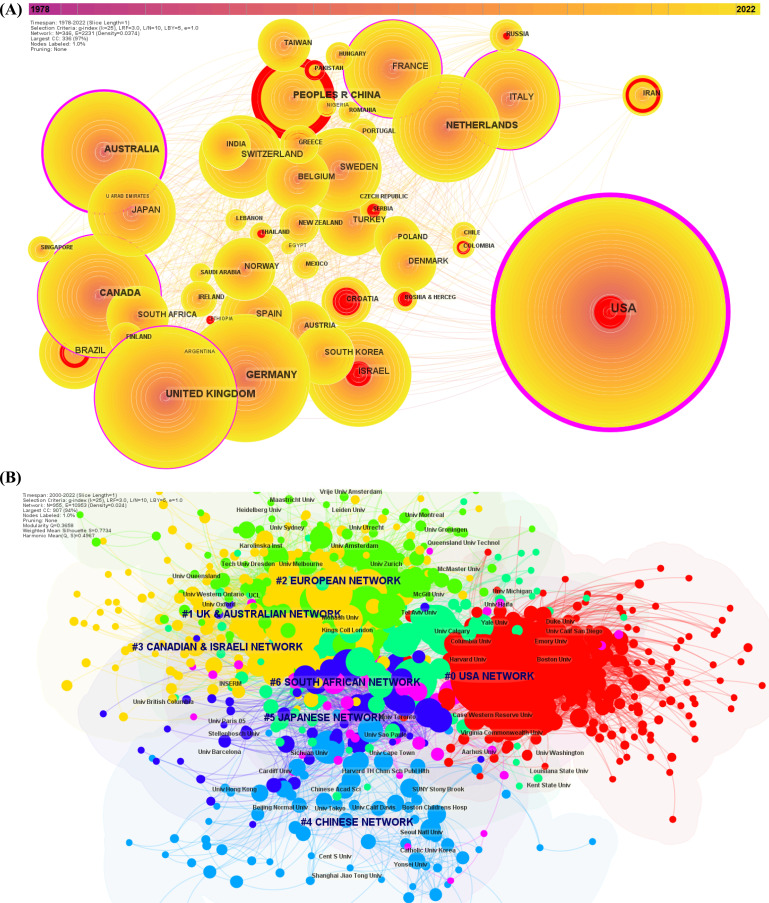
Network of co-authors' countries (**A**) (1978-2022) and network of co-cited authors institution (**B**) (2000-2020). (**A**) The network of co-authors countries reveals the collaborative country network. Betweenness centrality organizes the network, with the countries presenting the most important centrality being central to the network. Centrality is represented with a peripheral pink trim which thickness is proportional to the degree of centrality. Highly central nodes are considered pivotal points in the research field. We limit the nodes to the 50 first countries. (**B**) Visualization map of the network of co-author institutions with the highlight of clusters. Each node represents an institution. Each cluster gathers several institutions that constitute a research network. The thickness of links between nodes is proportional to the intensity of collaboration and denotes of the influence of each node.

We retrieved the co-cited country network based on the country of each author (Fig. **4A**). The countries with the highest degree of centrality were the USA (0.73), Australia (0.24), and the United Kingdom (0.17). The burstness analysis revealed that the USA (57.25; 1981-1993), Israel (21.05; 1986-2002) and Croatia (20.06; 1999-2012) had the earliest and most important co-citation bursts. Furthermore, the three countries with the most recent strength of co-citation burst were China (149.84; 2020-2022), the USA (57.25; 1981-1993), and Croatia (20.06; 1999-2012).

The cooperation networks across 955 institutions for the last two decades were also retrieved. The top three institutions gathering the most important number of articles were the US Department of Veterans Affairs (n = 3,954), Harvard University (n = 2,534), and the University of California (n = 2,296), and those with the most co-cited articles were Harvard University, Columbia University and Yale University (Supplementary Table **S4**).

The co-cited institution network of the last two decades presented a significant modularity score that was significant, and the mean silhouette score indicated highly credible clusters (Q = 0.496; S = 0.773). Six different clusters were identified, with the most massive cluster being #0 ‘USA network’. European countries shared more cooperation with Australian and Canadian institutions (#1 ‘UK & Australian network’, #2 ‘European network’ and #3 ‘Canadian & Israeli network’), and cluster #4, ‘The Chinese network’, was more peripheral to the previously cited clusters (Fig. **4B**).

Finally, the focus on the co-authorship network can be considered a social and collaborative network encompassing researchers. The co-authorship network of the last two decades presenting a significant modularity score was significant, and the silhouette score indicated highly credible clusters (Q = 0.895; S = 0.769). In total, 13 different clusters of collaborating circles were found, giving insight into specific research topics #0 ‘COVID-19’, #1 ‘war veterans’, #2 ‘PTSD mechanism’, #3 ‘police’, #4 ‘HPA AXIS’, and #5 ‘complex PTSD’ (Supplementary Fig. **S7**). The burstness analysis revealed that the top three authors with the most important burstness for co-authorship citation burst were ‘Davidson J’ (34.53), ‘Yehuda R’ (32.04) and ‘Charney D’ (25.81) (Supplementary Table **S2. H**).

## 4 DISCUSSION

### 4.1 Summary of the Main Findings

Our scientometric analysis proposes a snapshot of the current state of knowledge and highlights how evidence is connected, uncovering the structure and development of PTSD research. This overview of the literature on PTSD has included 42,170 articles with an exponential growth of publications since 1980.

The USA is the most influential country, with the most important number of published papers and citations and with only American institutions in the top 10 most influential institutions. Of importance, China presented in the last year a significant burst of both publications and citations. Schematically, among the four major trends of research, we identified distinct clusters linked to war-related trauma and more recent ones linked to civilian trauma. All retrieved networks presented significant modularity scores with highly credible silhouette scores. These different research clusters reflect the geopolitical events and major disasters of the past decades that directly drive PTSD research and promote the integration of the latest scientific research advances.

### 4.2 Evolution of Research Trends for War-related Trauma

The Vietnam War veteran’s follow-up drove the first decades of research. The Vietnam War (1955-1975) media coverage constituted a real traumatism for both the US Army and the American people facing the brutality of war for the first time. This media coverage participated in stigmatizing the surviving soldiers of the conflict and induced a ‘cultural trauma’ to the American population [56]. Nevertheless, it also strengthened funding for the conduction of cohort follow-ups that have provided a solid foundation for clinical research, such as the National Vietnam Veterans Readjustment Study, revealing 15 to 30% of the lifetime prevalence of PTSD for veterans [57].

After this initial focus on Vietnam survivors, new conflicts appeared, such as the Gulf War (#3) in 1990-1991 [58], with exposure to sundry traumatic stressors, and the emergence of a specific physical syndrome (#16), with irritable bowel syndrome and the ‘Gulf War illness’ [59]. Major advances were made during these decades with the development of structural neuroimaging [37, 38], which allowed the identification of structural abnormalities associated with PTSD, such as a smaller hippocampus, and novel nonpharmacological treatments, such as rTMS [36].

In the following decades, the Afghanistan war (#11) 2001-2021 and the Iraq war clusters (#10) in 2003-2011 appeared. Adjacently, the development of functional neuroimaging (#5) laid the groundwork for translational research for PTSD [39]. However, with the recent end of the Afghanistan war, this major trend of research has become less active.

The evolution of remote-warfare tactics has also changed the types of trauma. Although some soldiers might be less physically exposed to battlefields, novel types of war-related trauma are found in drone operators [60], with a lower prevalence of PTSD found in this population compared to one of the soldiers exposed to the battlefield [61].

### 4.3 Evolution of Research Trends for Civilian Trauma

Civilian populations have always been the first victims of war-related trauma but also of major disasters and pandemics.

Nevertheless, the first scientific papers focusing on PTSD we identified in Holocaust survivors were not published until 1988 [62]. In Cluster 3 of the co-authors’ network, we see a burst of citations for the work of some authors on Holocaust survivors [63]. Furthermore, the first co-cited reference clusters identified for civilians are cluster #11 ‘terrorism9/11/Afghanistan war’, and #20 ‘psychosis/childhood trauma’, with a focus on firefighters and childhood trauma, respectively.

Terrorism can have a strong impact on civilian health, with at least as many occurrences of PTSD as war-related PTSD [64]. Of importance, poorly co-cited trends do not necessarily appear in the co-cited network. Therefore, we could still find many clusters regarding terrorism attacks, such as those related to the Paris terrorist attack of 13 November 2015 or the 16 April 2014 Sewol ferry disaster (Supplementary Fig. **S7**).

Some clusters linked to the development of trauma related to physical health consequences have also appeared, such as cluster #14 ‘intensive care unit’. The focus on the most recent research trends using the 2020-2022 co-cited reference network revealed other emerging clusters, such as #6 ‘Complex PTSD/post-partum PTSD’ or #16 ‘(prolonged/complicated) grief disorder’, witnessing the development of research outside of the war-related context.

Furthermore, as found in many scientometric studies, the COVID-19 pandemic has largely marked clinical research. In the context of PTSD, the #4 COVID-19 cluster can be detailed in several smaller clusters in the 2020-2022 network, with clusters #1 ‘COVID-19/psychological distress’, #2 and #12 on ‘COVID-19/health care workers’, and #4 ‘long covid’.

In line with this, we found that, among the top 10 articles with the strongest centrality divergence scores between 2020 and 2022, seven concerned the impact of COVID-19 on mental health, confirming that the pandemic strongly transformed and expanded PTSD research to further clinical and demographic aspects (Supplementary Table **S3**).

Similar to the #17 cluster, the recent war in Ukraine represents one of the largest refugee migratory crises in recent European history, with at least 15 million people displaced. Given the proximity of the event, no cluster on this event resulted from our analysis. Nevertheless, the Ukrainian refugee crisis can be seen as one of the largest refugee migratory crises in recent European history, with the need for host countries to develop public mental health programs able to reduce the long-term impact of trauma [65]. The first results confirm a strong exposure to traumatic events and a high prevalence of PTSD symptoms among urban-dwelling persons and, in particular, internally displaced persons [66]. Our mental health intervention should include acute mental health first aid, preventive intervention, and specific education programs that could even benefit other asylum seekers [67]. Furthermore, research has been conducted on the impact of the post-migrational environmental stressors (legal status, postmigration living conditions) on the prognosis of PTSD symptom evolution [68].

Indeed, the number of war refugees, asylum migrants, and economic migrants has greatly increased; moreover, climate change migrants are projected to cause substantial increases in population movement in the coming decades [69].

### 4.4 Evidence Synthesis Trend Leading Current PTSD Research

In parallel with developments in both war-related and civilian-related trauma, it is the ‘evidence synthesis’ trend that currently dominates research, with a cluster on translational research (#0) on evidence synthesis regarding genetic predisposition and environmental exposures for the occurrence of PTSD [40] and the search for inflammatory markers [70].

Indeed, one of the main features of PTSD is that it develops only in a small part of the population exposed to the trauma. Genetic background, as well as early-life experiences, are major players in developing resiliency or vulnerability in response to traumatic exposure. In particular, the gene x environment interaction seems to negatively affect resilience to trauma (Klengel 2013; Zannas & Binder 2014; Klengel & Binder 2015). Importantly, mutation of the FKBP5 gene [71], which is a major regulator of glucocorticoid receptor (GR) sensitivity, has been linked to a higher risk of developing PTSD, especially in individuals who experience childhood abuse (Binder *et al.* JAMA 2008) or war scenarios [72].

In addition, the results of evidence synthesis concerning the field of fMRI and neuroscience (clusters #5 and #2, respectively) can explain most of the current PTSD research. Whereas advances in neuroimaging techniques have made the identification of structural and functional brain abnormalities linked to PTSD vulnerability possible (Gilbertson *et al.* 2002, Pitman *et al.*, 2012), research on the neurocircuitry of fear memory and fear extinction in the past 20 years has allowed a deep understanding of the neurobiology of this disorder [73]. However, the same progress has not yet been equally translated into psychiatric practice for PTSD, and this remains one of the major challenges in the field.

### 4.5 Evolution of PTSD Treatments

Pharmacological treatments for PTSD (cluster #8) have been tested since 2000, particularly with antidepressants [34], and the primary prevention of PTSD has also been studied with propranolol [74]. Later, secondary prevention of PTSD with pharmacotherapy in the aftermath of the trauma was proposed. Prazosin, as a treatment for trauma nightmares and sleep disturbances [35], and morphine, as a treatment for controlling pain and anxiety after injury [75], were both associated with a reduction in PTSD development. Nonpharmacological interventions have also been developed, with NIBS focusing on the right dorsolateral cortex, appearing to have a positive effect in reducing core symptoms in patients with posttraumatic stress disorder [76]. Furthermore, some randomized trials are available for cognitive behavior therapy (trauma-focused CBT and exposure-based therapy), which is considered the gold standard treatment for childhood, adolescent, and adult PTSD [73] given its ability to modify neural networks [77].

Moreover, the 2020-2022 network reveals the important development of PTSD treatments. The psychedelics renaissance (cluster #8) is currently promoting the use of MDMA-assisted therapy [46] for PTSD. In addition, the use of mHealth apps and virtual reality (cluster #9) is becoming more popular as a complementary treatment for PTSD, often in association with exposure therapy [78, 79]. Preliminary randomized control studies also show a beneficial effect of hydrocortisone on a specific population of PTSD patients [80, 81]. Finally, another promising treatment is the use of synthetic cannabinoids, such as nabilone, particularly for PTSD-associated nightmares [82].

### 4.6 Somatic Symptoms or Biological Features of PTSD

A specific cluster on sleep disturbances related to the Vietnam war (#19) resulted from the analysis [83]. This is not surprising, as sleep impairments, such as recurrent nightmares and sleep discontinuity, are diagnostic criteria of PTSD and severely affect daily functioning (Neylan *et al.* 2006). In addition, the HPA/PTSD cluster (#12) is also part of the research trend on somatic symptoms of PTSD. This is intuitive, as traumatic experiences are stressful events; thus, they are likely to trigger the neuroendocrine release of the stress hormone. In parallel, this might be linked to the abundant literature showing that low rather than elevated cortisol levels are usually found in PTSD patients (Yehuda *et al.* 1995, Meewisse *et al.* 2007). Notably, data from recent studies have suggested that this phenotype may reflect a preexisting vulnerability trait rather than being a consequence of the trauma exposure [84].

### 4.7 Influence Networks and Changes in Policy

The retrieved collaborative network clearly shows that American institutions are leading the research on PTSD, particularly for war-related trauma. We can assume that this is partly due to the productivity of scientific research in the USA but also to the intense military activity of the US army in recent decades. Nevertheless, civilian-related trauma has been extensively explored lately, with important advances being made for the different civilian populations exposed to the risk of developing PTSD. One important gap in research is the lack of large-scale investigations of the consequences of various sources of trauma (climate change, disasters, and war conflicts) in developing countries or third-world countries. As a consequence, important disparities in PTSD treatment exist based on the country income level. In a recent survey from the World Mental Health (WMH), individuals from high-income countries were twice as likely to seek treatment as those in low-lower and upper-middle-income countries [85].

Nevertheless, the very nature of these conflicts and political aspects plays a very large role in the financing of research. Another important gap is - compared to other psychiatric disorders- a lack of high-quality randomized controlled trials for pharmacological and nonpharmacological treatments, which has limited treatment guidelines for PTSD.

### 4.8 Limitations

One of the most important limitations of scientometric analysis is the use of citation-related indicators that can expose citation bias: the probability of being cited depends on the outcome of that study [86]. Of importance, research trends that are poorly co-cited or constituted by recently published articles do not necessarily appear in the co-cited network, although emerging trends could be very impactful in the future direction of research. Self-citations, the authority of the author, and the journal impact are different parameters that can influence citation bias. The use of WOSCC as the only database can further limit the interpretation of results [87]. However, most databases (*e.g*., PubMed, EMBASE, and PsyINFO) do not propose full references and citations of articles. Furthermore, most DOIs differ between WOS and Scopus, which has greatly limited the merging of datasets.

## CONCLUSION AND FUTURE DIRECTION

This scientometric analysis provides historical insight and future perspectives on clinical research concerning PTSD. Important progress in research on PTSD has been made since the Vietnam War. The study of PTSD neurobiology and cognitive mechanisms has helped the development of specific therapies and continues with innovative treatments. Nevertheless, tremendous challenges remain with the need for effective PTSD scaled-up interventions and optimal treatment for war-related and, in particular, civilian-related trauma and the handling of the increasing migration of populations due to war conflict, economic migration, and possibly climate change. This study provides a comprehensive overview of research on PTSD over the past 50 years, informing future directions for researchers, grant applicants, and policy-makers.

## AUTHORS’ CONTRIBUTIONS

Conceptualization was done by Michel Sabe, Stefan Kaiser, and Marco Solmi. Methodology was conducted by Michel Sabe, Chaomei Chen, and Marco Solmi. Formal analysis and investigation were performed by Michel Sabe, Chaomei Chen, and Marco Solmi Writing and original draft preparation were done by Michel Sabe, Stefan Kaiser, and Marco Solmi. Writing - review, and editing were contributed by all authors equally,under the supervision of Stefan Kaiser and Marco Solmi.

## Figures and Tables

**Fig. (1):**
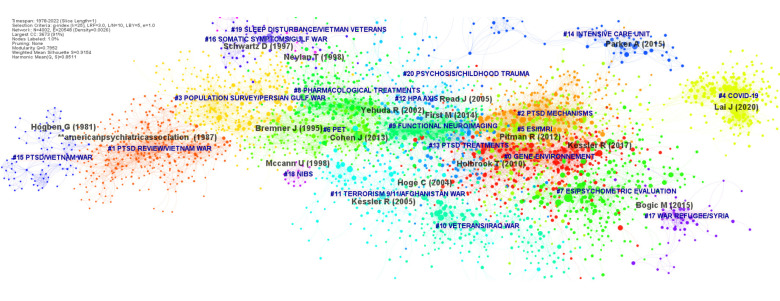
Co-cited reference network with highlight of clusters (1978-2022 period). The position of the node (article) corresponds to the year of publication. The size of a node is proportional to the number of times the node has been co-cited. For each cluster, a single color is attributed. Each cluster is arranged on a horizontal timeline from left (1978) to right (2022). In Figure B, colors are attributed to the four different trends identified: I ‘war veterans and refugees’ (blue); II ‘treatment of PTSD’ and ‘neuroimaging’ (green); III ‘evidence synthesis’(orange); IV ‘physical symptoms of PTSD’(brown).

**Fig. (2):**
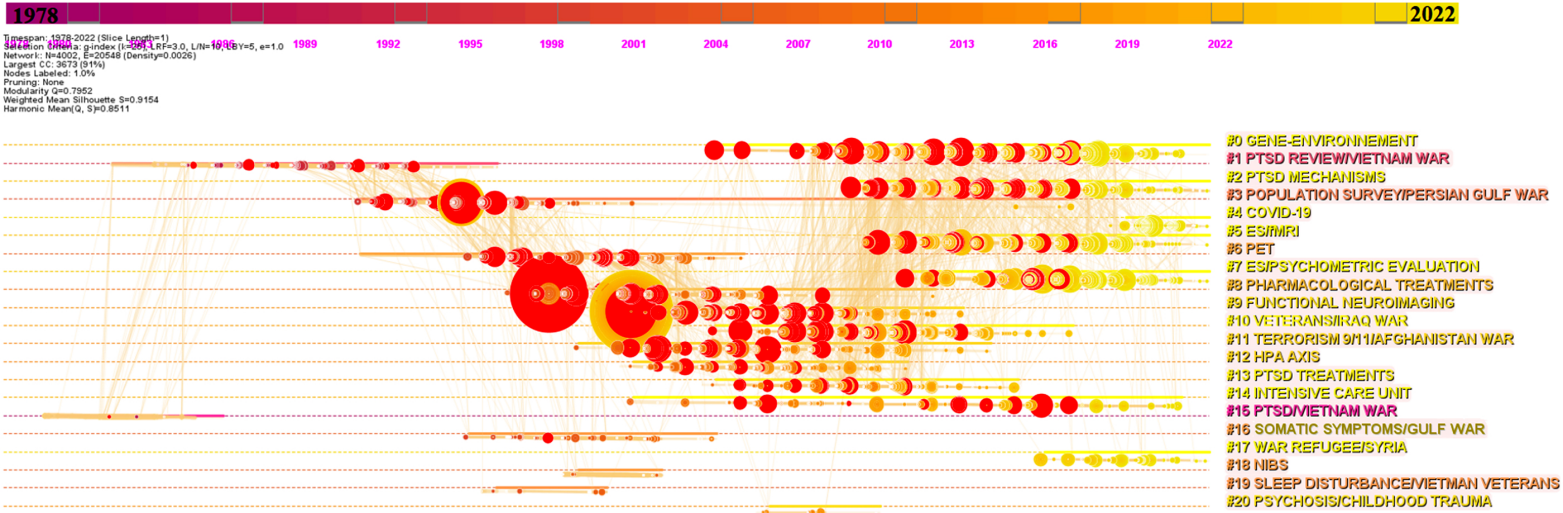
Timeline map visualization of the co-cited network (1978-2022 period). The position of the node (article) corresponds to the year of publication. The size of a node is proportional to the number of times the node has been co-cited. The cluster are ranked by their size from #0 to #20. The clusters are labeled at the far right of the timeline maps. Both nodes, links between nodes and clusters labels receive a color associated to the horizontal line from left (purple) to right (yellow) to inform on the mean year of publication or co-citation. Burstness of citation is revealed with the presence of red tree rings that superimpose the previous node color. The thicker the red tree rings, the more burstness for the corresponding node.

**Table 1:** The top 10 most co-cited references.

**Number of ** **Co-citations in the Network/Number of Citations in the Literature^a^**	**Source**	**Vol**	**Page**	**Title**	**Doi**	**Type of Study**	**Related Cluster in Fig 1**
457/3034	J Trauma Stress	28	489-98	Blevins *et al.* 2015. The Posttraumatic Stress Disorder Checklist for DSM-5 (PCL-5): Development and Initial Psychometric Evaluation	10.1002/jts.22059	Checklist	0
292/6573	JAMA Netw Open	3	3	Lai *et al.* 2020. Factors Associated With Mental Health Outcomes Among Health Care Workers Exposed to Coronavirus Disease 2019	10.1001/jamanetworkopen.2020.3976	Cross-sectional	4
286/1665	Psychol Assess	28	1379-91	Bovin *et al.* 2016. Psychometric properties of the PTSD Checklist for Diagnostic and Statistical Manual of Mental Disorders-Fifth Edition (PCL-5) in veterans	10.1037/pas0000254	Clinical study	7
216/14054	The Lancet	395	912-20	The psychological impact of quarantine and how to reduce it: rapid review of the evidence	10.1016/S0140-6736(20)30460-8	Review	4
203/23226	Arch Gen Psychiatry	62	593-02	Kessler *et al.* 2005. Lifetime prevalence and age-of-onset distributions of DSM-IV disorders in the National Comorbidity Survey Replication	10.1001/archpsyc.62.6.593	Household survey	11
202/1335	Nat Rev Neurosci	13	769-87	Pitman *et al.* 2012. Biological studies of post-traumatic stress disorder	10.1038/nrn3339	Review	9
195/14223	Arch Gen Psychiatry	52	1048-60	Kessler *et al.* 1995. Posttraumatic stress disorder in the National Comorbidity Survey	10.1001/archpsyc.1995.03950240066012.	Household survey	6
180/6623	N Engl J Med	351	13-22	Hoge *et al.* 2004. Combat Duty in Iraq and Afghanistan, Mental Health Problems, and Barriers to Care	10.1056/NEJMoa040603	Review	11
174/796	Psychol Assess	30	383-95	Weathers *et al.* 2018. The Clinician-Administered PTSD Scale for DSM-5 (CAPS-5): Development and initial psychometric evaluation in military veterans	10.1037/pas0000486	Clinical study	7
172/8893	Int. J. Environ. Res. Public Health	17	1729	Wang *et al.* 2020. Immediate Psychological Responses and Associated Factors during the Initial Stage of the 2019 Coronavirus Disease (COVID-19) Epidemic among the General Population in China	10.3390/ijerph17051729	Online survey	4

^a^Number of citations in the literature 9according to the journal where the paper was published.

## LIST OF ABBREVIATIONS

CBT: Cognitive Behavioral Therapy
COVID-19: Coronavirus 2019 Disease
GR: Glucocorticoid Receptor
mPFC: Medial Prefrontal Cortex
PET: Positron Emission Tomography
PTSD: Posttraumatic Stress Disorder
