# Supplementary material for: Half a Century of Research on Posttraumatic Stress Disorder: A Scientometric Analysis
Source: Curr Neuropharmacol. 2023 Sep 28;22(4):736–48. doi: 10.2174/1570159X22666230927143106 (PMC10845098; doi:10.2174/1570159X22666230927143106)
Supplement: Supplementary file 1 [file CN-22-736_SD1.pdf]

## Supplementary Material

# Half a Century of Research on Posttraumatic Stress Disorder: A Scientometric Analysis

Michel Sabé[1], Chaomei Chen[2], Wissam El-Hage[3], Arnaud Leroy[4], Guillaume Vaiva[5], Silvia Monari[1], Natacha Premand[1], Javier Bartolomei[1], Stefano Caiolo[6], Andreas Maercker[7], Robert H. Pietrzak[8,9], Marylène Cloître[10], Stefan Kaiser[1,*,#] and Marco Solmi[11,12,13,14,15,#]

*[1]Division of Adult Psychiatry, Department of Psychiatry, University Hospitals of Geneva, 2, Chemin du Petit-Bel-Air, CH-1226, Thonex, Switzerland; [2]College of Computing & Informatics, Drexel University, Philadelphia, PA, USA; [3]CHRU de Tours, Clinique Psychiatrique Universitaire, Centre Régional de Psychotraumatologie CVL, 37540 Saint-Cyr-sur-Loire, France; UMR 1253, iBrain, INSERM, Université de Tours, 37000 Tours, France; [4]Univ Lille, INSERM, Lille Neuroscience & Cognition Centre (U-1172), Plasticity & SubjectivitY Team, CHU Lille, Fontan Hospital, General Psychiatry Department & Centre National de Ressources et Résilience Pour les Psychotraumatismes (CN2R Lille - Paris), 59000 Lille, France; [5]CNRS UMR 9193-PsyCHIC-SCALab, & CHU Lille, Department of Psychiatry, Univ. Lille, F-59000, Lille, France; [6]Department of Neuroscience (DNS), University of Padova, Padua, Italy; [7]Division of Psychopathology and Clinical Intervention, University of Zurich, Zurich, Switzerland; [8]Department of Psychiatry, Yale University School of Medicine, New Haven, CT, USA; [9]US Department of Veterans Affairs National Center for Posttraumatic Stress Disorder, VA Connecticut Health Care System, West Haven, Connecticut; [10]National Center for PTSD Dissemination and Training Division, VA Palo Alto Health Care System, USA; and Department of Psychiatry and Behavioral Sciences, Stanford University, USA; [11]Department of Psychiatry, University of Ottawa, Ontario, Canada; [12]Department of Mental Health, The Ottawa Hospital, Ontario, Canada; [13]Ottawa Hospital Research Institute (OHRI) Clinical Epidemiology Program University of Ottawa, Ontario, Ottawa; [14]School of Epidemiology and Public Health, Faculty of Medicine, University of Ottawa, Ottawa, Canada; [15]Department of Child and Adolescent Psychiatry, Charité Universit¨atsmedizin, Berlin, Germany*

**Supplementary Information 1.** CiteSpace general parameters

**General CiteSpace Parameters used for Analysis:**

CiteSpace parameters were as follows:

- Link retaining factor of 3.0
- Look back years (−1)
- Time span (1950-2022) with one slice per year
- Links (strength: cosine, scope: within slices)
- Selection criteria (g-index scale factor of 25)
- Minimum duration set to 5 years
- Burstness analysis for the 1950-2022 network set to 5 years, to 2 years for the 2016-2022, and to 0 years for the 2021 analysis.

**1875-6190/24 $65.00+.00** **© 2024 Bentham Science Publishers**

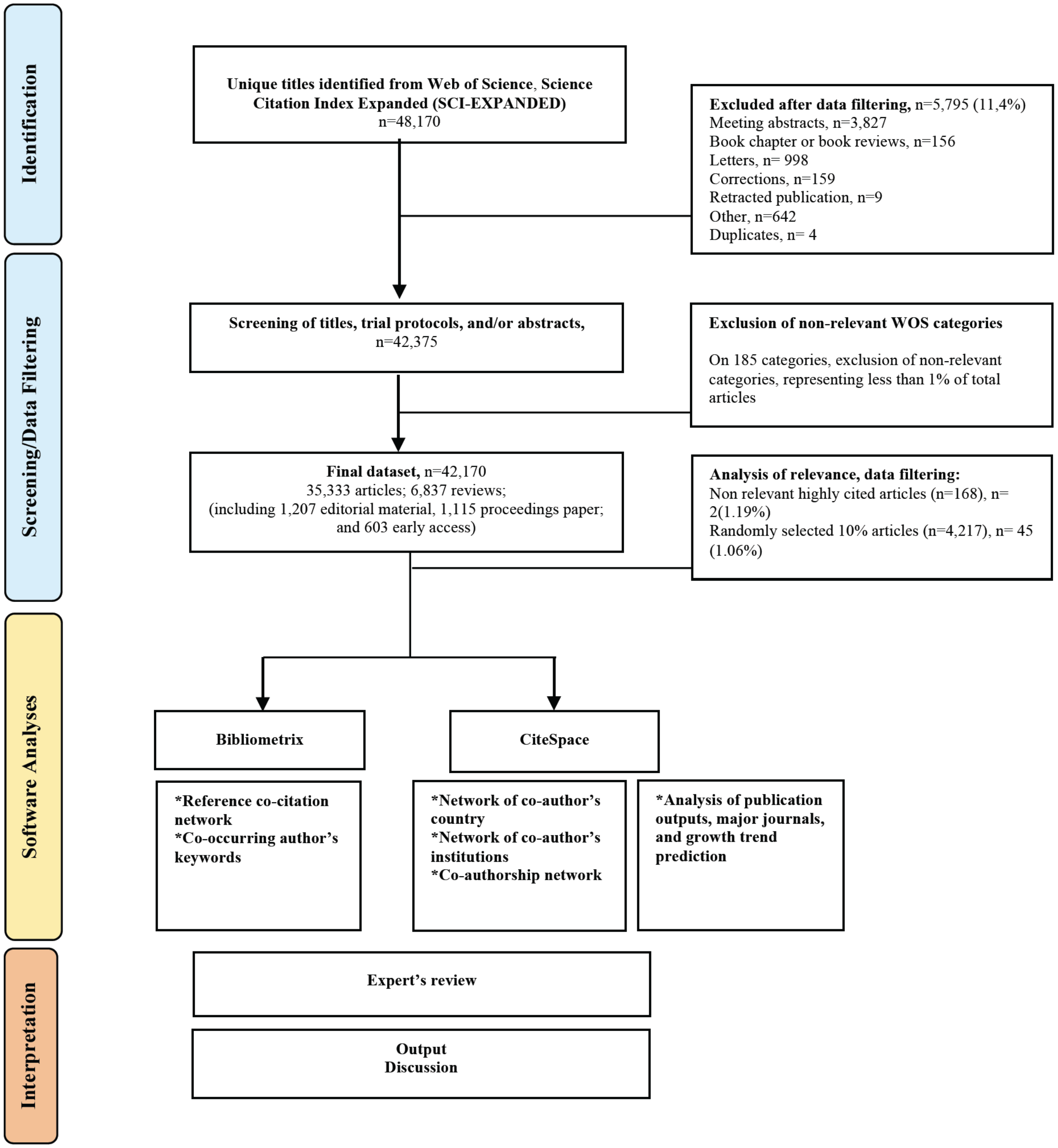


**Supplementary Fig. (1).** Flow chart of the scientometric stud.

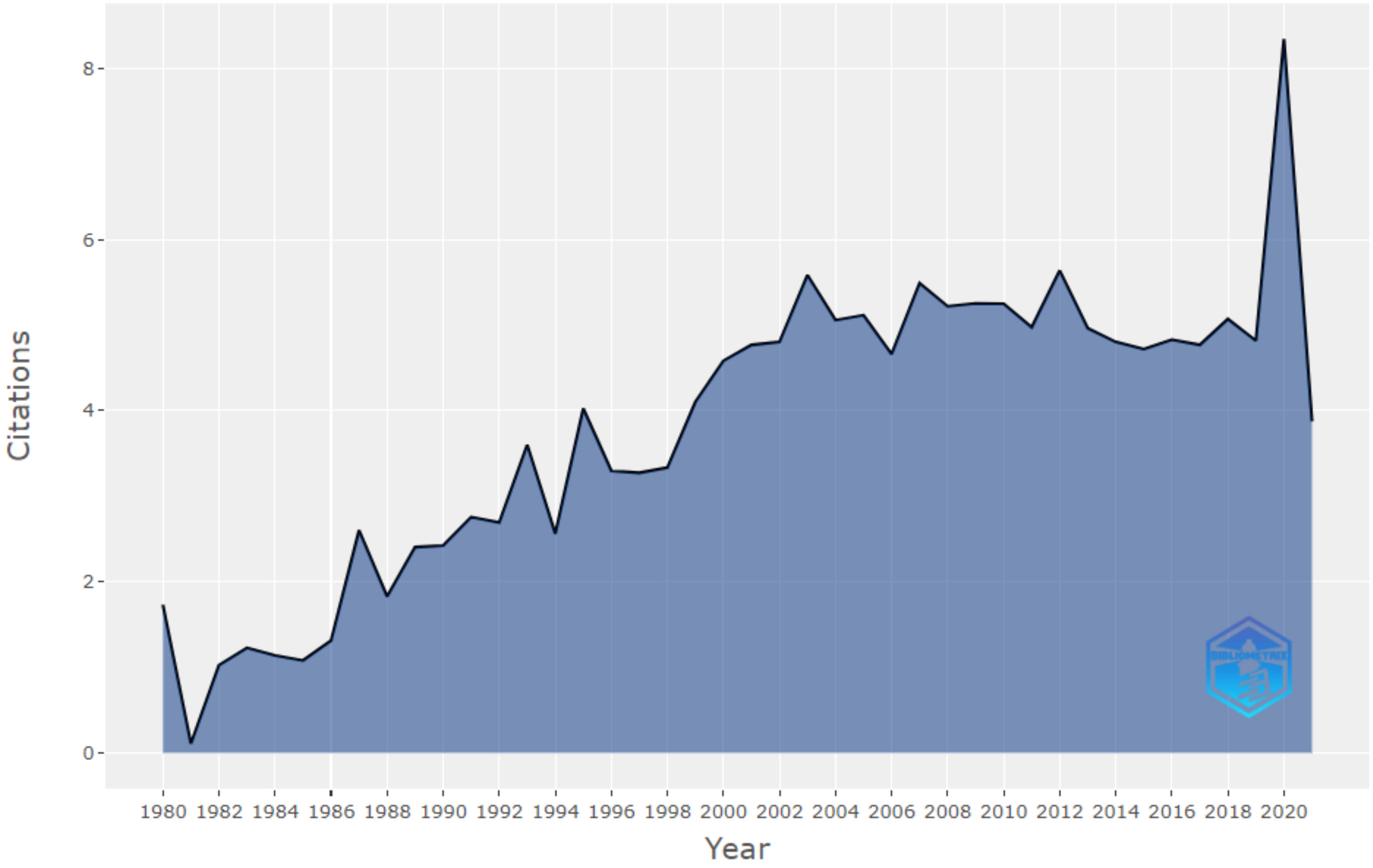


**Supplementary Fig. (2).** Average citation per year for references (1980-2022).

*Mean citation per article and per year: 1980: 1.7; 1985: 1.1; 1990: 2.4; 1995: 4; 2000: 4.6; 2005: 5.1; 2010: 5.2; 2015: 4.7; 2020: 8.3*

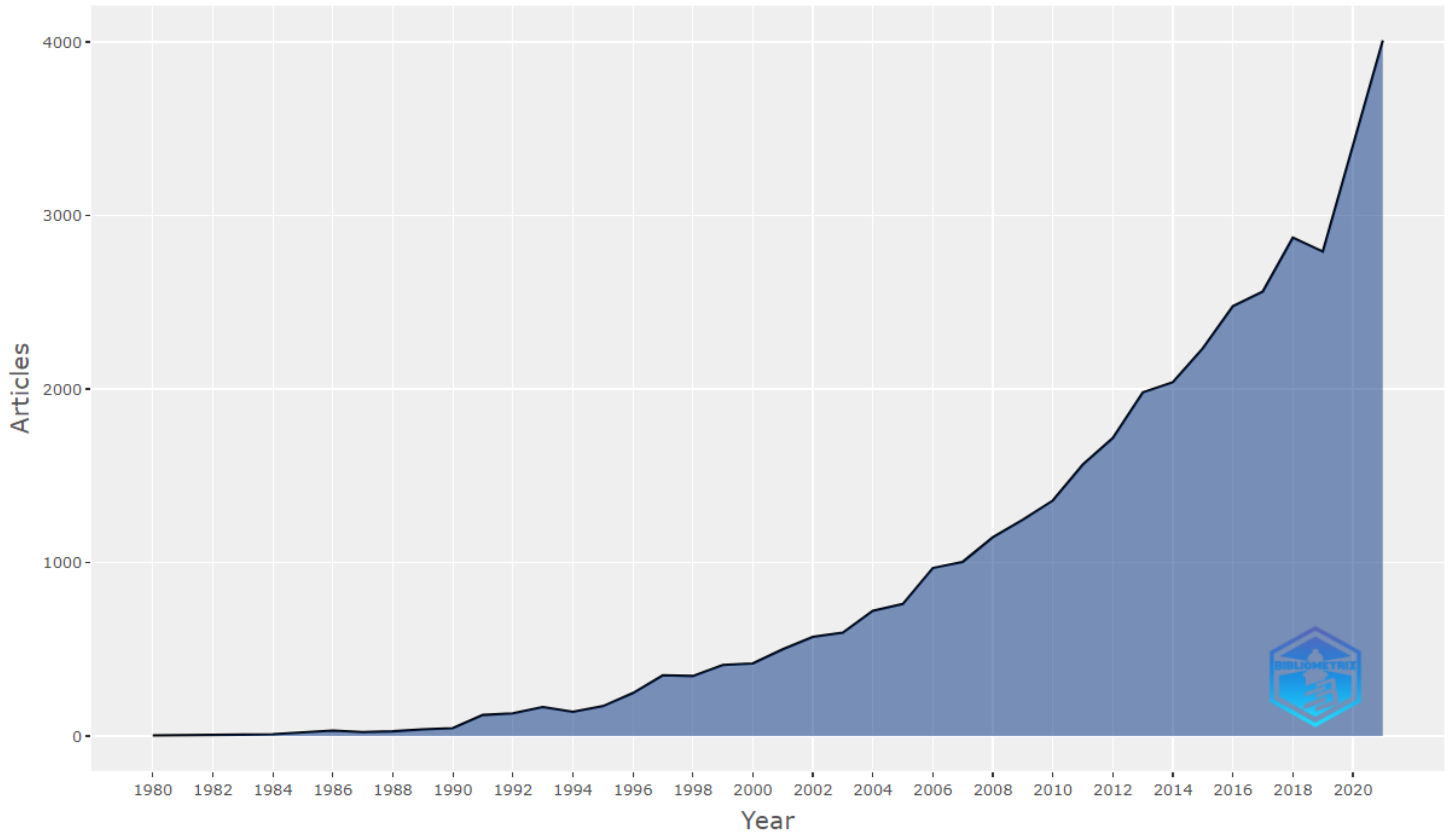


**Supplementary Fig. (3).** Annual scientific production (1980-2022),

*Number of articles per year: 1980: 3; 1985: 21; 1990: 45; 1995: 172; 2000: 418; 2005: 761; 2010: 1357; 2015: 2234; 2020: 3406; 2021: 4009*
*Annual Growth Rate: 19.19%*

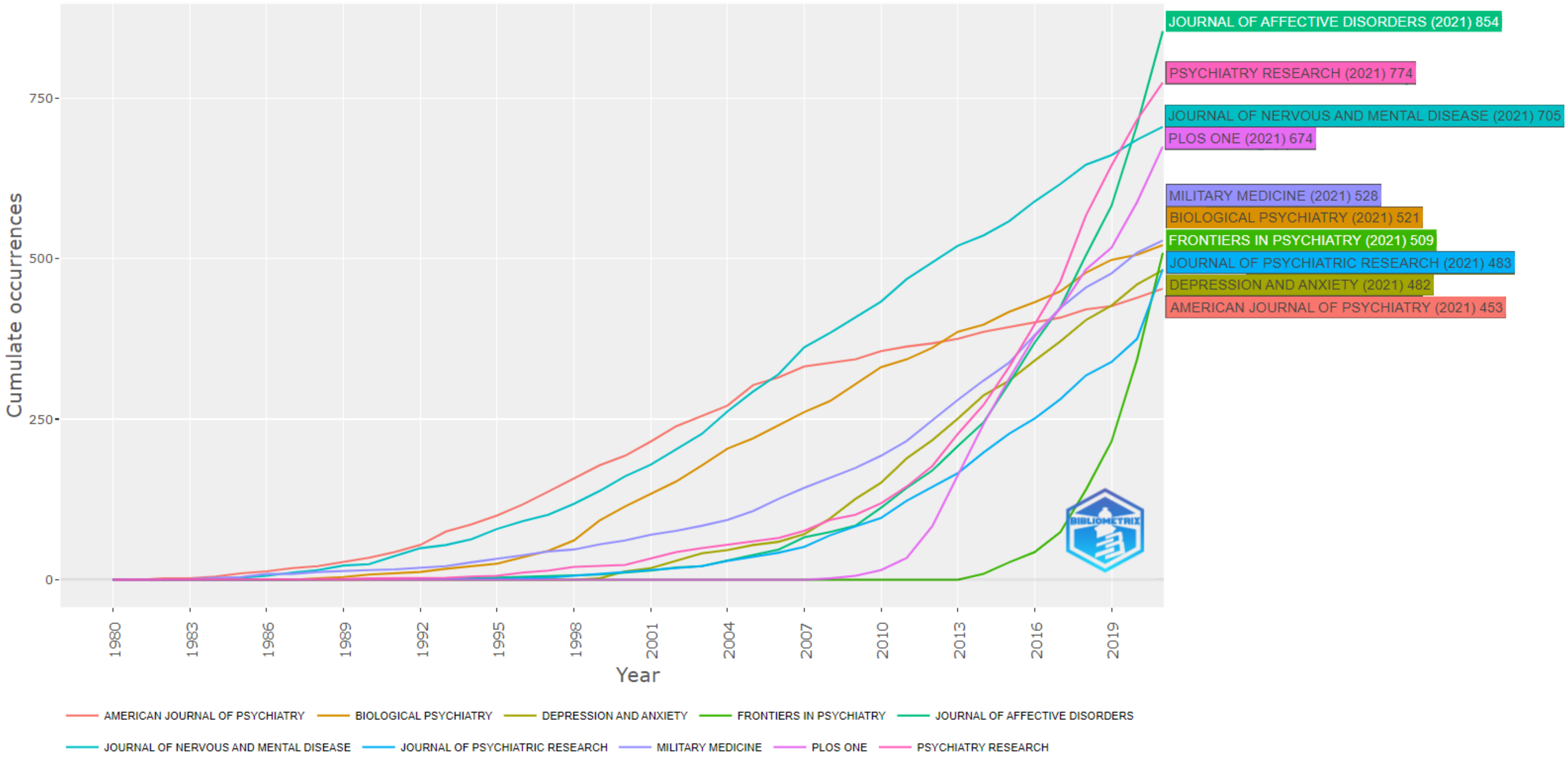


**Supplementary Fig. (4).** Source growth of top 10 journals.

**A. Co-citation reference network with highlight of burstness (1978-2021)**

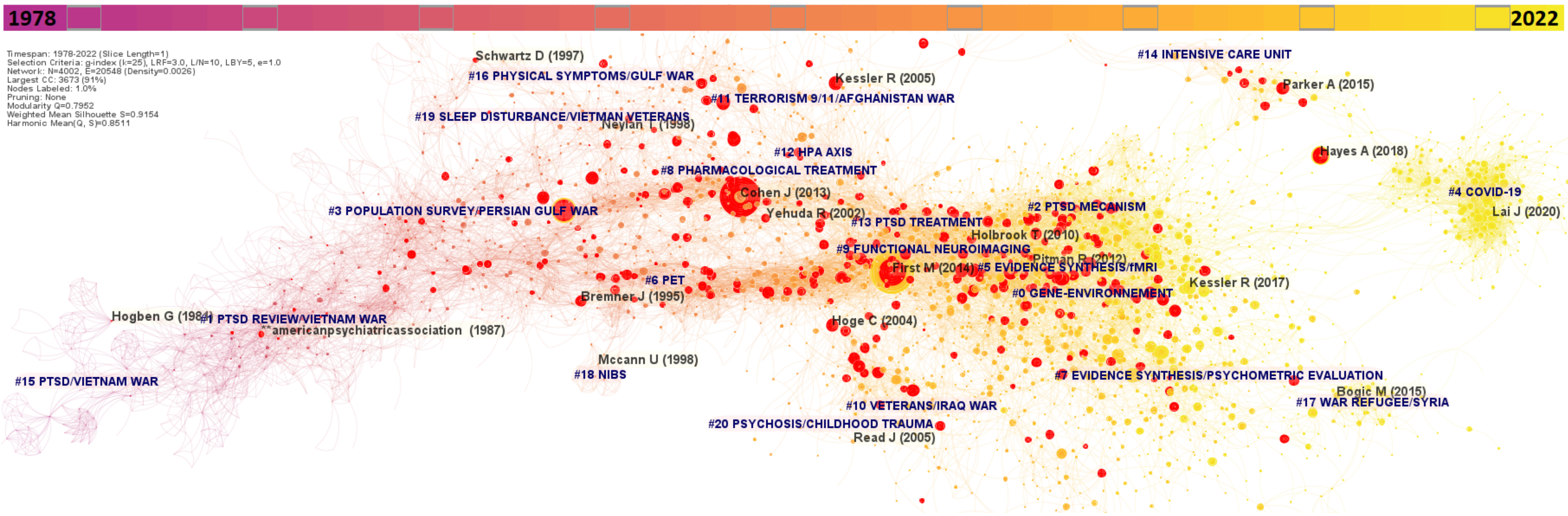


**Supplementary Figure 5**. Details of the reference co-cited network clusters (1978-2021).

**B. Cluster details by mean year for the co-cited reference network (1978-2021)**

| Cluster ID | Size | Silhouette | Mean (year) | Cluster label | Top Terms (log-likelihood ratio, p-level) |
|---|---|---|---|---|---|
| 0 | 346 | 0.842 | 2014 | EVIDENCE SYNTHESIS/GENE-ENVIRONEMENT | inflammation (273.31, 1.0E-4); cytokines (94.4, 1.0E-4); covid-19 (89.7, 1.0E-4); cardiovascular disease (84.69, 1.0E-4); gene-environment interaction (83.86, 1.0E-4) |

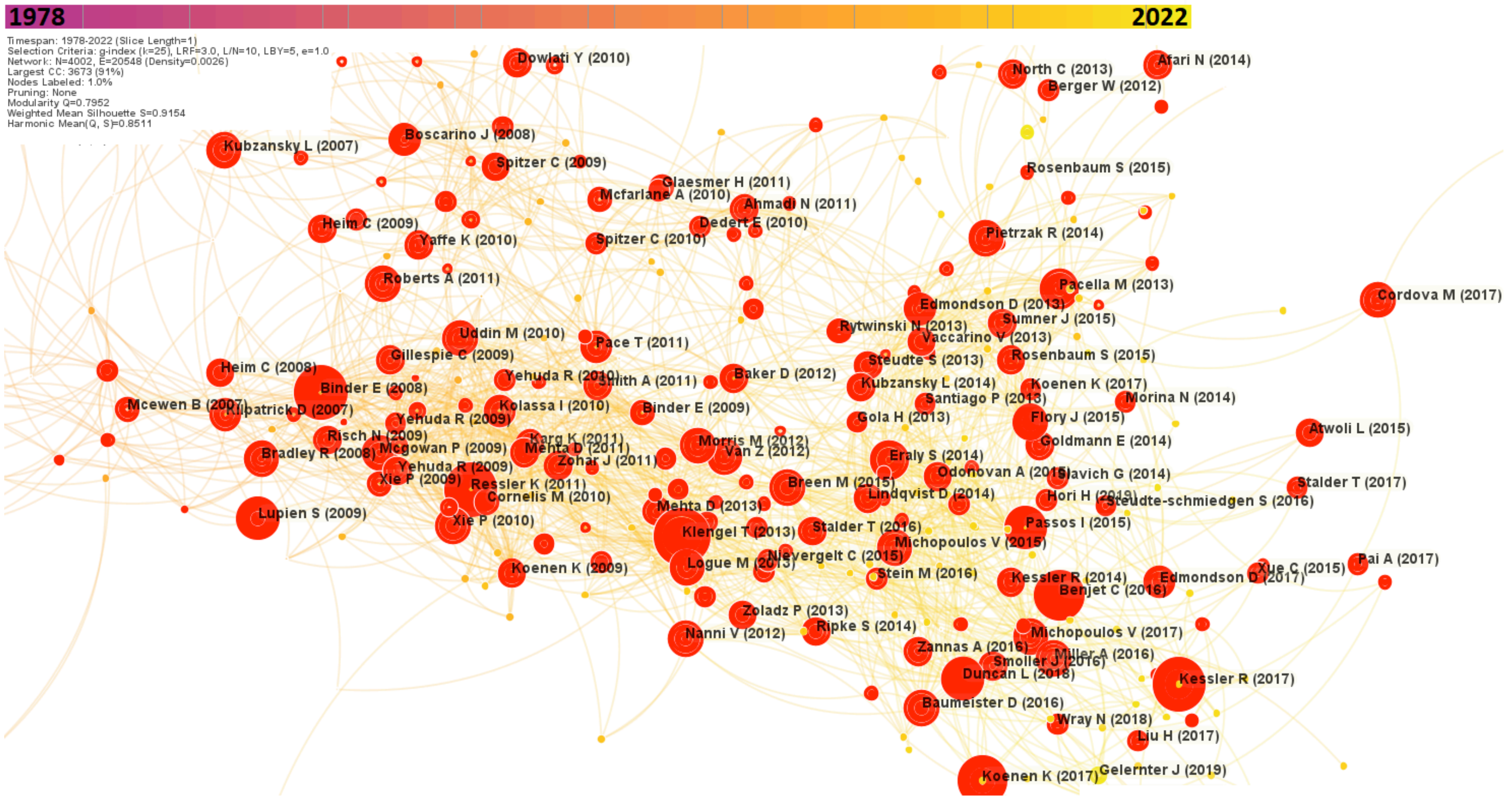


**Articles with highest centrality**

Klengel 2013 - Allele-specific FKBP5 DNA demethylation mediates gene-childhood trauma interactions

Roberts 2011- Race/ethnic differences in exposure to traumatic events, development of post-traumatic stress disorder, and treatment-seeking for post-traumatic stress disorder in the United States

Binder 2008- Association of FKBP5 polymorphisms and childhood abuse with risk of posttraumatic stress disorder symptoms in adults

Passos 2015- Inflammatory markers in post-traumatic stress disorder: a systematic review, meta-analysis, and meta-regression

Benjet 2016- The epidemiology of traumatic event exposure worldwide: results from the World Mental Health Survey Consortium

| | | | | | |
|---|---|---|---|---|---|
| 1 | 342 | 0.943 | 1989 | PTSD REVIEW/VIETNAM WAR | muscle twitch bursts (11.39, 0.001); neurohormones (11.39, 0.001); sexually abused children (11.39, 0.001); resistance veterans (11.39, 0.001); narcolepsy (11.39, 0.001) |

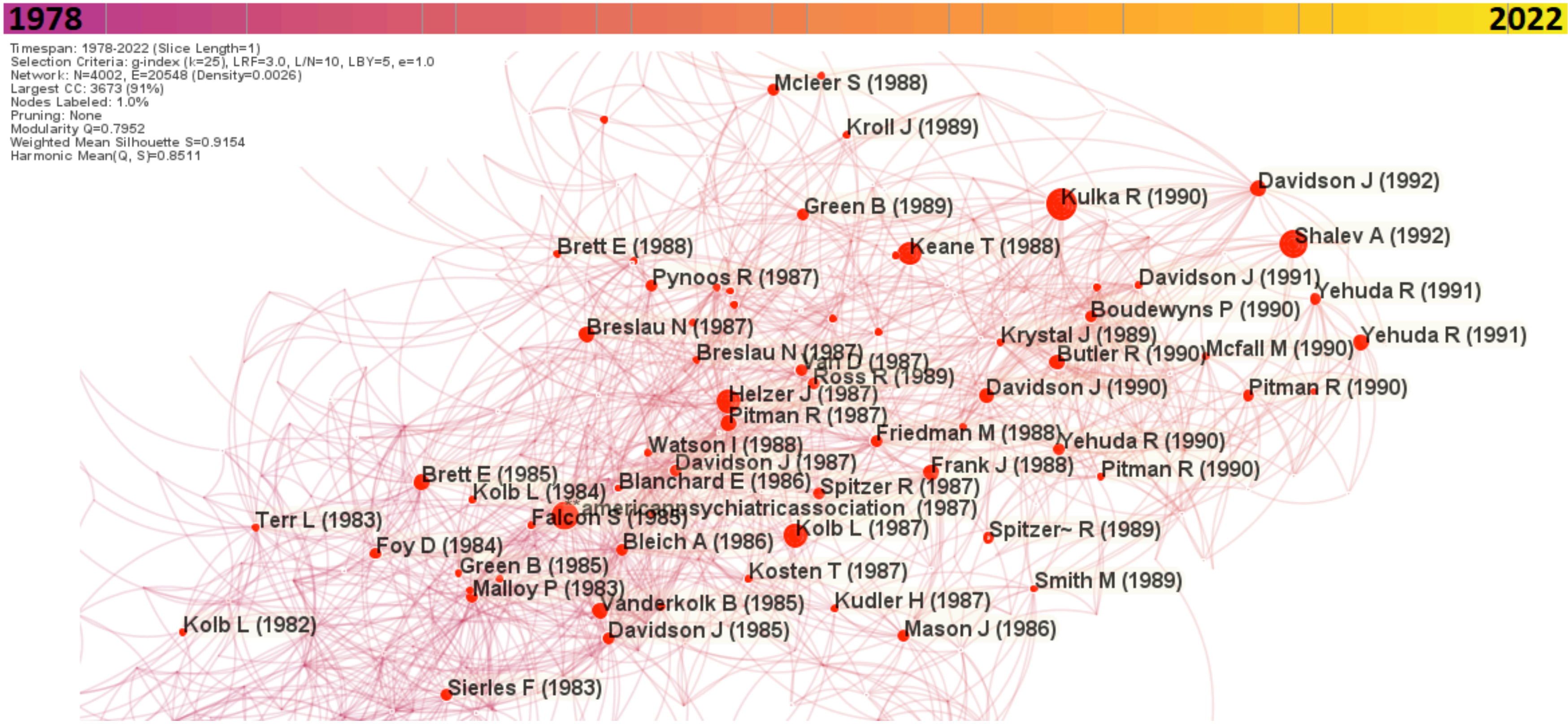


**Articles with highest centrality**

Kulka 1990- Trauma and the Vietnam war generation: Report of findings from the National Vietnam Veterans Readjustment Study

Helzer 1987- Post-traumatic stress disorder in the general population. Findings of the epidemiologic catchment area survey

Shalev 1992- Physiologic responses to loud tones in Israeli patients with posttraumatic stress disorder

Keane 1988- Mississippi Scale for Combat-Related Posttraumatic Stress Disorder: three studies in reliability and validity

Kolb 1987- A neuropsychological hypothesis explaining posttraumatic stress disorders

| | | | | | |
|---|---|---|---|---|---|
| 2 | 317 | 0.881 | 2016 | EVIDENCE SNYTHE-SIS/NEUROSCEINCE | extinction (241.86, 1.0E-4); fear conditioning (195.83, 1.0E-4); mdma (120.14, 1.0E-4); fear extinction (112.83, 1.0E-4); reconsolidation (95.5, 1.0E-4) |

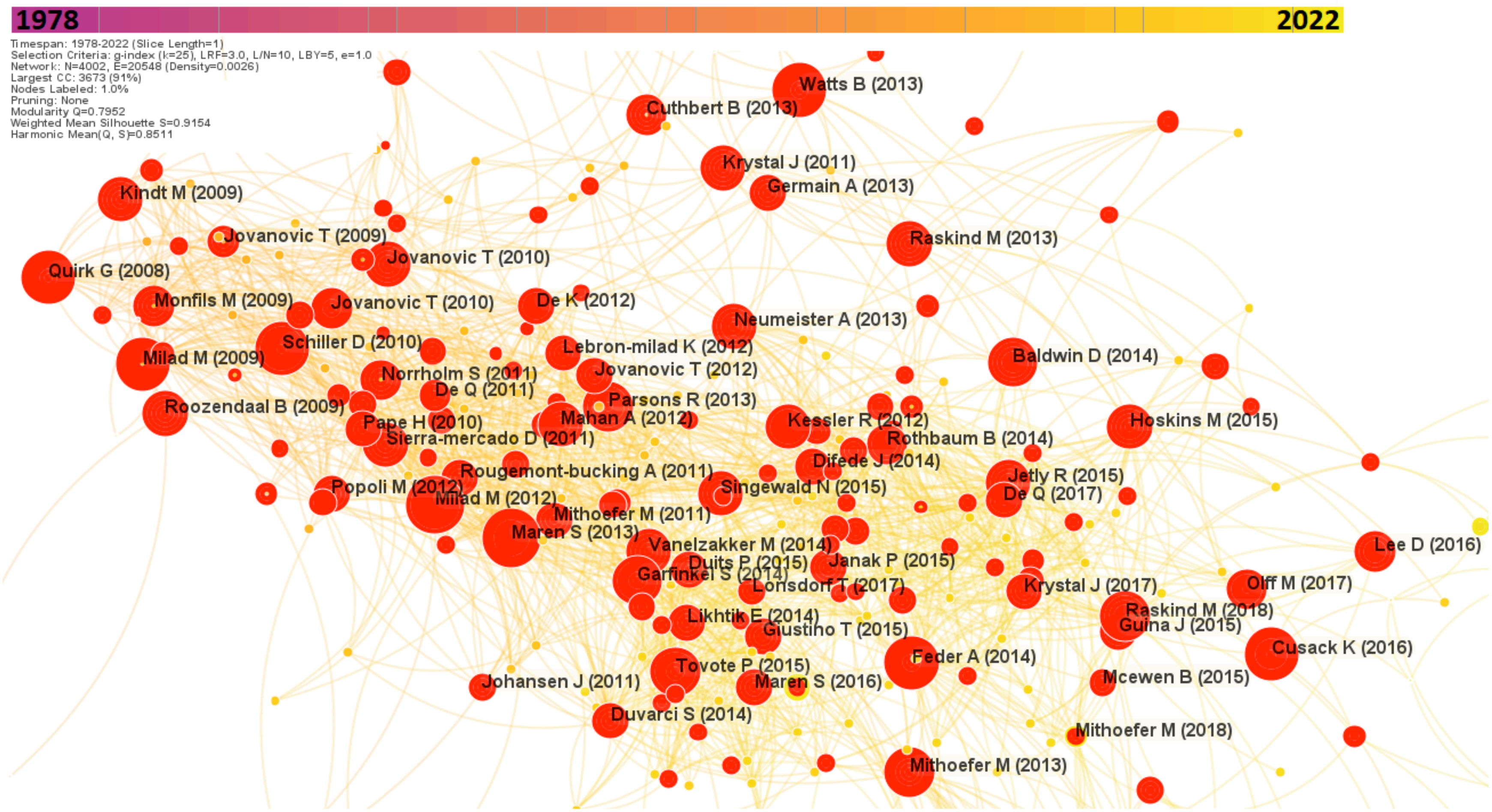


**Articles with highest centrality**

Milad 2009- Neurobiological basis of failure to recall extinction memory in posttraumatic stress disorder

Maren 2013- The contextual brain: implications for fear conditioning, extinction and psychopathology

Milad 2012- Fear Extinction as a Model for Translational Neuroscience: Ten Years of Progress

Watts 2013- Meta-analysis of the efficacy of treatments for posttraumatic stress disorder

Raskind 2018- Trial of Prazosin for Post-Traumatic Stress Disorder in Military Veterans

| | | | | | |
|---|---|---|---|---|---|
| 3 | 317 | 0.897 | 1996 | POPULATION SURVEY/ PERSIAN GULF WAR | injury (15.64, 1.0E-4); accidents (15.27, 1.0E-4); disability (14.07, 0.001); posttraumatic stress disorder (13.57, 0.001); diagnosis (13.11, 0.001) |

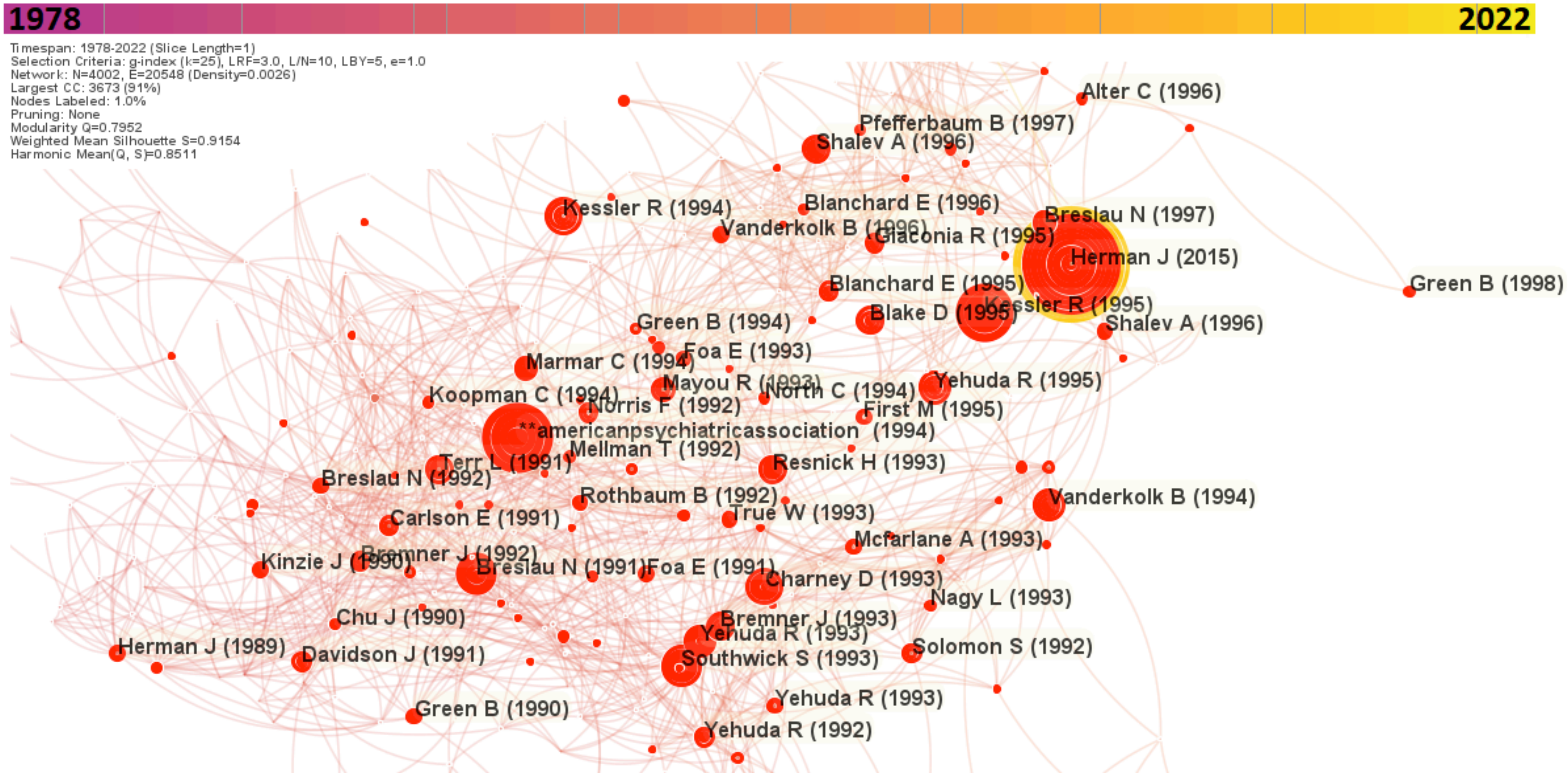


**Articles with highest centrality**

Herman 2015- Trauma and Recovery: The Aftermath of Violence--From Domestic Abuse to Political Terror

Breslau 1998- Trauma and posttraumatic stress disorder in the community: the 1996 Detroit Area Survey of Trauma

Kessler 1995- Posttraumatic stress disorder in the National Comorbidity Survey

Charney 199- Psychobiologic mechanisms of posttraumatic stress disorder

Breslau 1993- Conflict between current knowledge about posttraumatic stress disorder and its original conceptual basis

| | | | | | |
|---|---|---|---|---|---|
| 4 | 263 | 0.977 | 2020 | COVID-19 | covid-19 (1382.85, 1.0E-4); sars-cov-2 (215.97, 1.0E-4); mental health (201.3, 1.0E-4); pandemic (179.96, 1.0E-4); healthcare workers (164.88, 1.0E-4) |

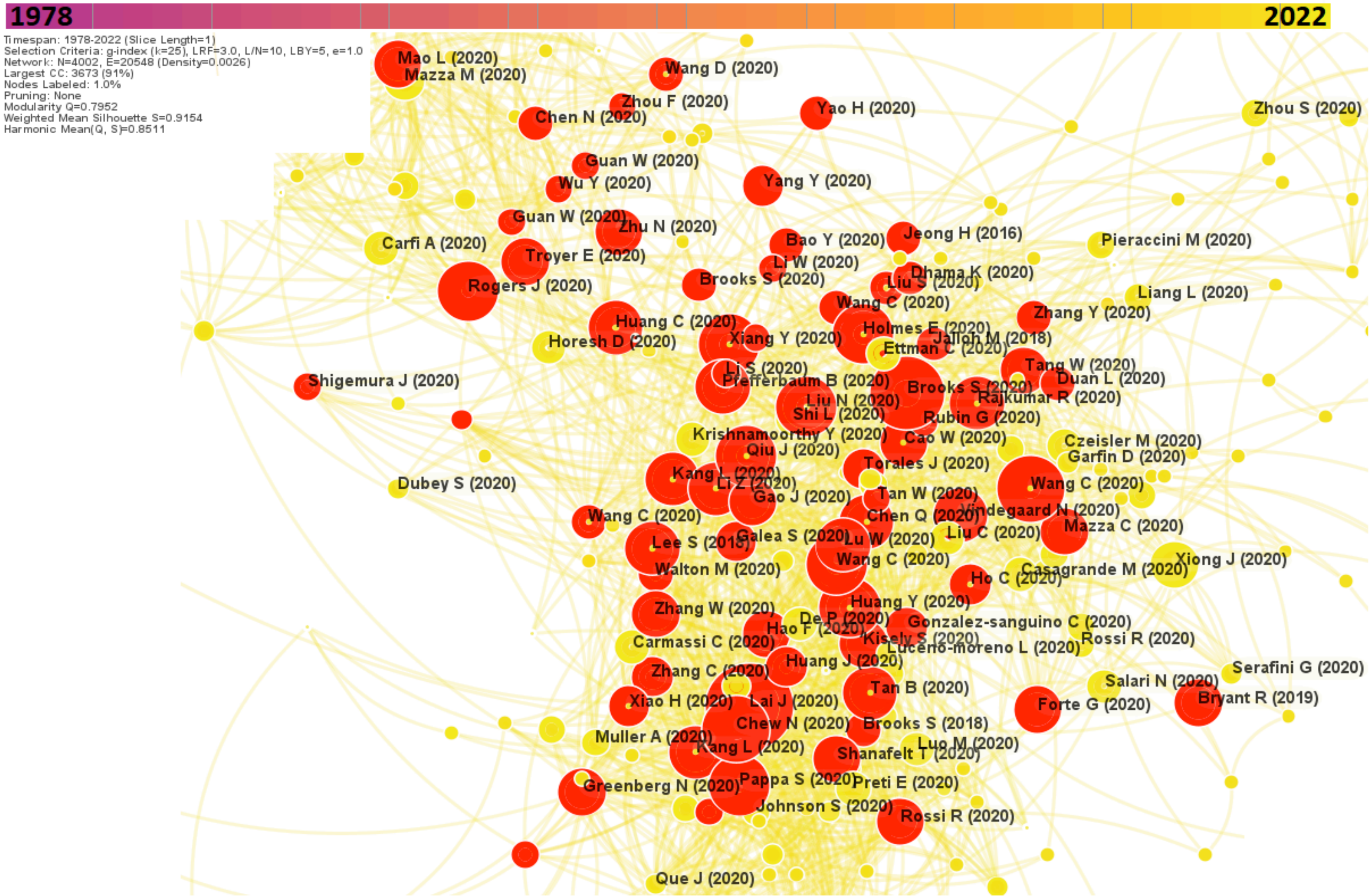


**Articles with highest centrality**

Boyaz 2020- Coronavirus Disease (COVID-19) and Traumatic Stress: Probable Risk Factors and Correlates of Posttraumatic Stress Disorder

Brooks 2020- The psychological impact of quarantine and how to reduce it: rapid review of the evidence

Holmes 2020- Multidisciplinary research priorities for the COVID-19 pandemic: a call for action for mental health science

Wang 2020- Does comorbidity increase the risk of patients with COVID-19: evidence from meta-analysis

Pappa 2020- Prevalence of depression, anxiety, and insomnia among healthcare workers during the COVID-19 pandemic: A systematic review and meta-analysis

| | | | | | |
|---|---|---|---|---|---|
| 5 | 252 | 0.863 | 2015 | EVIDENCE SYNTHESIS/fMRI | fmri (144.31, 1.0E-4); functional connectivity (128.62, 1.0E-4); default mode network (79.77, 1.0E-4); amygdala (74.41, 1.0E-4); covid-19 (73.04, 1.0E-4) |

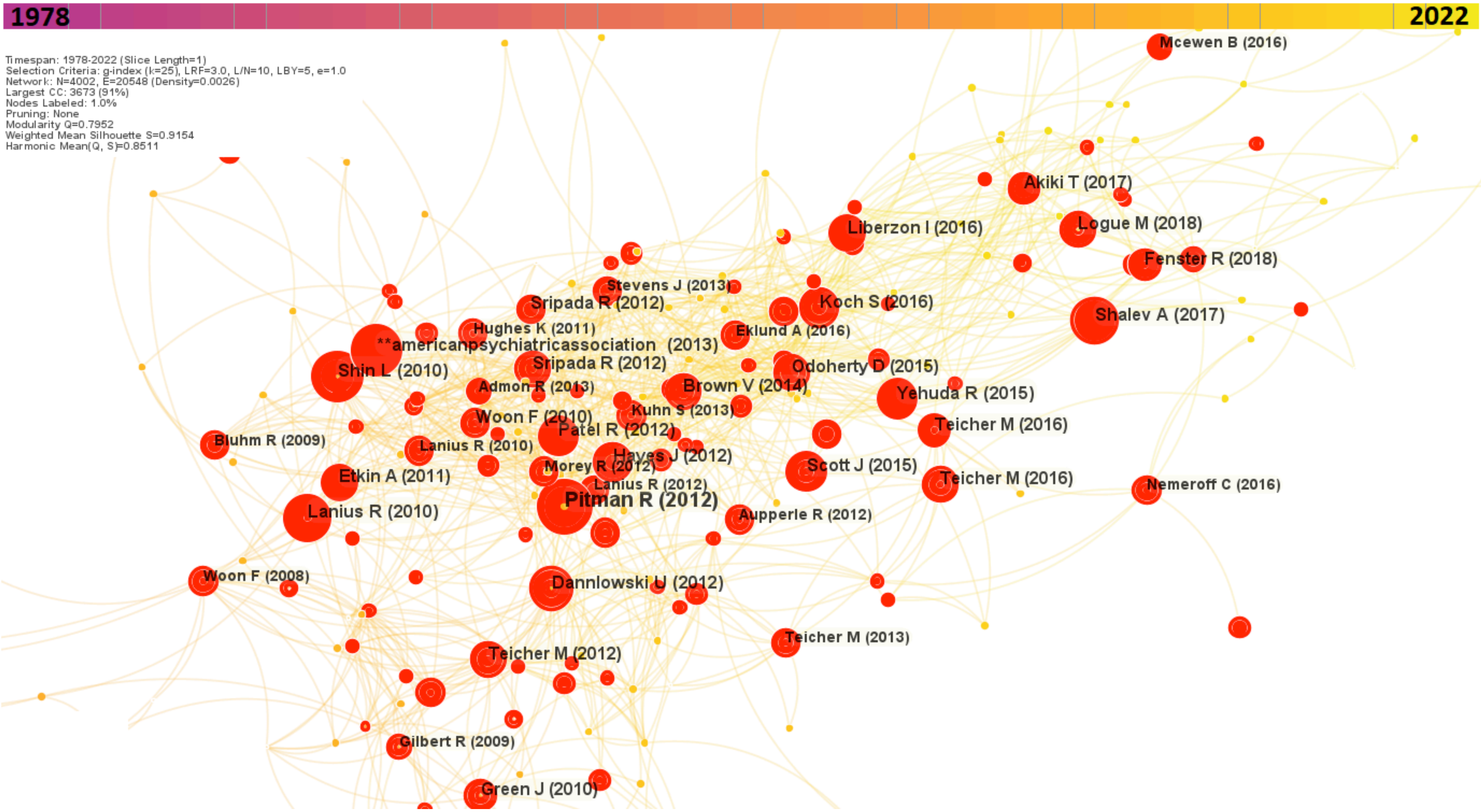


**Articles with highest centrality**

Patel 2012- Neurocircuitry models of posttraumatic stress disorder and beyond: a meta-analysis of functional neuroimaging studies

Pitman 2012- Biological studies of post-traumatic stress disorder

Shalev 2017- Post-Traumatic Stress Disorder

Shin 2010- The Neurocircuitry of Fear, Stress, and Anxiety Disorders

Lanius 2010- Emotion modulation in PTSD: Clinical and neurobiological evidence for a dissociative subtype

| | | | | | |
|---|---|---|---|---|---|
| 6 | 241 | 0.914 | 2000 | AFFECTIVE NEUROSCI-ENCE/MRI/PET | positron emission tomography (34.6, 1.0E-4); limbic system (33.84, 1.0E-4); crf (27.93, 1.0E-4); cortisol (22.61, 1.0E-4); child maltreatment (22.54, 1.0E-4) |

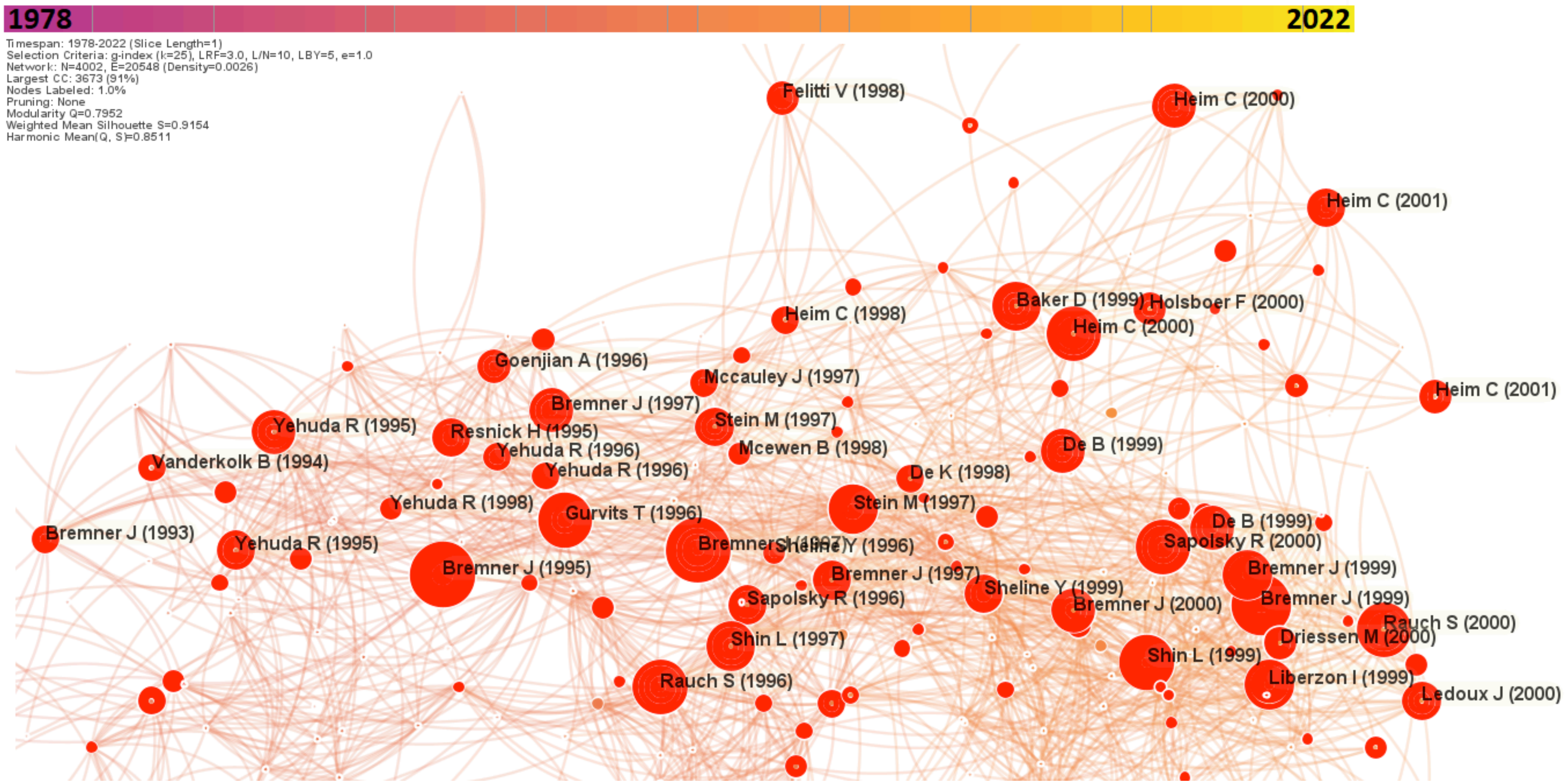


**Articles with highest centrality**

Bremmer 1995- MRI-based measurement of hippocampal volume in patients with combat-related posttraumatic stress disorder

Bremmer 1997- Magnetic resonance imaging-based measurement of hippocampal volume in posttraumatic stress disorder related to childhood physical and sexual abuse--a preliminary report

Bremmer 1999- Does stress damage the brain?

Rauch 2000- Exaggerated amygdala response to masked facial stimuli in posttraumatic stress disorder: a functional MRI study

Shin 1999- Regional cerebral blood flow during script-driven imagery in childhood sexual abuse-related PTSD: A PET investigation

| | | | | | |
|---|---|---|---|---|---|
| 7 | 240 | 0.937 | 2017 | EVIDENCE SYNTHESIS/ PSYCHOMETRIC EVALUATION | icd-11 (165.58, 1.0E-4); dsm-5 (142.94, 1.0E-4); network analysis (136.87, 1.0E-4); veterans (95.01, 1.0E-4); postpartum (90.55, 1.0E-4) |

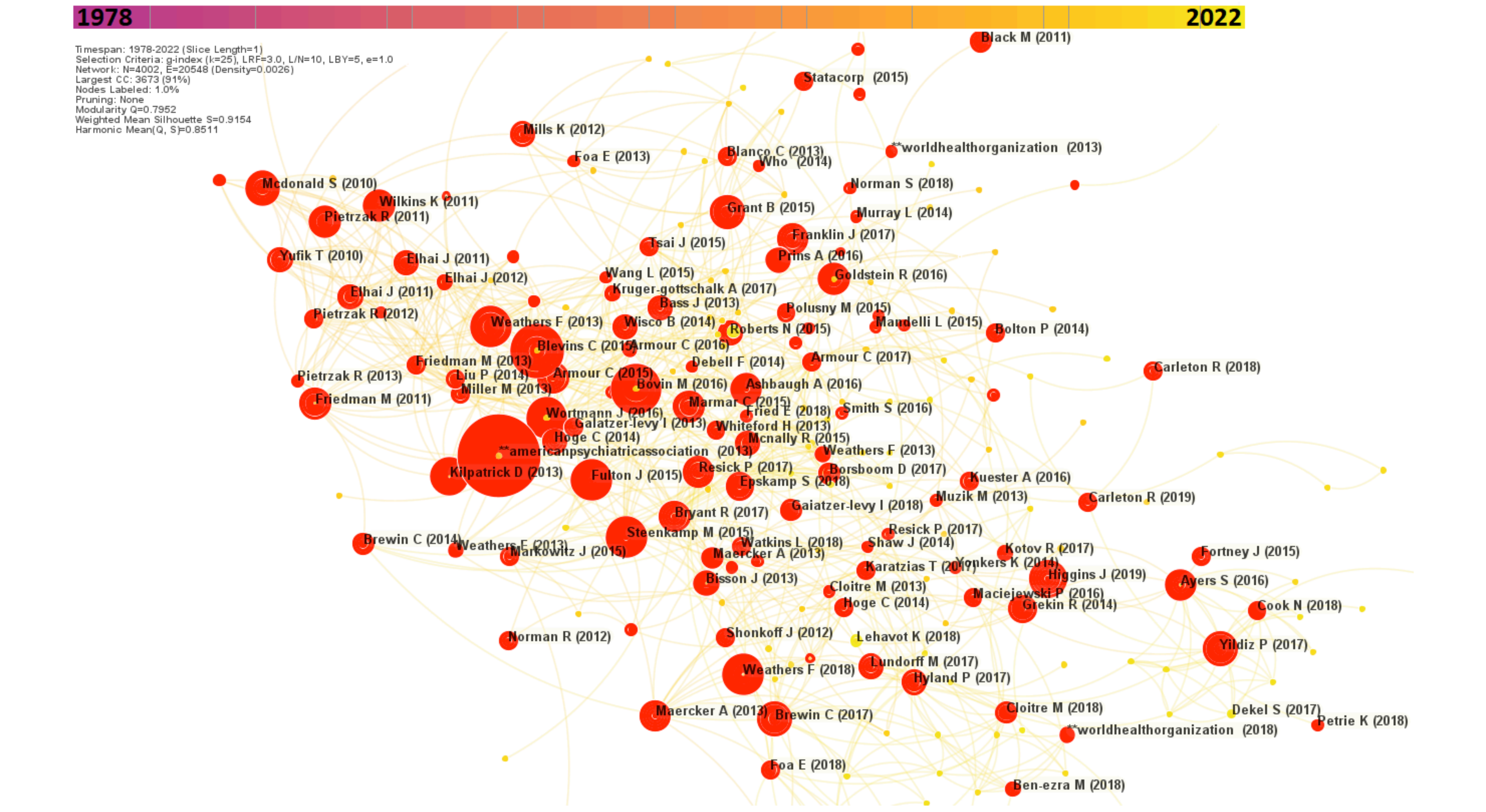


**Articles with highest centrality**

Blevins 2015- The Posttraumatic Stress Disorder Checklist for DSM-5 (PCL-5): Development and Initial Psychometric Evaluation

Bovin 2016- Psychometric Properties of the PTSD Checklist for Diagnostic and Statistical Manual of Mental Disorders–Fifth Edition (PCL-5) in Veterans

Weathers 2013- PTSD Checklist for DSM-5

Wortmann 2016- Psychometric Analysis of the PTSD Checklist-5 (PCL-5) Among Treatment-Seeking Military Service Members

Weathers 2018- The Clinician-Administered PTSD Scale for DSM-5 (CAPS-5): Development and initial psychometric evaluation in military veterans

| | | | | | |
|---|---|---|---|---|---|
| 8 | 233 | 0.897 | 2002 | PHARMACOLOGICAL TREATMENT | pharmacotherapy (36.89, 1.0E-4); fluoxetine (32.28, 1.0E-4); olanzapine (32.28, 1.0E-4); mirtazapine (32.28, 1.0E-4); paroxetine (29.91, 1.0E-4) |

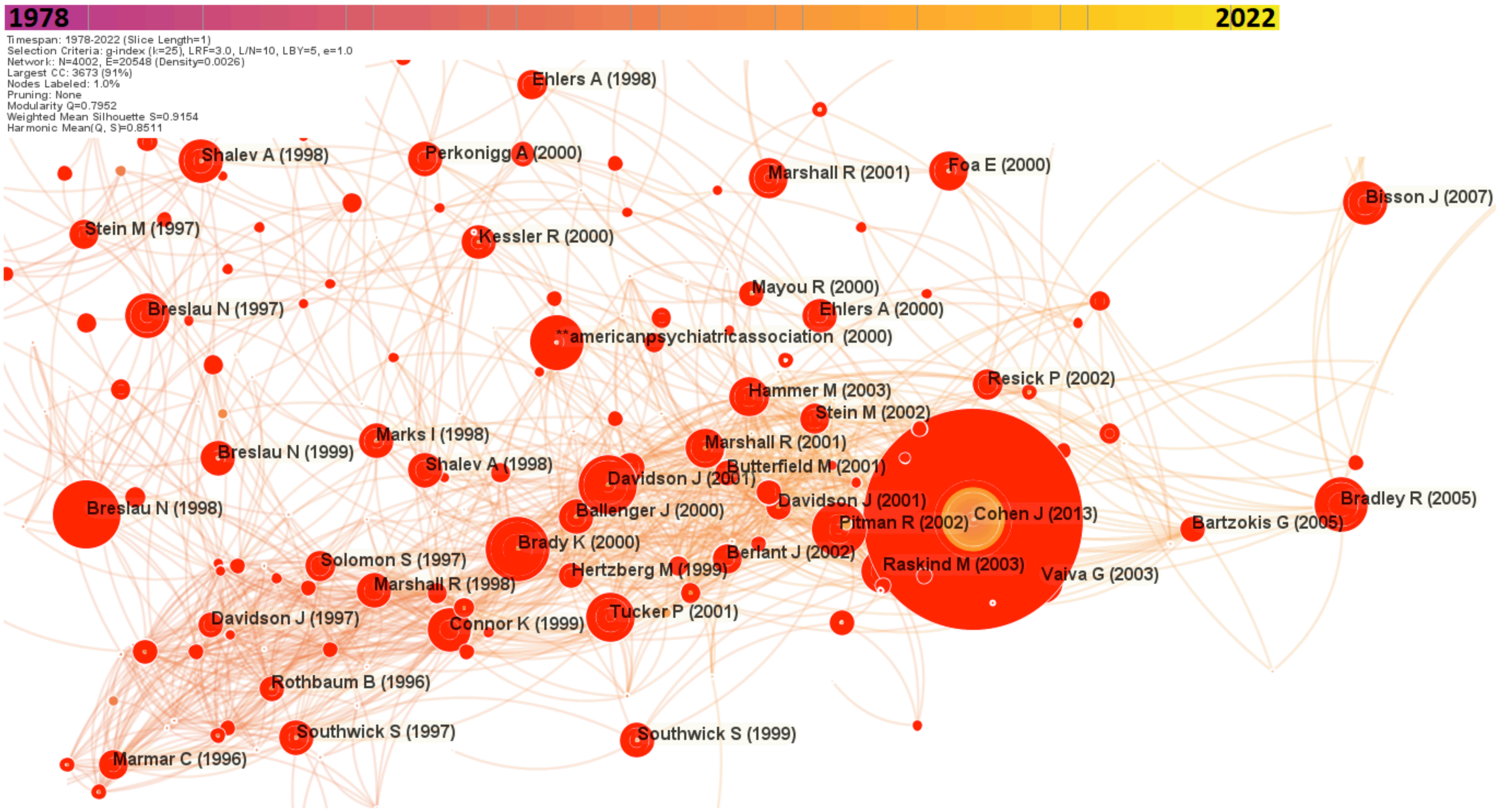


**Articles with highest centrality**

Brady 2000- Efficacy and safety of sertraline treatment of posttraumatic stress disorder: a randomized controlled trial

Cohen 2013- Statistical Power Analysis for the Behavioral Sciences

Breslau 1998- Trauma and posttraumatic stress disorder in the community: the 1996 Detroit Area Survey of Trauma

Davidson 2001- Multicenter, double-blind comparison of sertraline and placebo in the treatment of posttraumatic stress disorder

Pitman 2002- Pilot study of secondary prevention of posttraumatic stress disorder with propranolol

| | | | | | |
|---|---|---|---|---|---|
| 9 | 220 | 0.908 | 2007 | FUNCTIONAL NEUROIMAGING | amygdala (105.09, 1.0E-4); hippocampus (77.17, 1.0E-4); fmri (71.9, 1.0E-4); magnetic resonance imaging (62.6, 1.0E-4); neuroimaging (40.35, 1.0E-4) |

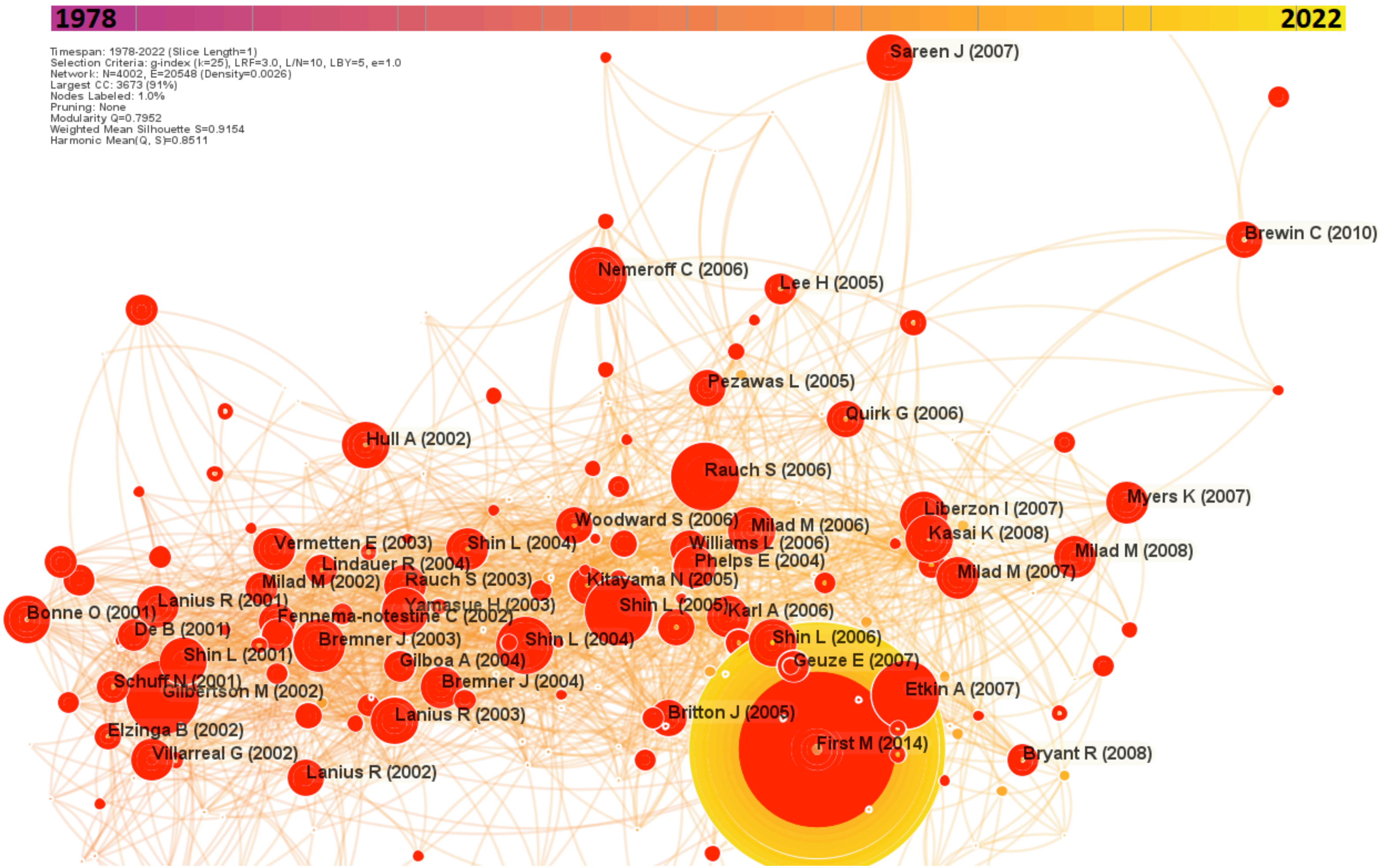


**Articles with highest centrality**

Gilbertson 2002- Smaller hippocampal volume predicts pathologic vulnerability to psychological trauma

Etkin 2007- Functional neuroimaging of anxiety: a meta-analysis of emotional processing in PTSD, social anxiety disorder, and specific phobia

Shin 2005- A Functional Magnetic Resonance Imaging Study of Amygdala and Medial Prefrontal Cortex Responses to Overtly Presented Fearful Faces in Posttraumatic Stress Disorder

Rauch 2006- Neurocircuitry models of posttraumatic stress disorder and extinction: human neuroimaging research--past, present, and future

Kasai 2008- Evidence for Acquired Pregenual Anterior Cingulate Gray Matter Loss from A Twin Study of Combat-Related Post-Traumatic Stress Disorder

| | | | | | |
|---|---|---|---|---|---|
| 10 | 194 | 0.944 | 2011 | VETERANS/IRAQ WAR | military personnel (139.48, 1.0E-4); veterans (99.75, 1.0E-4); military (87.34, 1.0E-4); deployment (66.37, 1.0E-4); combat (49.65, 1.0E-4) |

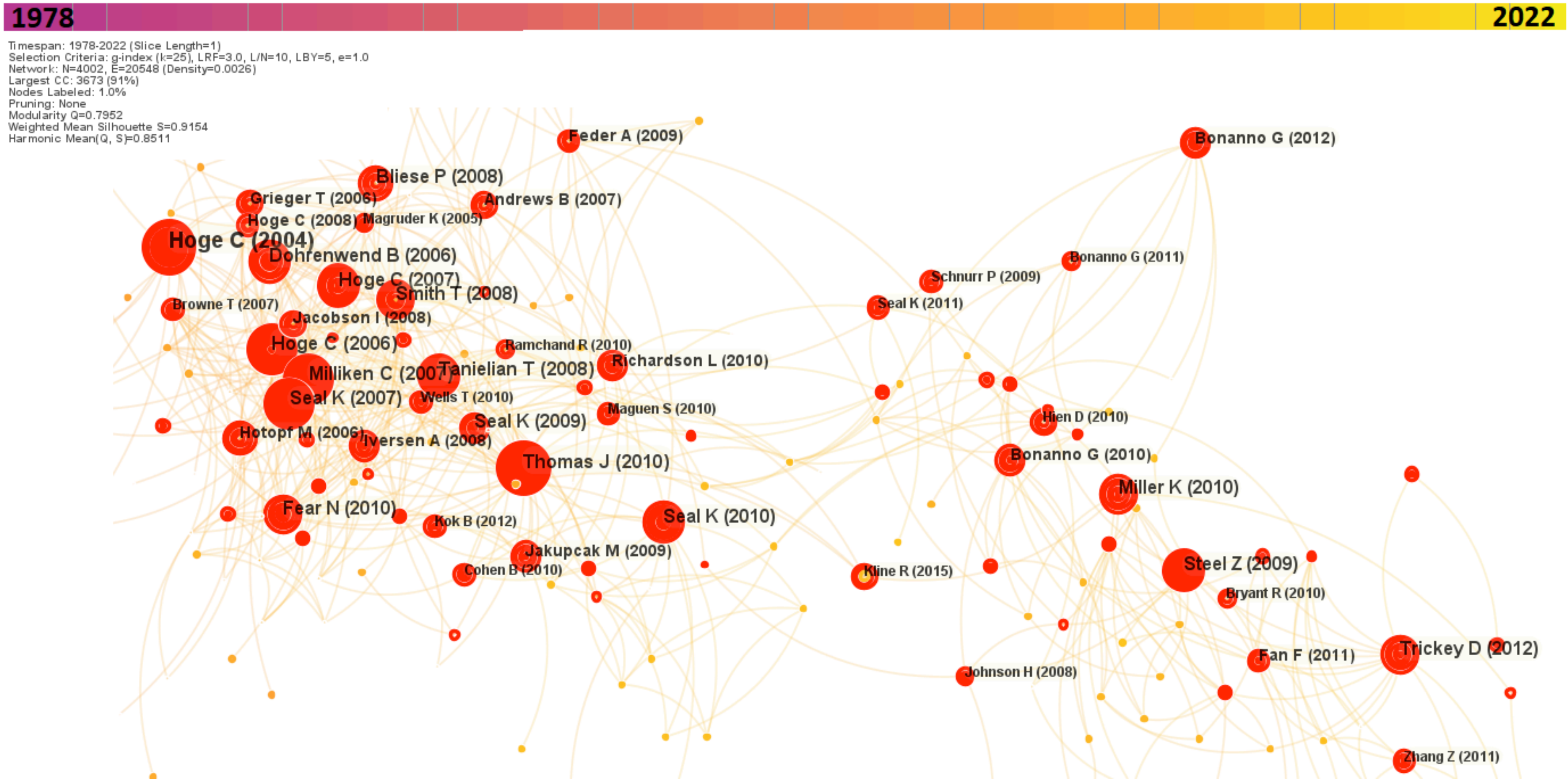


**Articles with highest centrality**

Thomas 2010- Prevalence of mental health problems and functional impairment among active component and National Guard soldiers 3 and 12 months following combat in Iraq

Hoge 2004- Combat duty in Iraq and Afghanistan, mental health problems, and barriers to care

Steel 2009 - Association of torture and other potentially traumatic events with mental health outcomes among populations exposed to mass conflict and displacement: a systematic review and meta-analysis

Miller 2010- War exposure, daily stressors, and mental health in conflict and post-conflict settings: bridging the divide between trauma-focused and psychosocial frameworks

Tanielian 2008- Invisible Wounds of War. Psychological and Cognitive Injuries, Their Consequences, and Services to Assist Recovery

| | | | | | |
|---|---|---|---|---|---|
| 11 | 164 | 0.92 | 2007 | TERRORISM 9/11/ AFGHANI-STAN WAR | terrorism (114.47, 1.0E-4); disaster (97.97, 1.0E-4); earthquake (67.66, 1.0E-4); disasters (55.92, 1.0E-4); nicotine dependence (32.92, 1.0E-4) |

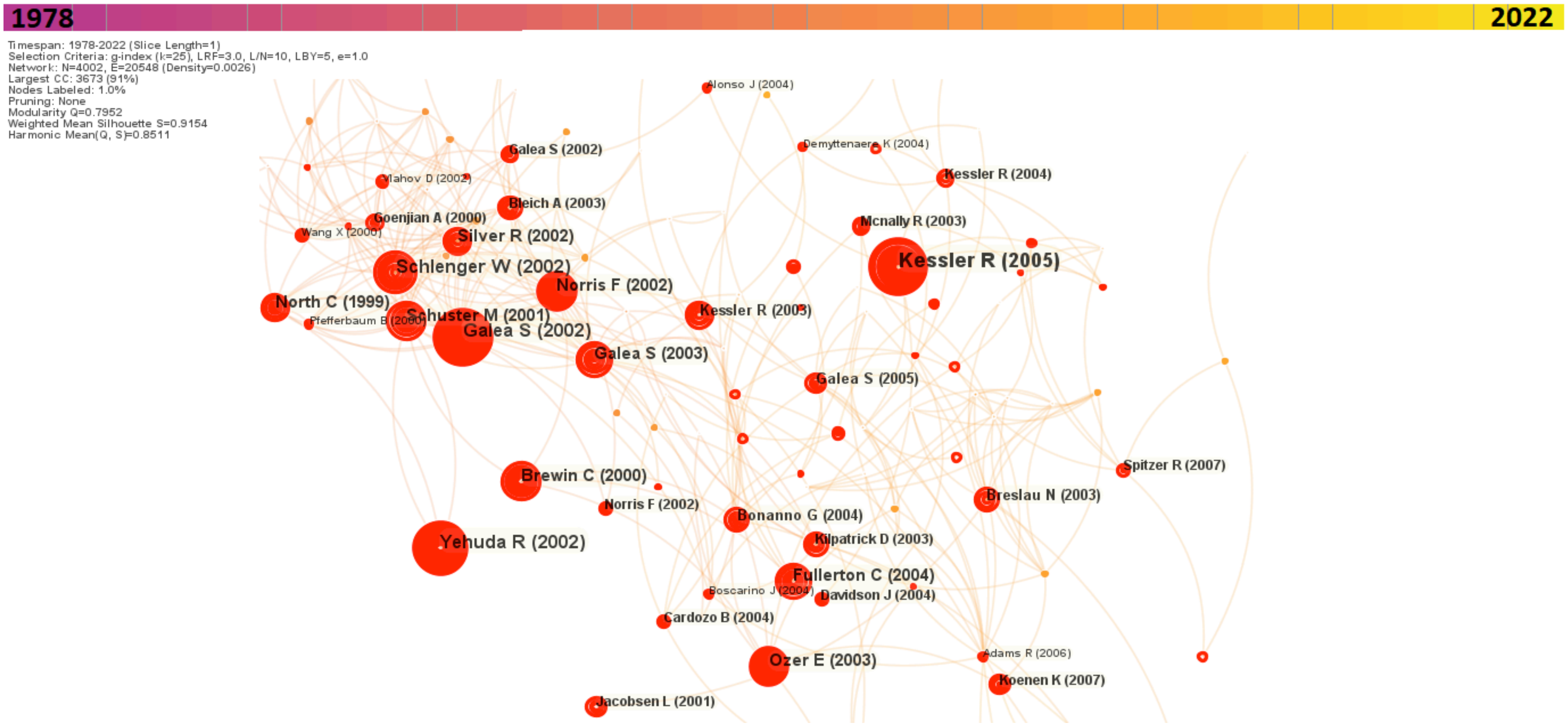


**Articles with highest centrality**

Kessler 2005- Lifetime prevalence and age-of-onset distributions of DSM-IV disorders in the National Comorbidity Survey Replication

Galea 2002- Psychological Sequelae of the September 11 Terrorist Attacks in New York City

Yehuda 2002- Post-traumatic stress disorder

Schlenger 2002- Psychological Reactions to Terrorist Attacks Findings From the National Study of Americans' Reactions to September 11

Norris 2002- 60,000 disaster victims speak: Part I. An empirical review of the empirical literature, 1981-200

| | | | | | |
|---|---|---|---|---|---|
| 12 | 119 | 0.932 | 2006 | HPA AXIS | cortisol (163.49, 1.0E-4); hpa axis (115.26, 1.0E-4); salivary cortisol (49.77, 1.0E-4); fibromyalgia (31.17, 1.0E-4); acth (29.37, 1.0E-4) |

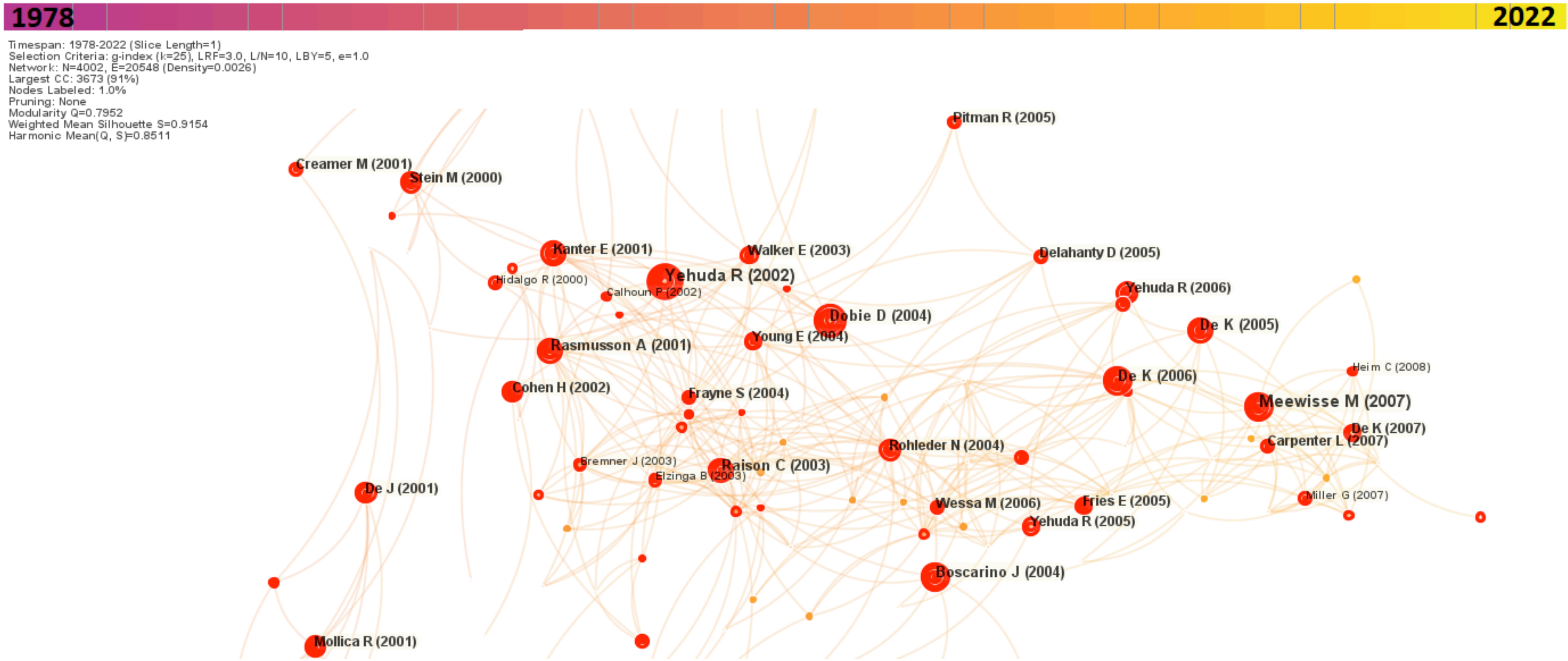


**Articles with highest centrality**

Meewisse 2007- Cortisol and post-traumatic stress disorder in adults: systematic review and meta-analysis

Yehuda 2002- Current status of cortisol findings in post-traumatic stress disorder

Boscarino 2004- Posttraumatic stress disorder and physical illness: results from clinical and epidemiologic studies

Dobie 2004- Posttraumatic stress disorder in female veterans: association with self-reported health problems and functional impairment

Knater 2001- Glucocorticoid feedback sensitivity and adrenocortical responsiveness in posttraumatic stress disorder

| | | | | | |
|---|---|---|---|---|---|
| 13 | 100 | 0.919 | 2009 | PTSD TREATMENT | prazosin (27.32, 1.0E-4); covid-19 (26.63, 1.0E-4); mental health (24.34, 1.0E-4); memory (22.78, 1.0E-4); cognitive behavioral therapy (20.7, 1.0E-4) |

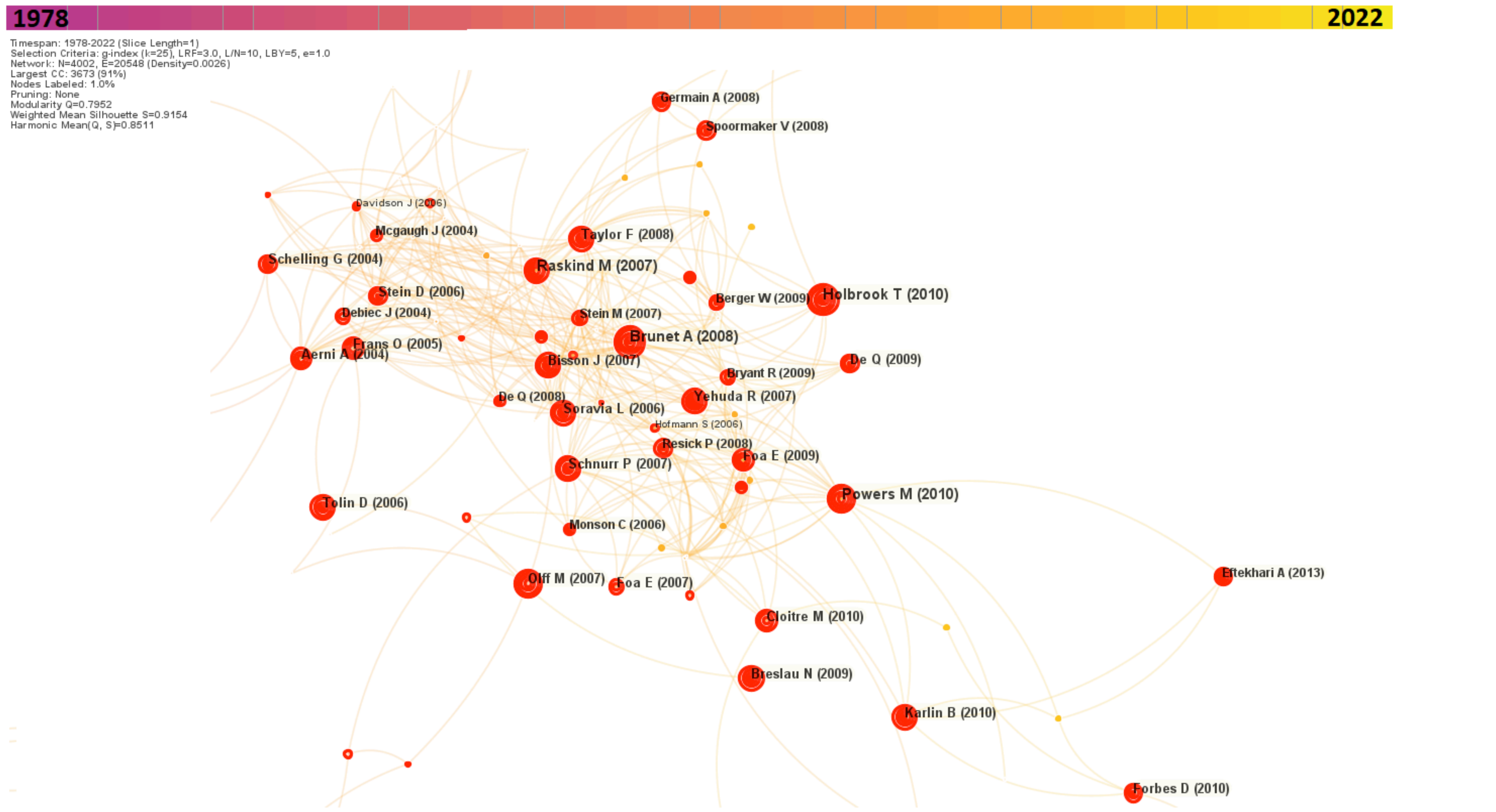


**Articles with highest centrality**

Raskinf 2007- A parallel group placebo controlled study of prazosin for trauma nightmares and sleep disturbance in combat veterans with post-traumatic stress disorder

Holbrook 2010- Morphine Use after Combat Injury in Iraq and Post-Traumatic Stress Disorder

Powers 2010- A meta-analytic review of prolonged exposure for posttraumatic stress disorder

Brunet 2008- Effect of post-retrieval propranolol on psychophysiologic responding during subsequent script-driven traumatic imagery in post-traumatic stress disorder

Schnurr 2007- Cognitive behavioral therapy for posttraumatic stress disorder in women: a randomized controlled trial

| | | | | | |
|---|---|---|---|---|---|
| 14 | 85 | 0.971 | 2012 | INTENSIVE CARE UNIT | critical illness (238.75, 1.0E-4); critical care (231.76, 1.0E-4); intensive care (223.14, 1.0E-4); delirium (167.45, 1.0E-4); intensive care unit (134.28, 1.0E-4) |

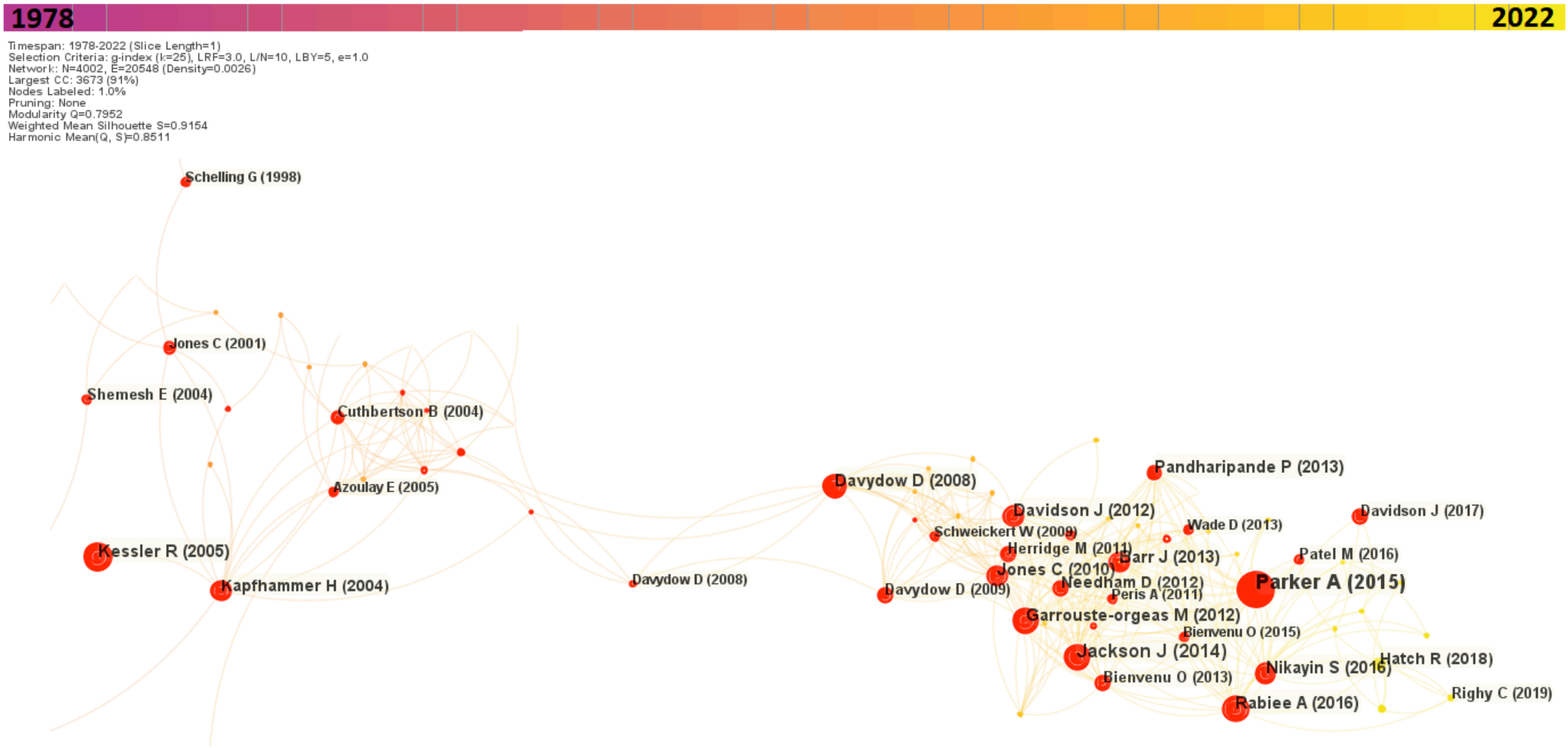


**Articles with highest centrality**

Davydow 2008- Posttraumatic stress disorder in general intensive care unit survivors: a systematic review

Parker 2015- Posttraumatic stress disorder in critical illness survivors: a metaanalysis

Kessler 2005 - Lifetime prevalence and age-of-onset distributions of DSM-IV disorders in the National Comorbidity Survey Replication

Garrouste-orgeas- Impact of an intensive care unit diary on psychological distress in patients and relatives

Kapfhammer 2004- Posttraumatic stress disorder and health-related quality of life in long-term survivors of acute respiratory distress syndrome

| | | | | | |
|---|---|---|---|---|---|
| 15 | 80 | 0.993 | 1982 | PTSD/VIETNAM WAR | general practitioner (NaN, 1.0); total brain volume (NaN, 1.0); animal models (NaN, 1.0); cortical networks (NaN, 1.0); prolonged exposure therapy (NaN, 1.0) |

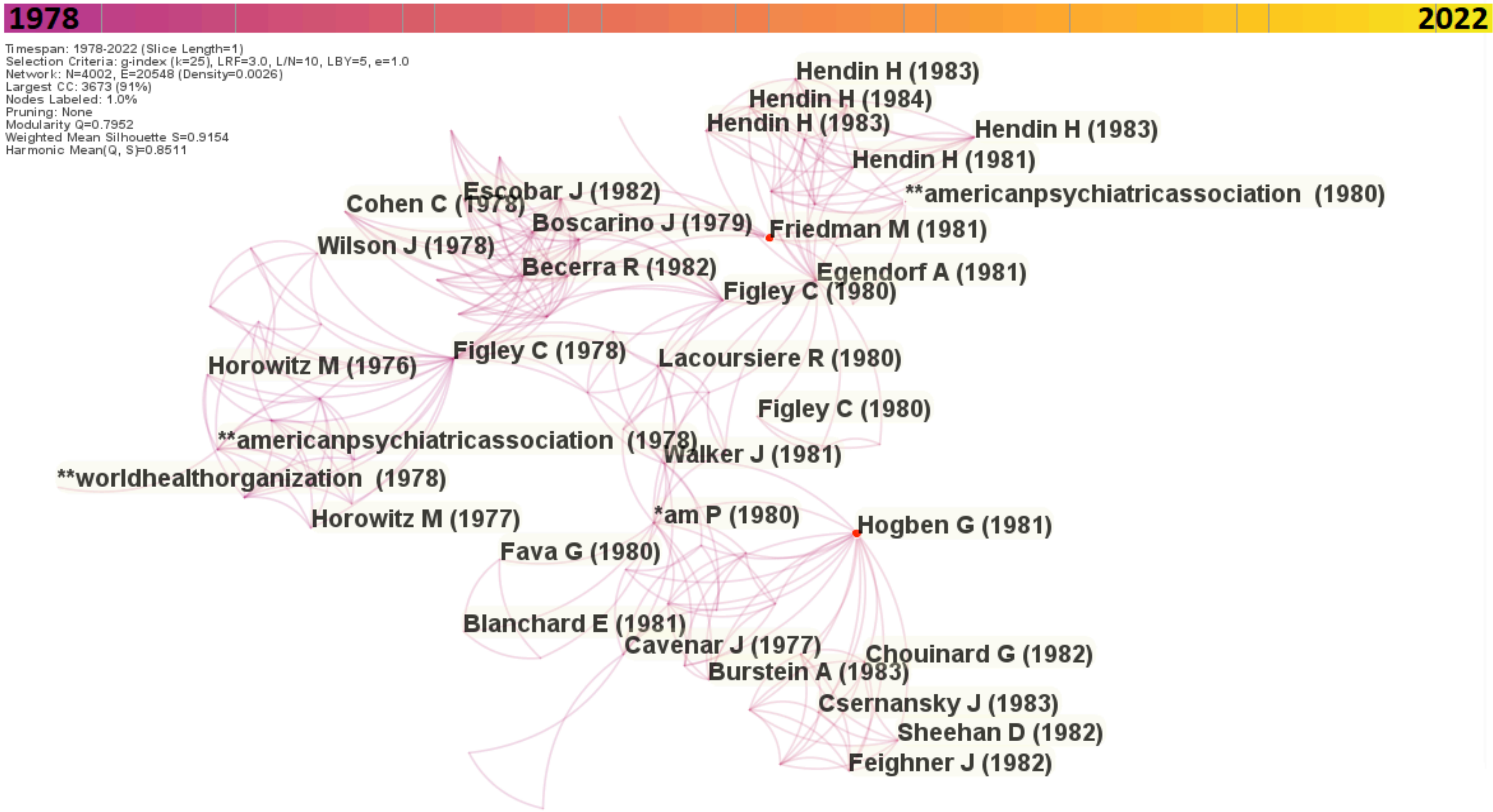


**Articles with highest centrality**

Friedman 1981- Post-Vietnam syndrome: recognition and management

Egendorf 1981- Legacies of Vietnam : comparative adjustment of veterans and their peers : a study

Hendin 1983- Psychotherapy for Vietnam veterans with posttraumatic stress disorders

Hendin 1981- Meanings of combat and the development of posttraumatic stress disorder

Hogben 1981- Treatment of traumatic war neurosis with phenelzine

| | | | | | |
|---|---|---|---|---|---|
| 16 | 66 | 0.974 | 1999 | PHYSICAL SYMPTOMS/GULF WAR | multiple chemical sensitivity (57.16, 1.0E-4); persian gulf syndrome (38.07, 1.0E-4); gulf war syndrome (30.46, 1.0E-4); mcs (28.53, 1.0E-4); health symptoms (24.05, 1.0E-4) |

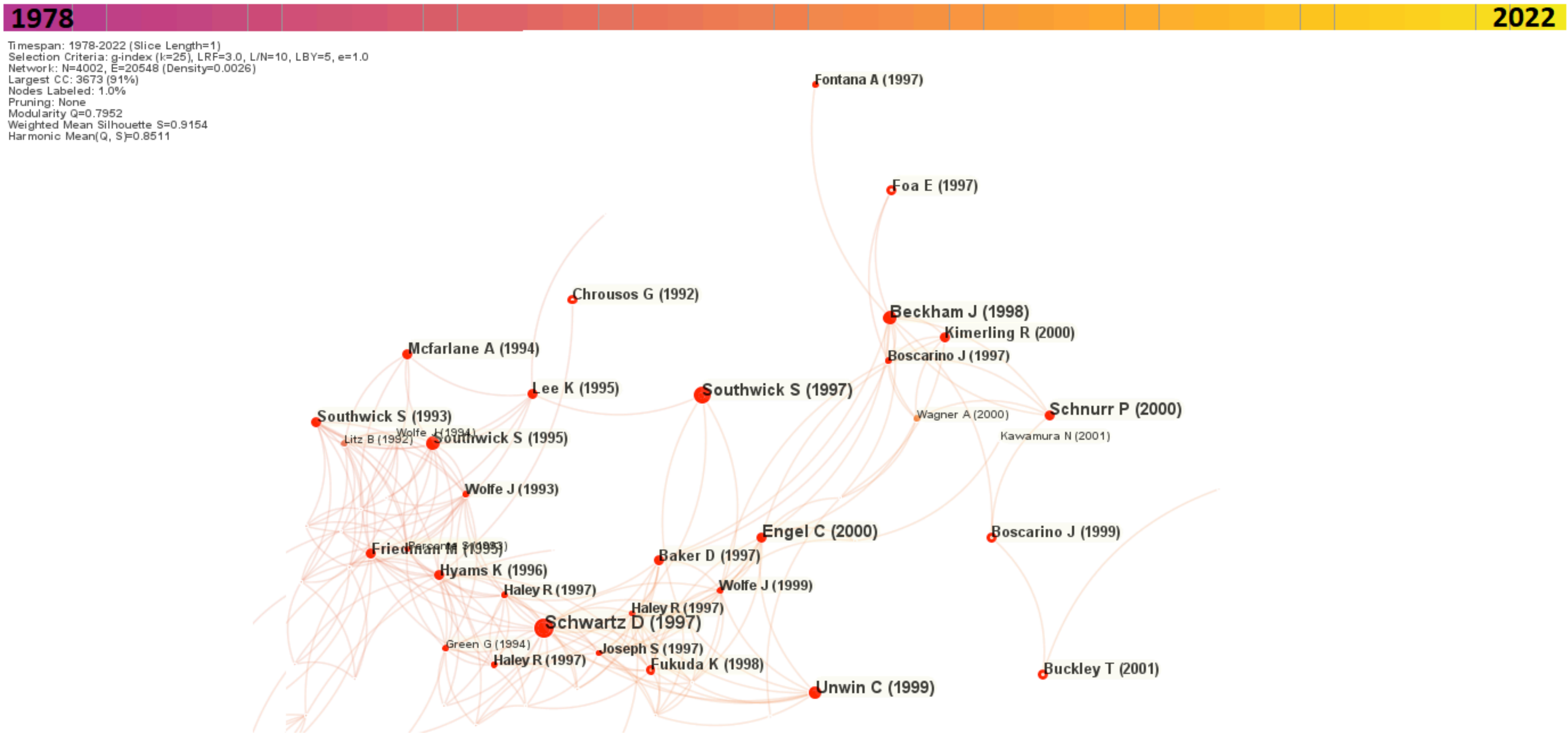


**Articles with highest centrality**

Schwartz 1997- Self-reported illness and health status among Gulf War veterans: A population-based study

Engel 2000- Relationship of Physical Symptoms to Posttraumatic Stress Disorder Among Veterans Seeking Care for Gulf War-Related Health Concerns

Southwick 1997- Consistency of memory for combat-related traumatic events in veterans of Operation Desert Storm

Beckham 1998- Health status, somatization, and severity of posttraumatic stress disorder in Vietnam combat veterans with posttraumatic stress disorder

Schnuur 2000- Physician-diagnosed medical disorders in relation to PTSD symptoms in older male military veterans

| | | | | | |
|---|---|---|---|---|---|
| 17 | 57 | 0.992 | 2019 | WAR REFUGEE/SYRIA | refugees (187.4, 1.0E-4); refugee (118.33, 1.0E-4); asylum seekers (88.44, 1.0E-4); syria (69.58, 1.0E-4); mental health (64.76, 1.0E-4) |

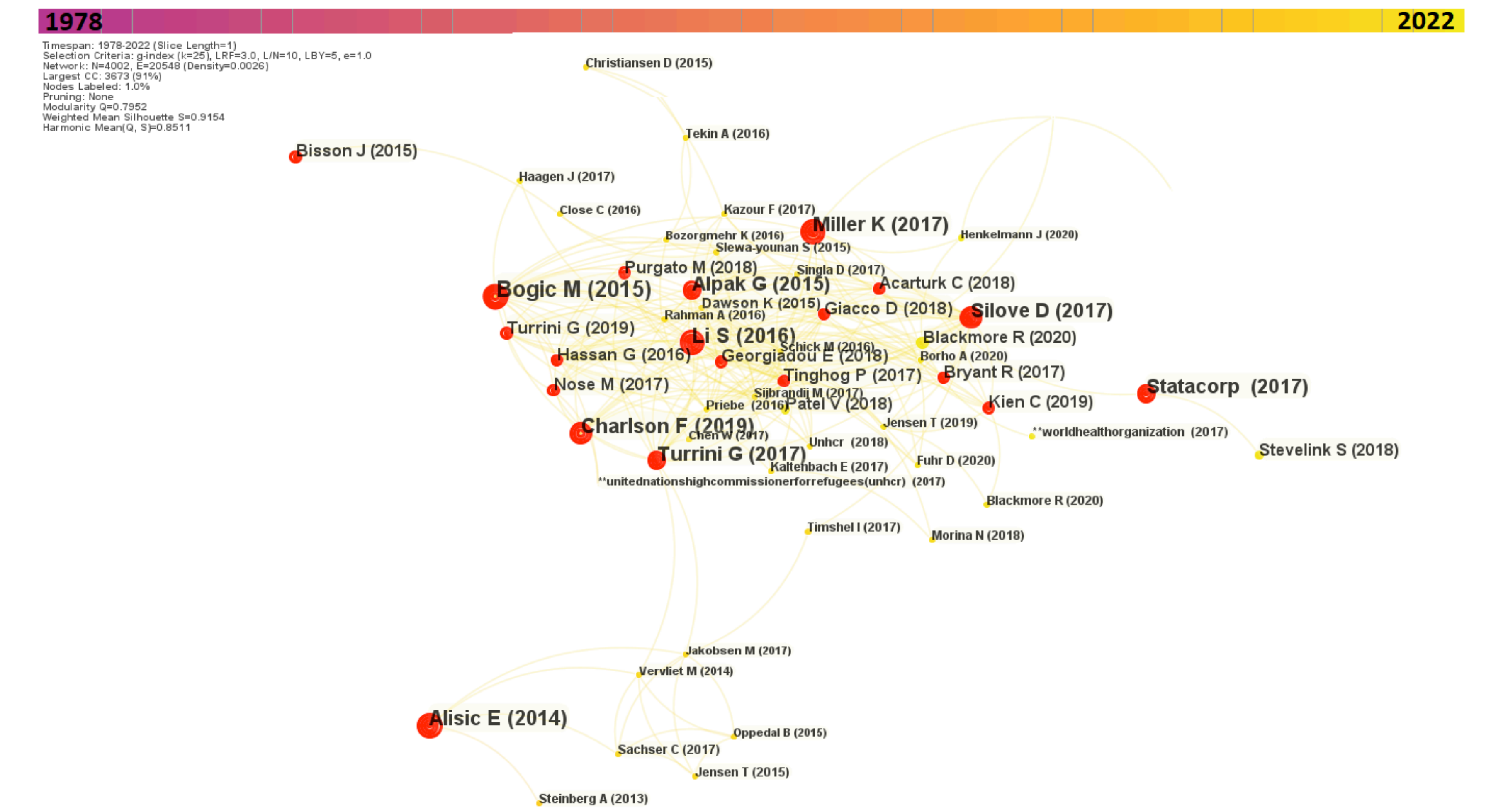


**Articles with highest centrality**

Miller 2017- The mental health of civilians displaced by armed conflict: an ecological model of refugee distress

Silove 2017- The contemporary refugee crisis: an overview of mental health challenges

Bogic 2015- Long-term mental health of war-refugees: a systematic literature review

Alisic 2014- Rates of post-traumatic stress disorder in trauma-exposed children and adolescents: meta-analysis

Li 2016- The Relationship Between Post-Migration Stress and Psychological Disorders in Refugees and Asylum Seekers

<table>
<tr><td>18</td><td>14</td><td>0.999</td><td>2000</td><td>NIBS</td><td>cortical excitability (31.13, 1.0E-4); ect (15.45, 1.0E-4); psychiatric disorders (15.45, 1.0E-4); transcranial magnetic stimulation (15.45, 1.0E-4); transcranial magnetic stimulation (9.44, 0.005)</td></tr>
<tr><td colspan="6">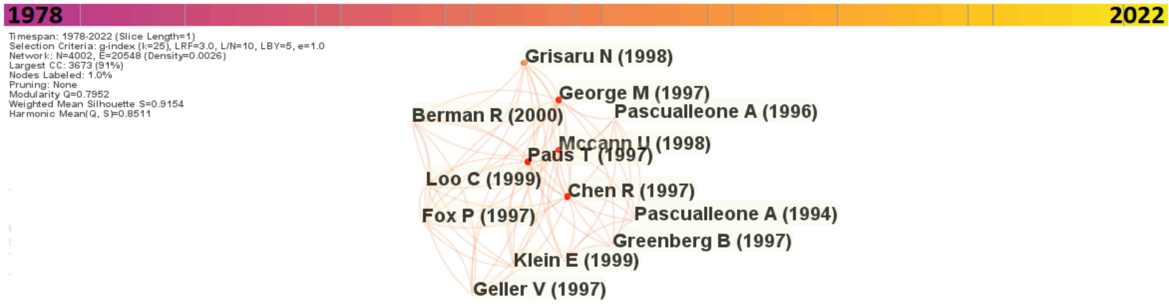
</td></tr>
<tr><td colspan="6">Articles with highest centrality<br>Mccann 1998- Repetitive transcranial magnetic stimulation for posttraumatic stress disorder<br>Grisaru 1998- Effect of transcranial magnetic stimulation in posttraumatic stress disorder: a preliminary study<br>Chen 1997- Depression of motor cortex excitability by low-frequency transcranial magnetic stimulation<br>Geroges 1997 - Mood improvement following daily left prefrontal repetitive transcranial magnetic stimulation in patients with depression: a placebo-controlled crossover trial<br>Paus 1997- Transcranial magnetic stimulation during positron emission tomography: a new method for studying connectivity of the human cerebral cortex</td></tr>
<tr><td>19</td><td>14</td><td>0.999</td><td>1998</td><td>SLEEP DISTURBANCE/ VIETMAN VETERANS</td><td>dreams (25.69, 1.0E-4); sleep (21.51, 1.0E-4); polysomnography (18.07, 1.0E-4); combat veterans (16.18, 1.0E-4); rem sleep (14.57, 0.001)</td></tr>
<tr><td colspan="6">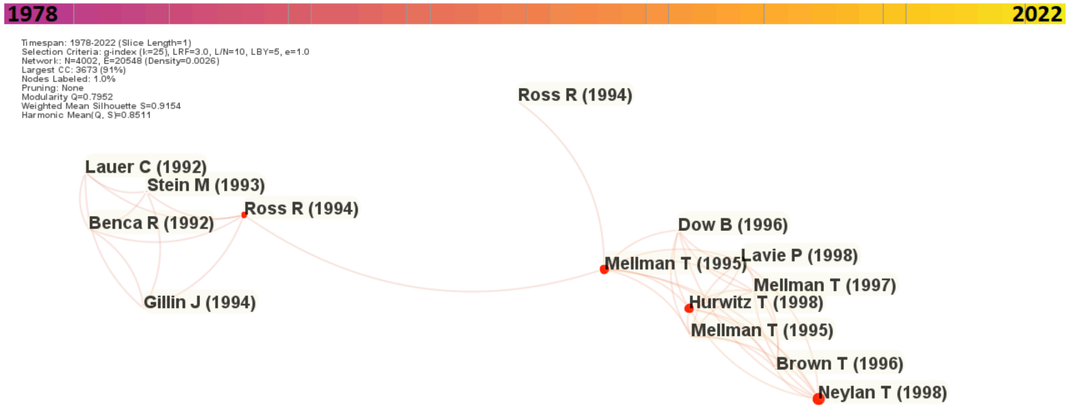
</td></tr>
<tr><td colspan="6">Articles with highest centrality<br>Neylan 1998- Sleep disturbances in the Vietnam generation: findings from a nationally representative sample of male Vietnam veterans<br>Mellman 1995- Sleep events among veterans with combat-related posttraumatic stress disorder<br>Hurwitz 1998- Polysomnographic sleep is not clinically impaired in Vietnam combat veterans with chronic posttraumatic stress disorder<br>Mellman 1995- Sleep disturbance and its relationship to psychiatric morbidity after Hurricane Andrew<br>Ross 1994- Rapid eye movement sleep disturbance in posttraumatic stress disorder.</td></tr>
</table>

| | | | | | |
|---|---|---|---|---|---|
| 20 | 9 | 0.995 | 2007 | PSYCHOSIS/CHILDHOOD TRAUMA | psychosis (34.47, 1.0E-4); sexual abuse (26.16, 1.0E-4); delusions (14.02, 0.001); schizophrenia (12.35, 0.001); hallucinations (11.89, 0.001) |

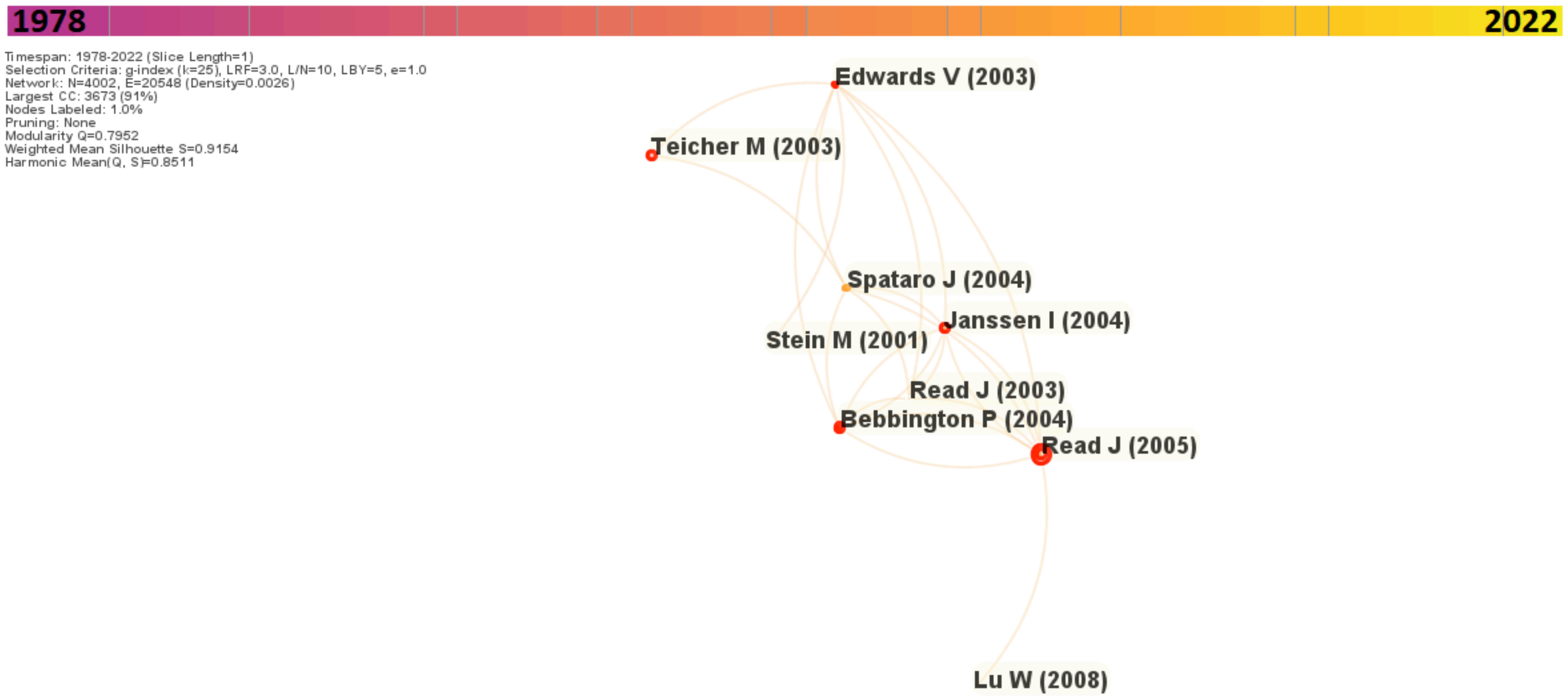


**Articles with highest centrality**

Read 2005- Childhood trauma, psychosis and schizophrenia: a literature review with theoretical and clinical implications

Teicher 2003- The neurobiological consequences of early stress and childhood maltreatment

Bebbington 2004- Psychosis, victimisation and childhood disadvantage Evidence from the second British National Survey Evidence from the second British National Survey of Psychiatric Morbidity

Jannssen 2004- Childhood abuse as a risk factor for psychotic experiences

Edwards 2003- Relationship Between Multiple Forms of Childhood Maltreatment and Adult Mental Health in Community Respondents: Results From the Adverse Childhood Experiences Study

**A. Co-citation reference network with highlight of burstness (2020-2022)**

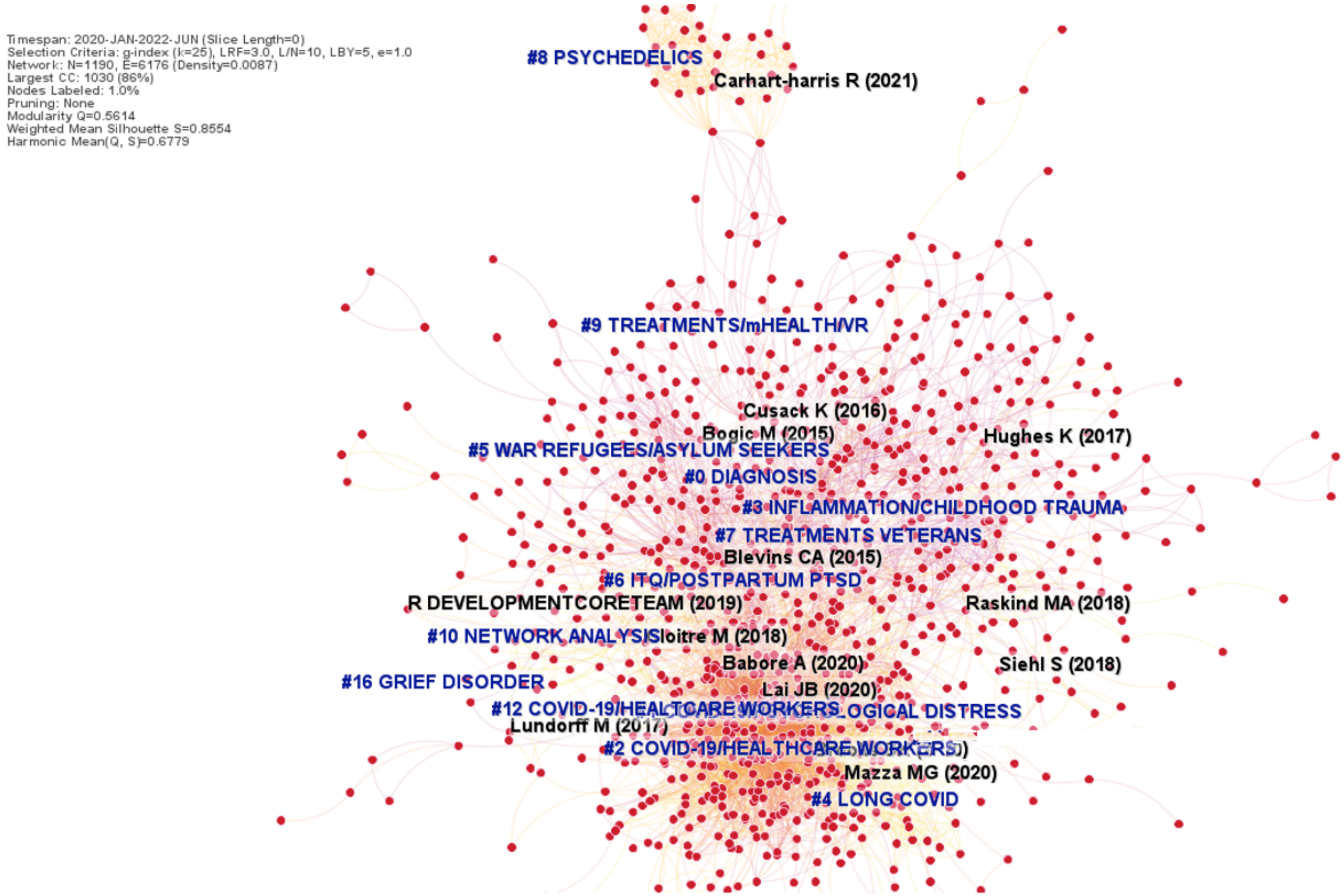


**Supplementary Fig. (6).** Co-cited reference network (2020-2022 time period).

**B. Visualization of the co-citation reference with highlights of clusters (2020-2022)**

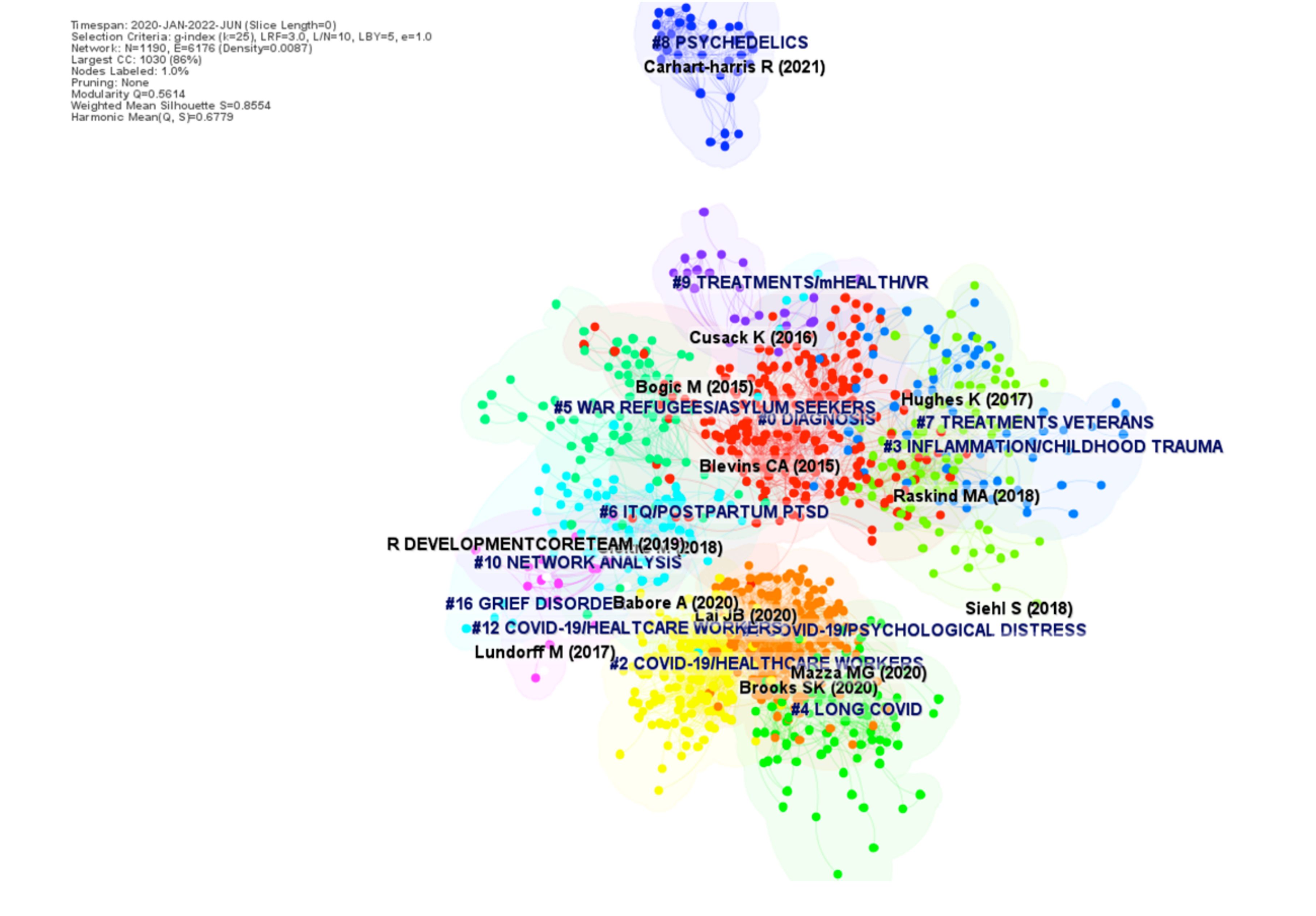

**C. Cluster details by mean year for the co-cited reference network (2020-2022)**

| Cluster ID | Size | Silhouette | Mean (year) | Cluster label | Top Terms (log-likelihood ratio, p-level) |
|---|---|---|---|---|---|
| 0 | 207 | 0.8 | 2020 | DIAGNOSIS | posttraumatic stress disorder (64.82, 1.0E-4); veterans (36.34, 1.0E-4); ptsd (32.6, 1.0E-4); fmri (22.49, 1.0E-4) |

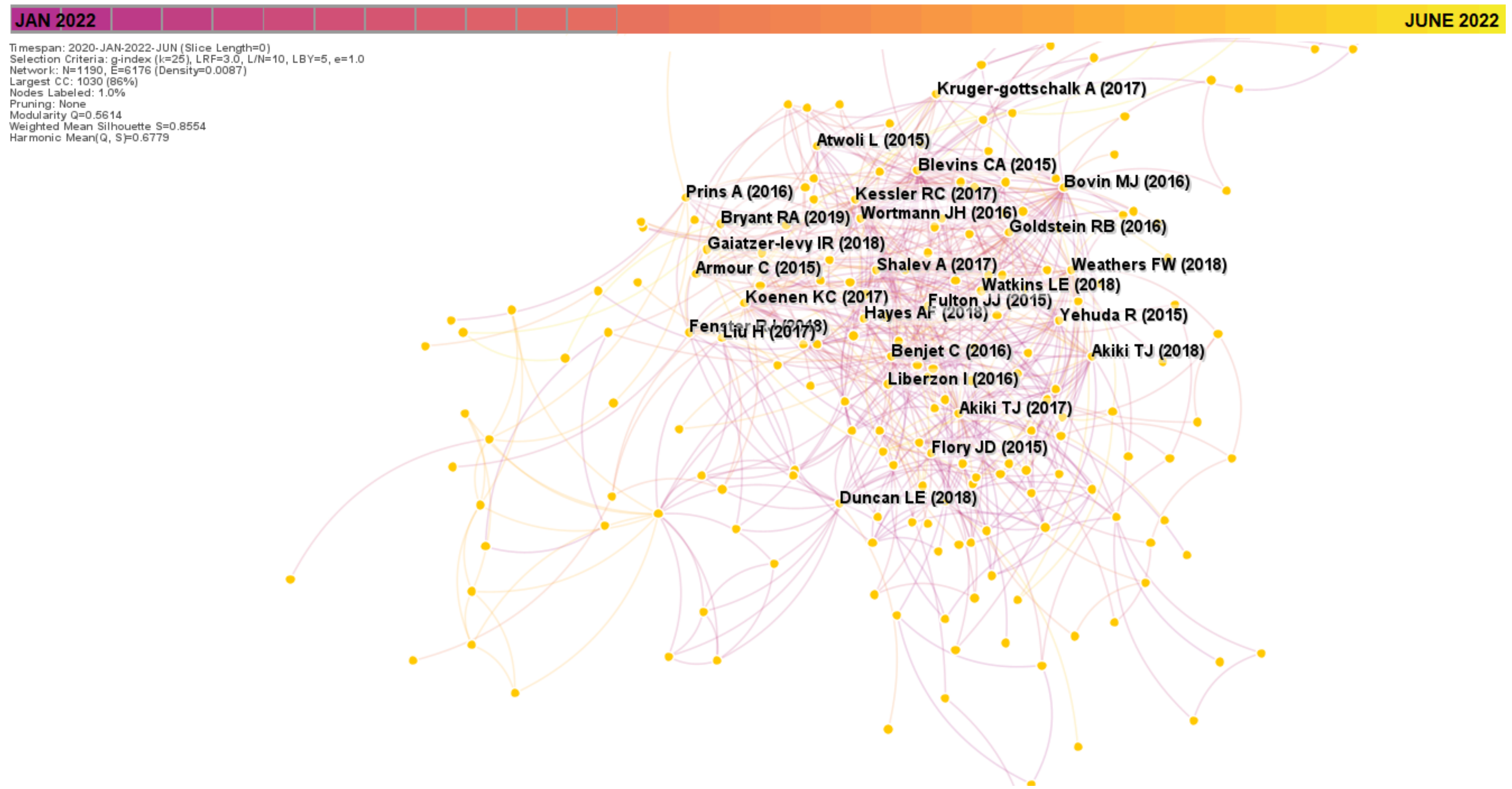


**Articles with highest centrality**

Belvins 2015- The Posttraumatic Stress Disorder Checklist for DSM-5

Bovin 2016- Psychometric properties of the PTSD Checklist for Diagnostic and Statistical Manual of Mental Disorders-Fifth Edition (PCL-5) in veterans

Weathers 2018- The Clinician-Administered PTSD Scale for DSM-5 (CAPS-5)

Kessler 2017- Trauma and PTSD in the WHO World Mental Health Surveys

Koenen 2017- Posttraumatic stress disorder in the World Mental Health Surveys

| | | | | | |
|---|---|---|---|---|---|
| 1 | 184 | 0.778 | 2021 | COVID-19/PSYCHOLOGICAL DISTRESS | covid-19 (113.52, 1.0E-4); anxiety (35.58, 1.0E-4); coronavirus (34.6, 1.0E-4); quarantine (27.08, 1.0E-4); pandemic (25.85, 1.0E-4) |

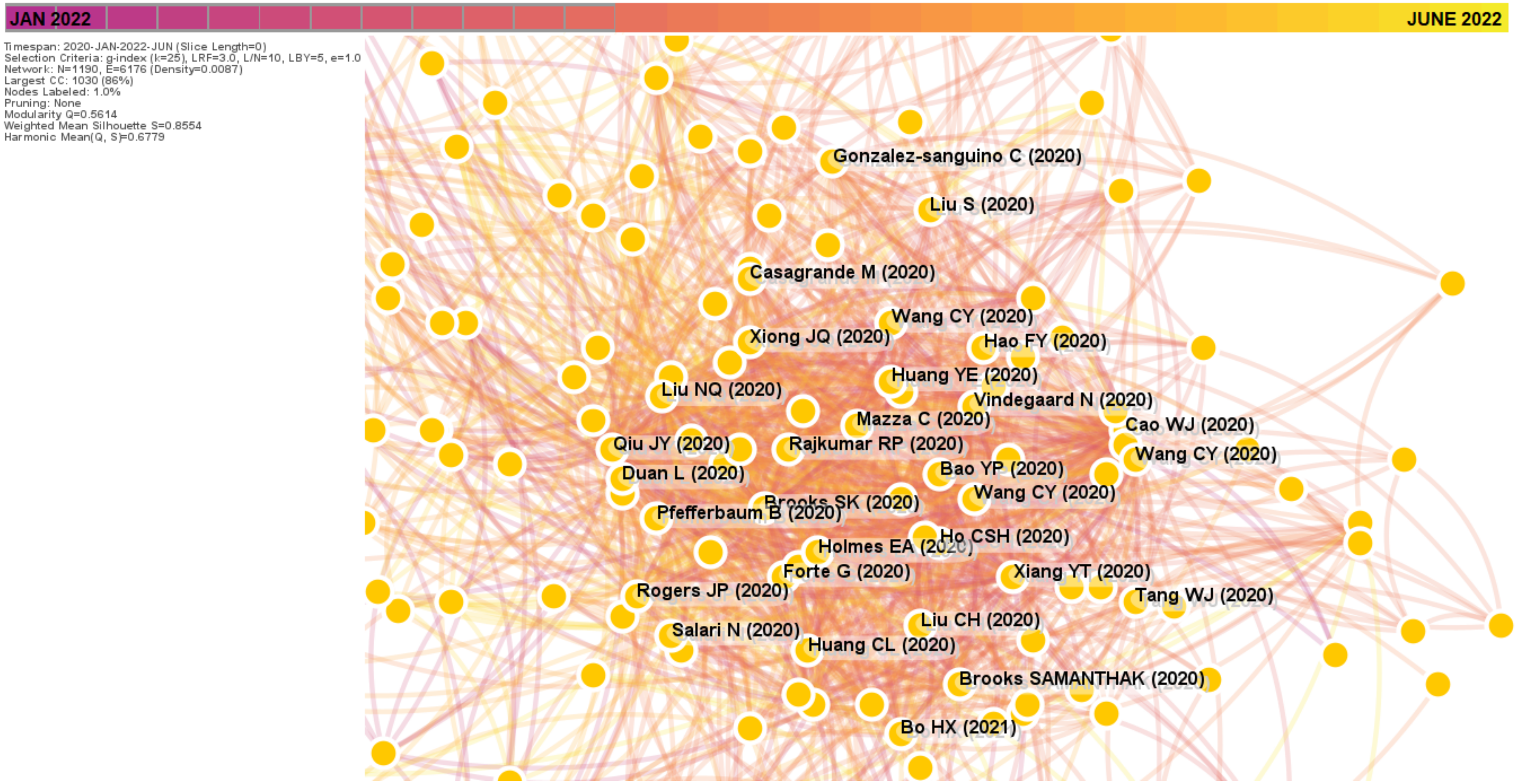


**Articles with highest centrality**

Brooks 2020- The psychological impact of quarantine and how to reduce it

Wang 2020- Immediate Psychological Responses and Associated Factors during the Initial Stage of the 2019 Coronavirus Disease (COVID-19) Epidemic among the General Population in China

Holmes 2020- Multidisciplinary research priorities for the COVID-19 pandemic

Qiu 2020- A nationwide survey of psychological distress among Chinese people in the COVID-19 epidemic: implications and policy recommendations

Liu 2020- Prevalence and predictors of PTSS during COVID-19 outbreak in China hardest-hit areas: Gender differences matter

| | | | | | |
|---|---|---|---|---|---|
| 2 | 153 | 0.749 | 2021 | COVID-19/HEALTHCARE WORKERS | healthcare workers (81.6, 1.0E-4); covid-19 (34.56, 1.0E-4); health care workers (23.84, 1.0E-4); burn-out (20.83, 1.0E-4); nurses (16.89, 1.0E-4) |

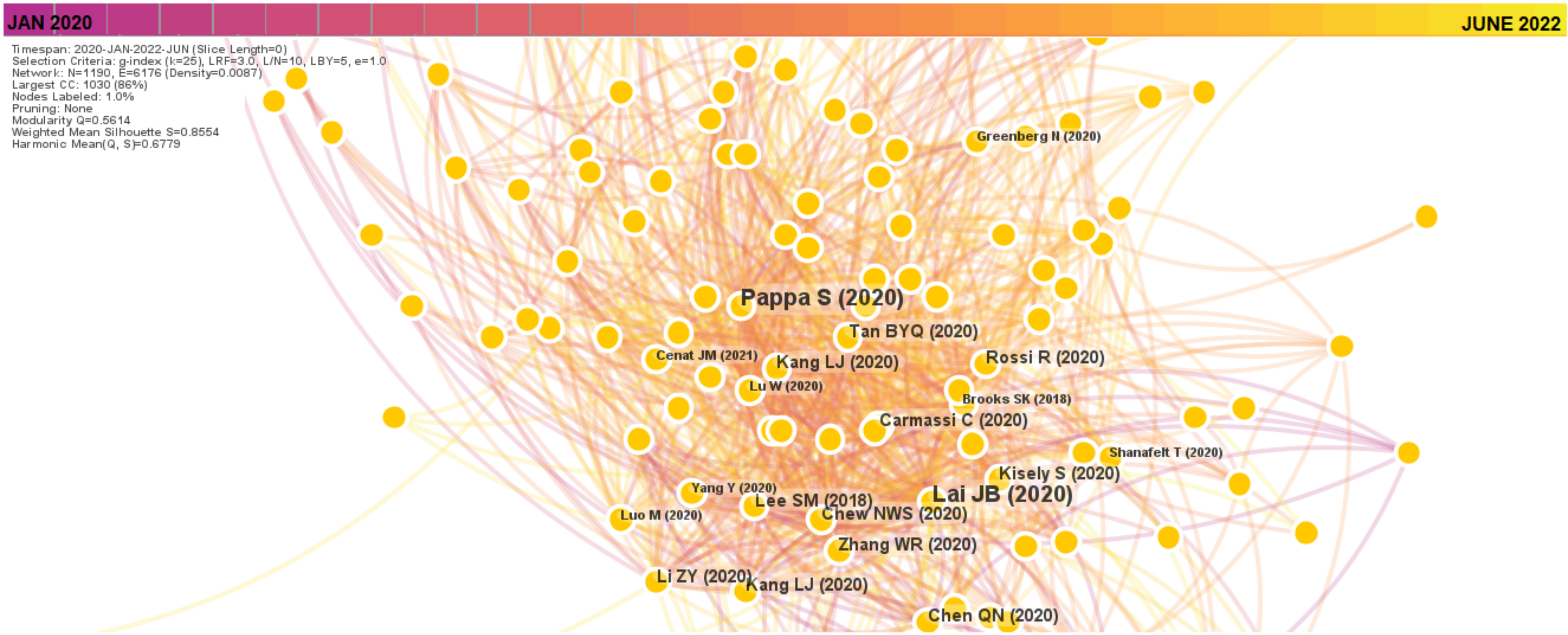


**Articles with highest centrality**

Lai 2020- Factors Associated With Mental Health Outcomes Among Health Care Workers Exposed to Coronavirus Disease 2019

Pappa 2020- Prevalence of depression, anxiety, and insomnia among healthcare workers during the COVID-19 pandemic: A systematic review and meta-analysis

Chew 2020- A multinational, multicentre study on the psychological outcomes and associated physical symptoms amongst healthcare workers during COVID-19 outbreak

Lee 2018- Psychological impact of the 2015 MERS outbreak on hospital workers and quarantined hemodialysis patients

Zhang 2020- Mental Health and Psychosocial Problems of Medical Health Workers during the COVID-19 Epidemic in China

| | | | | | |
|---|---|---|---|---|---|
| 3 | 94 | 0.931 | 2020 | INFLAMMATION/CHILDHOOD TRAUMA | inflammation (32.54, 1.0E-4); microbiome (15.05, 0.001); child abuse (15.05, 0.001); cortisol (14.52, 0.001) |

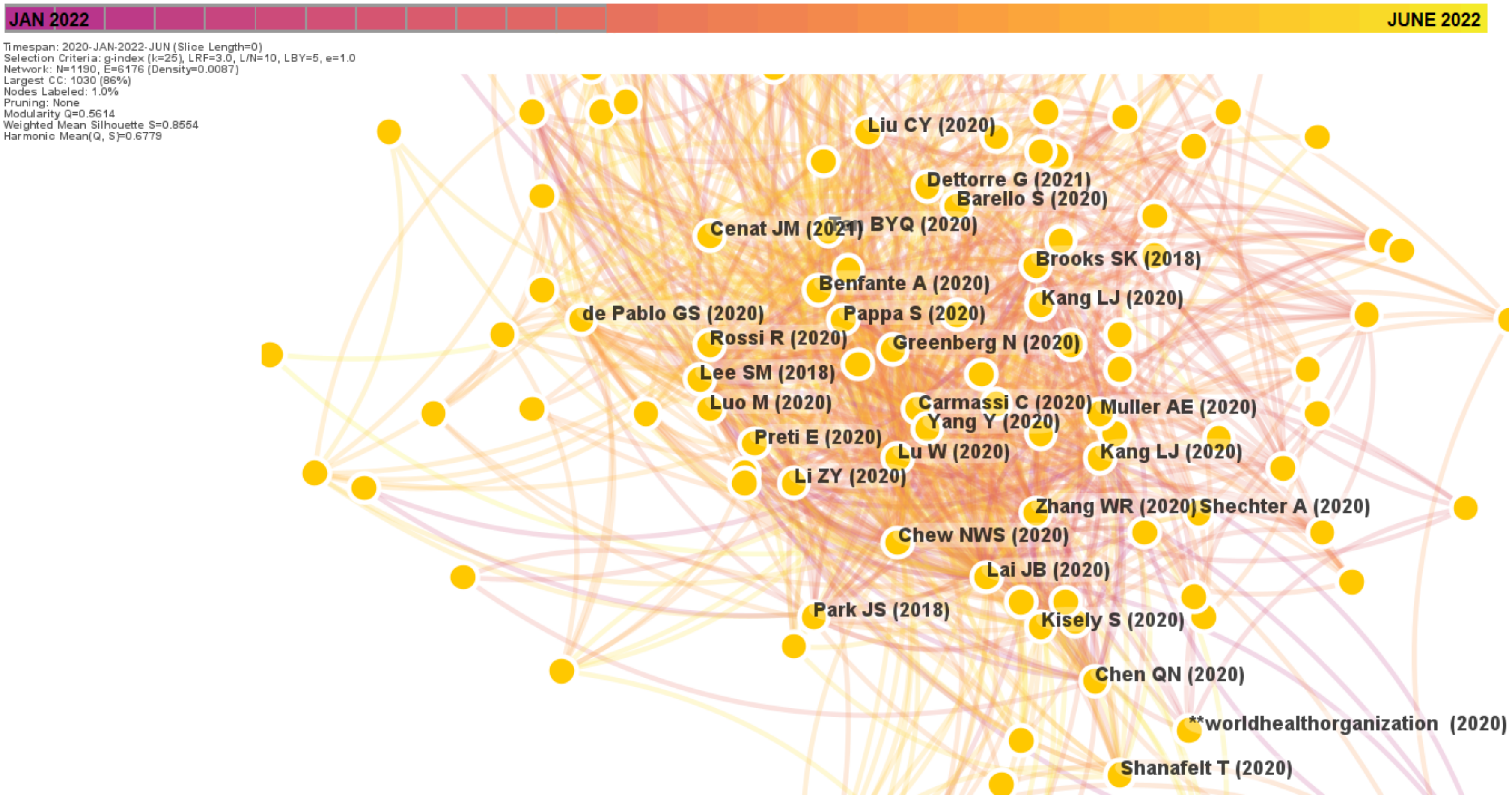


**Articles with highest centrality**

Hughes 2017- The effect of multiple adverse childhood experiences on health: a systematic review and meta-analysis

Baumeister 2016- Childhood trauma and adulthood inflammation: a meta-analysis of peripheral C-reactive protein, interleukin-6 and tumour necrosis factor-α

Passos 2015- Inflammatory markers in post-traumatic stress disorder: a systematic review, meta-analysis, and meta-regression

Odonovan 2015 Elevated Risk for Autoimmune Disorders in Iraq and Afghanistan Veterans with Posttraumatic Stress Disorder

Hori 2019 Inflammation and post□traumatic stress disorder

| | | | | | |
|---|---|---|---|---|---|
| 4 | 85 | 0.905 | 2021 | LONG COVID | sars-cov-2 (30.5, 1.0E-4); long covid (19.95, 1.0E-4); acute respiratory distress syndrome (14.95, 0.001); severe acute respiratory syndrome coronavirus 2 (14.95, 0.001); trauma (11.13, 0.001) |

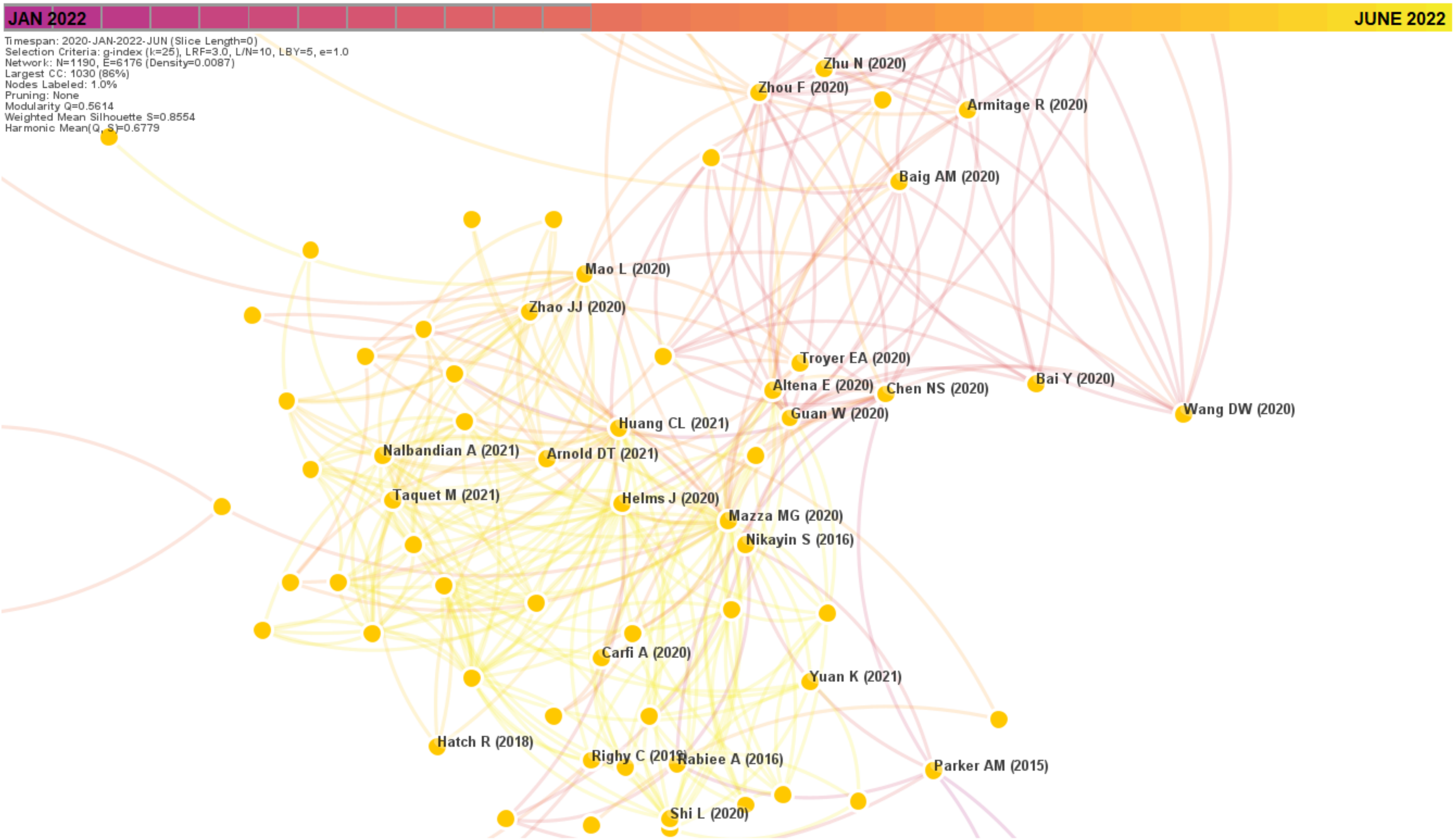


**Articles with highest centrality**

Huang 2021- 6-month consequences of COVID-19 in patients discharged from hospital: a cohort study

Mazza 2020- Anxiety and depression in COVID-19 survivors

Mao 2020- Neurologic Manifestations of Hospitalized Patients With Coronavirus Disease 2019 in Wuhan, China

Chen 2020- Epidemiological and clinical characteristics of 99 cases of 2019 novel coronavirus pneumonia in Wuhan, China: a descriptive study

Righy 2019- Prevalence of post-traumatic stress disorder symptoms in adult critical care survivors: a systematic review and meta-analysis

| | | | | | |
|---|---|---|---|---|---|
| 5 | 75 | 0.925 | 2020 | WAR REFUGEES/ASYLUM SEEKERS | asylum seekers (57.46, 1.0E-4); refugees (52.06, 1.0E-4); refugee (30.6, 1.0E-4); covid-19 (29.41, 1.0E-4); syria (20.8, 1.0E-4) |

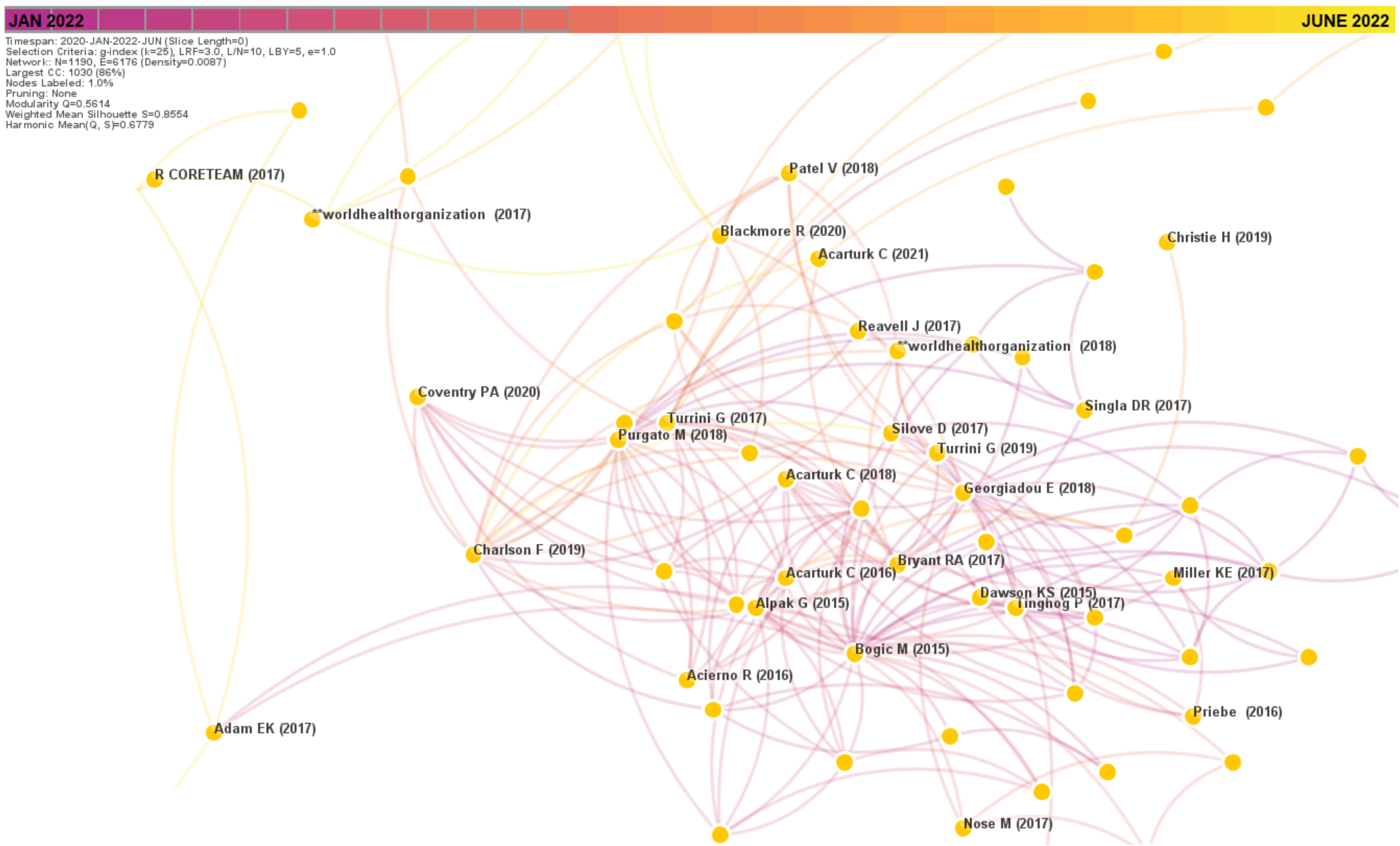


**Articles with highest centrality**

Bogic 2015- Long-term mental health of war-refugees: a systematic literature review

Charlson 2019- New WHO prevalence estimates of mental disorders in conflict settings: a systematic review and meta-analysis

Georgiadou 2018- Prevalence of Mental Distress Among Syrian Refugees With Residence Permission in Germany: A Registry-Based Study

Turrini 2017- Common mental disorders in asylum seekers and refugees: umbrella review of prevalence and intervention studies

Blackmore 2020 The prevalence of mental illness in refugees and asylum seekers: A systematic review and meta-analysis

| | | | | | |
|---|---|---|---|---|---|
| 6 | 75 | 0.871 | 2021 | COMPLEX PTSD/POSTPARTUM PTSD | icd-11 (32.77, 1.0E-4); covid-19 (32.5, 1.0E-4); postpartum (24.25, 1.0E-4); complex ptsd (18.21, 1.0E-4); childbirth (16.24, 1.0E-4) |

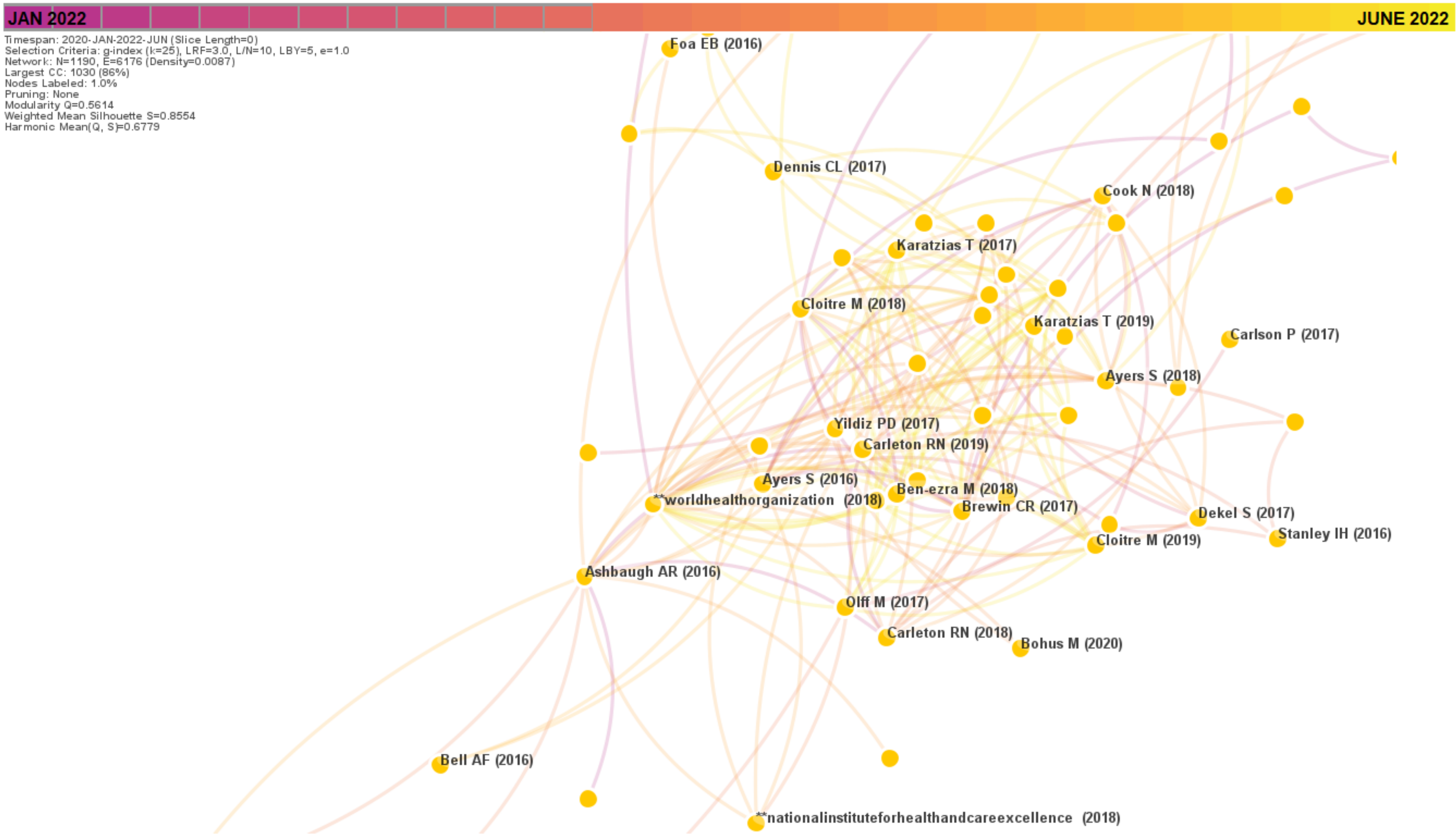


**Articles with highest centrality**

Yildiz 2017- The prevalence of posttraumatic stress disorder in pregnancy and after birth: A systematic review and meta-analysis

Cloitre 2018- The International Trauma Questionnaire: development of a self-report measure of ICD-11 PTSD and complex PTSD

Ashbaugh 2016 - Psychometric Validation of the English and French Versions of the Posttraumatic Stress Disorder Checklist for DSM-5 (PCL-5)

Brewin 2017- A review of current evidence regarding the ICD-11 proposals for diagnosing PTSD and complex PTSD

Ayers 2016 - The aetiology of post-traumatic stress following childbirth: a meta-analysis and theoretical framework

| | | | | | |
|---|---|---|---|---|---|
| 7 | 69 | 0.96 | 2020 | TREATMENTS VETERANS | terrorism (13.45, 0.001); emergency medical service providers challenges (6.72, 0.01); mental health services (6.72, 0.01); s-ketamine (6.72, 0.01) |

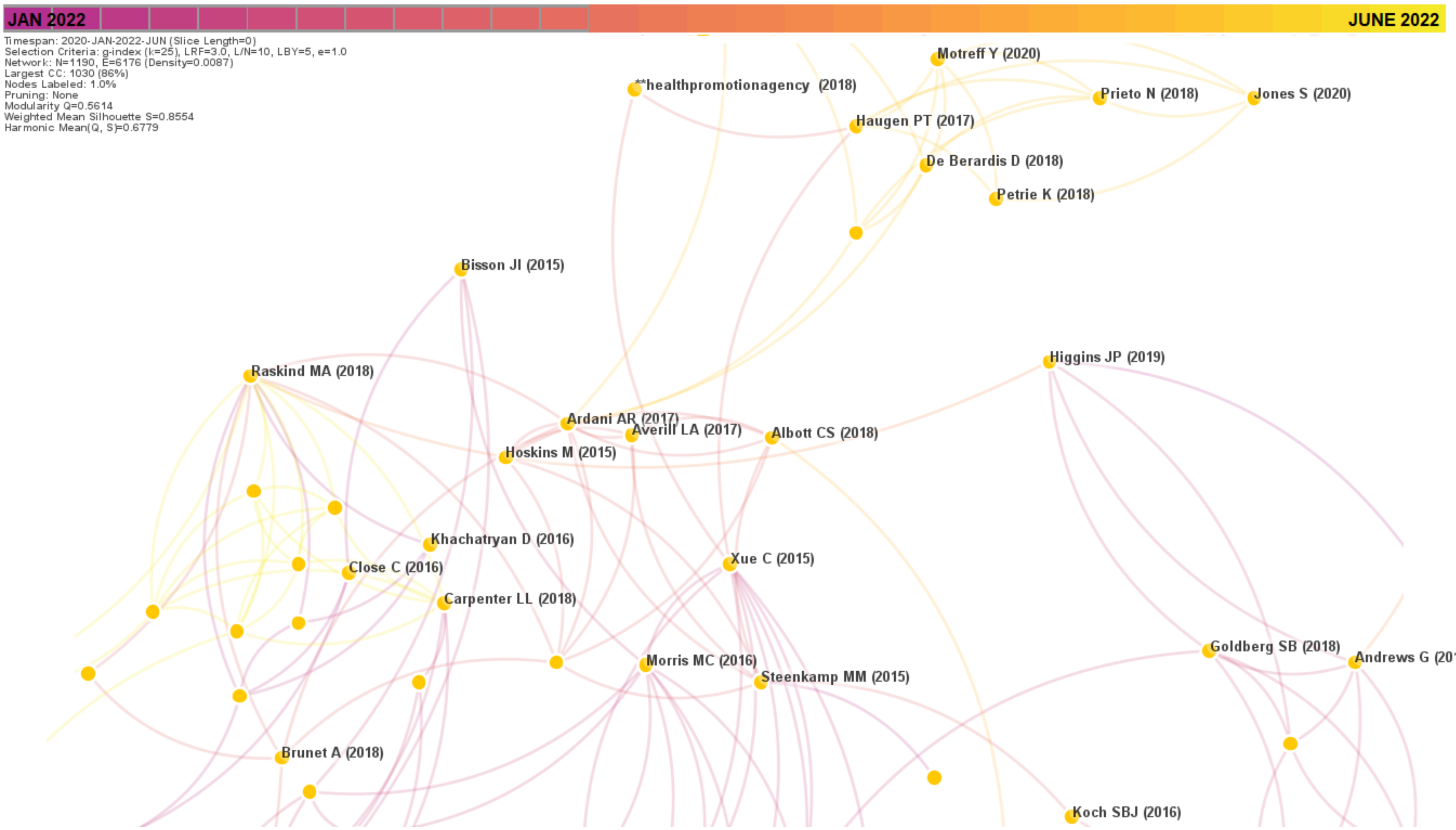


**Articles with highest centrality**

Raskind 2018- Trial of Prazosin for Post-Traumatic Stress Disorder in Military Veterans

Xue 2019- A Meta-Analysis of Risk Factors for Combat-Related PTSD among Military Personnel and Veterans

Bisson 2015- Post-Traumatic Stress Disorder

Steenkam 2015- Psychotherapy for Military-Related PTSD A Review of Randomized Clinical Trials

Petrie 2018- Prevalence of PTSD and common mental disorders amongst ambulance personnel: a systematic review and meta-analysis

| | | | | | |
|---|---|---|---|---|---|
| 8 | 38 | 0.996 | 2021 | PSYCHEDELICS | psilocybin (53.9, 1.0E-4); nsduh (26.74, 1.0E-4); psychedelics (26.74, 1.0E-4); ayahuasca (17.78, 1.0E-4); psychedelic (17.78, 1.0E-4) |

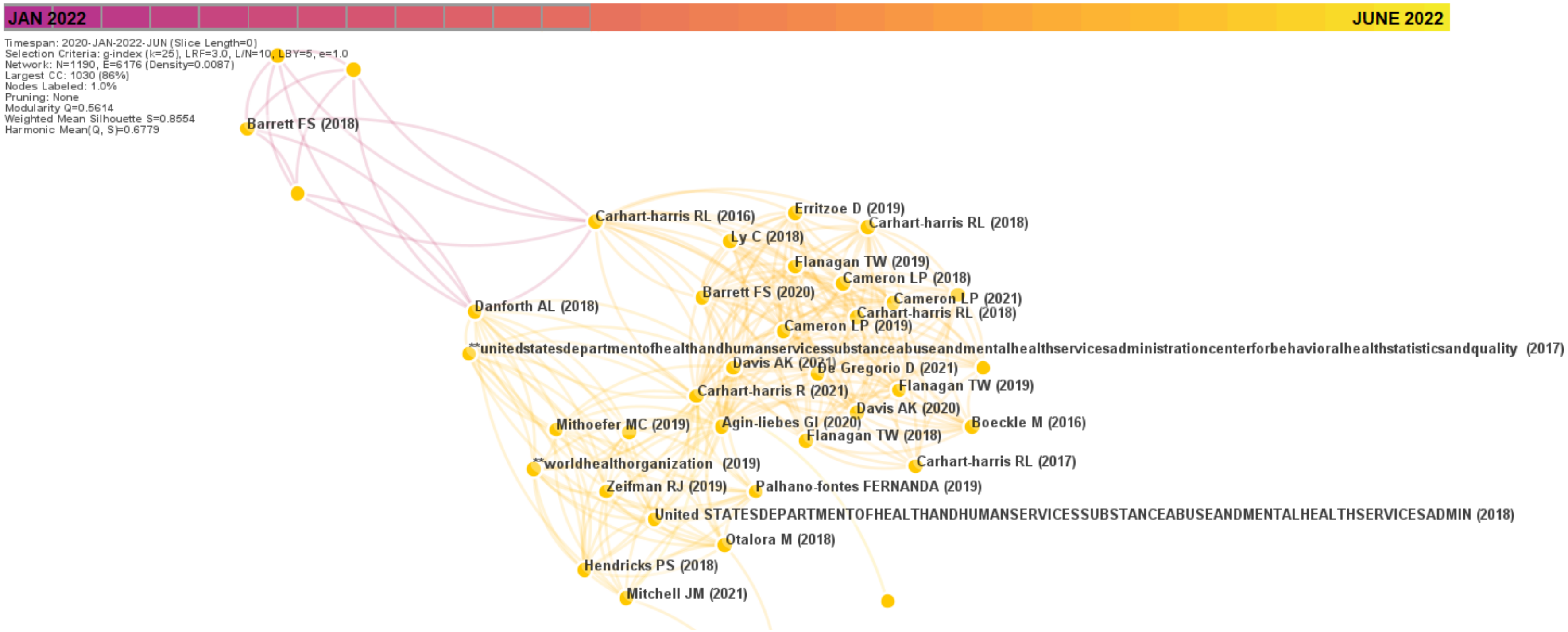


**Articles with highest centrality**

Mitchell 2021- MDMA-assisted therapy for severe PTSD: a randomized, double-blind, placebo-controlled phase 3 study

Carhart-Harris 2016- Psilocybin with psychological support for treatment-resistant depression: an open-label feasibility study

Danforth 2018- Reduction in social anxiety after MDMA-assisted psychotherapy with autistic adults: a randomized, double-blind, placebo-controlled pilot study

Carhart-Harris 2021- Trial of Psilocybin versus Escitalopram for Depression

Davis 2021- Effects of Psilocybin-Assisted Therapy on Major Depressive Disorder A Randomized Clinical Trial

| | | | | | |
|---|---|---|---|---|---|
| 9 | 24 | 0.981 | 2020 | TREATMENTS/mHEALTH/VR | virtual reality (20.06, 1.0E-4); mhealth (13.99, 0.001); therapy (12.29, 0.001); smartphone (8.87, 0.005); chronic disease (8.87, 0.005) |

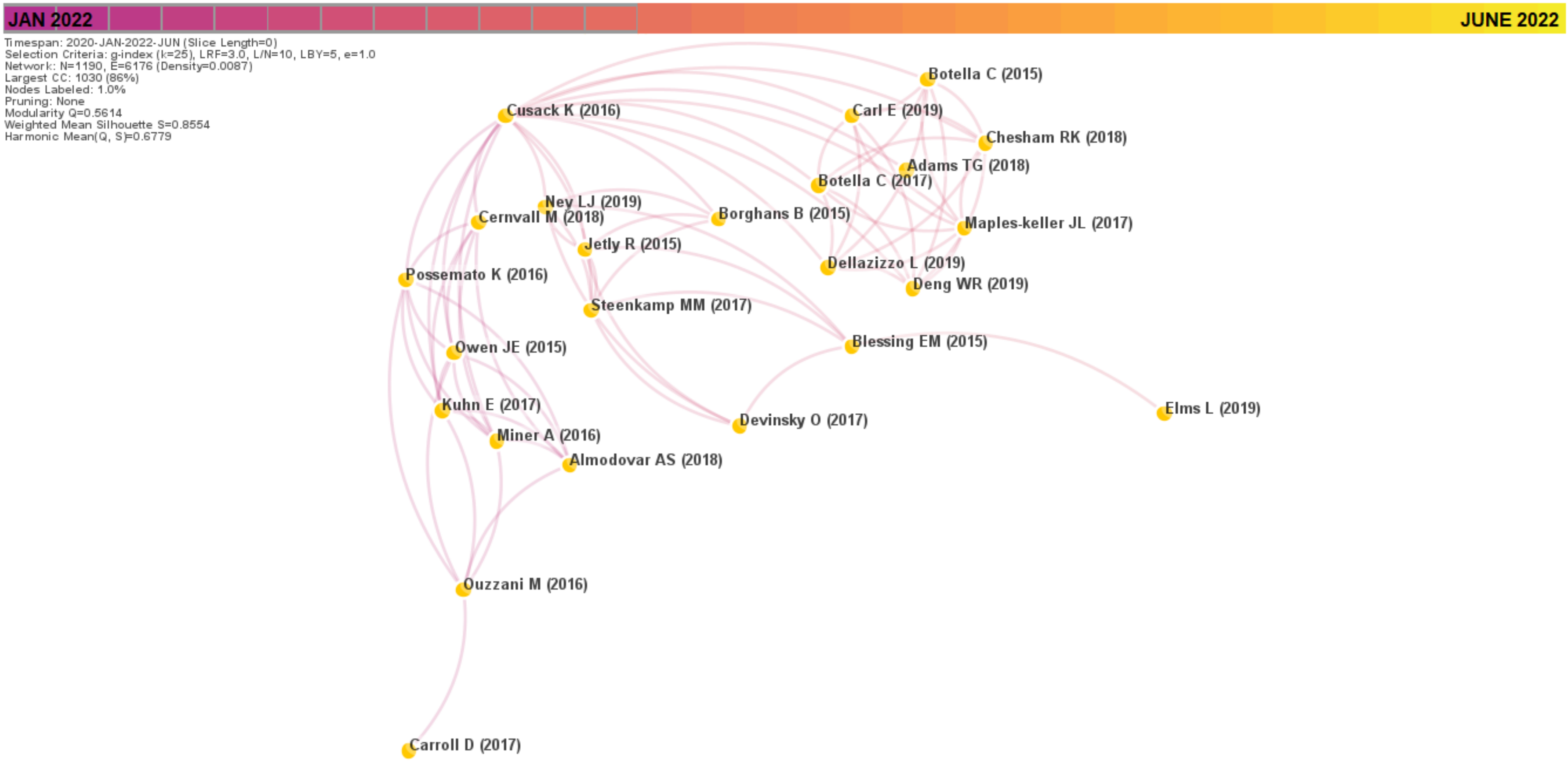


**Articles with highest centrality**

Cusack 2016- Psychological treatments for adults with posttraumatic stress disorder: A systematic review and meta-analysis

Jetly 2015- The efficacy of nabilone, a synthetic cannabinoid, in the treatment of PTSD-associated nightmares: A preliminary randomized, double-blind, placebo-controlled cross-over design study

Carl 2019- Virtual reality exposure therapy for anxiety and related disorders: A meta-analysis of randomized controlled trials

Owen 2015- mHealth in the Wild: Using Novel Data to Examine the Reach, Use, and Impact of PTSD Coach

| | | | | | |
|---|---|---|---|---|---|
| 10 | 18 | 0.947 | 2021 | NETWORK ANALYSIS | network analysis (38.83, 1.0E-4); covid-19 (10.36, 0.005); comorbidity (9.4, 0.005); prolonged exposure (7.99, 0.005); hallucinations (7.99, 0.005) |

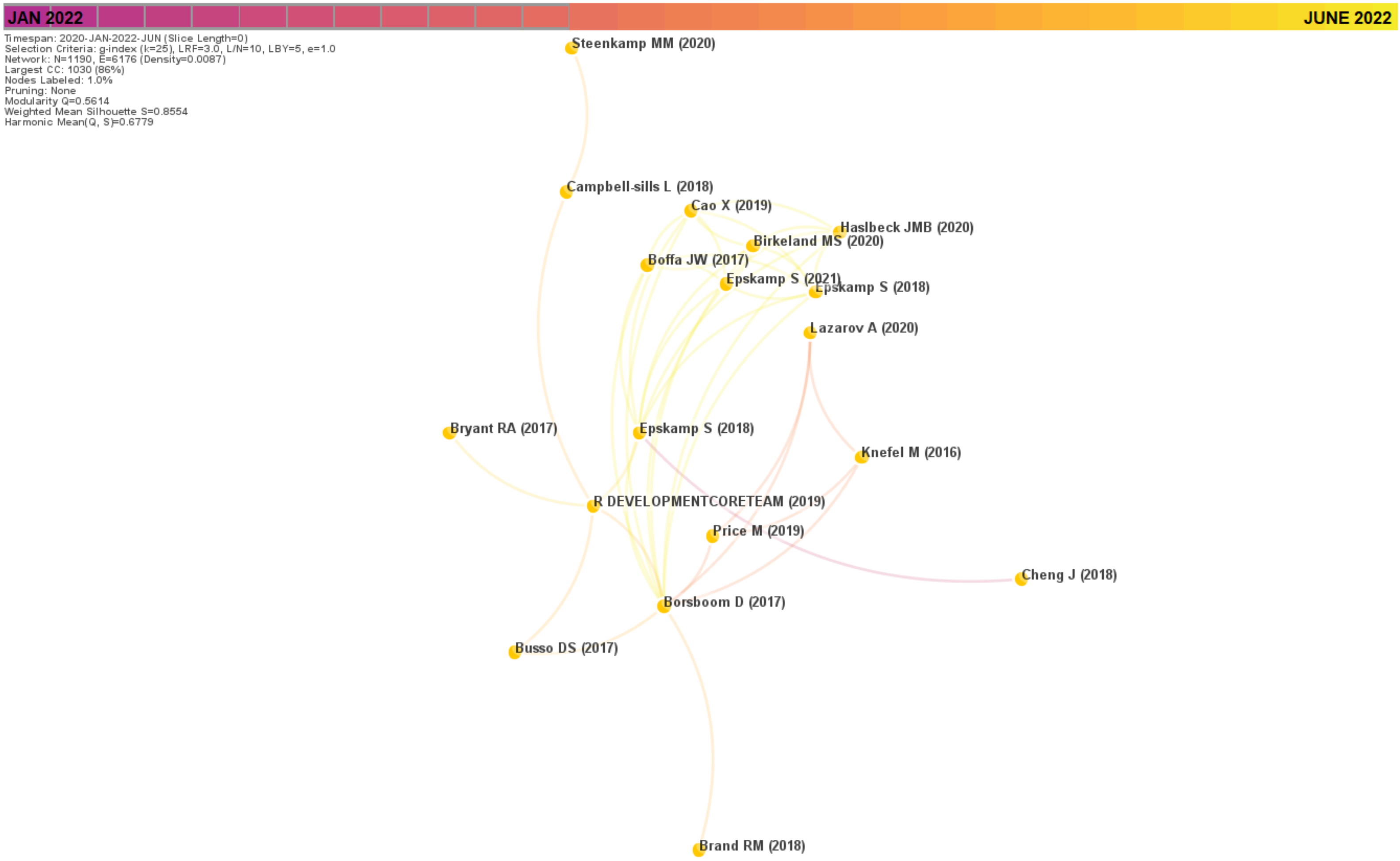


**Articles with highest centrality**

Borsboom 2017- A network theory of mental disorders

Epskamp 2018- Estimating psychological networks and their accuracy: A tutorial paper

Lazarov 2020- Symptom structure of PTSD and co-morbid depressive symptoms - a network analysis of combat veteran patients

Bryant 2017- Mental Health and Social Networks After Disaster

Campbell-Sills 2017- Predictive validity and correlates of self-assessed resilience among U.S. Army soldiers

| | | | | | |
|---|---|---|---|---|---|
| 12 | 5 | 0.994 | 2022 | COVID-19/HEALTCARE WORKERS | regression analysis (10.94, 0.001); cross-sectional studies (10.94, 0.001); occupational change (10.94, 0.001); nursing staff (10.94, 0.001); psychosocial stress (10.94, 0.001) |

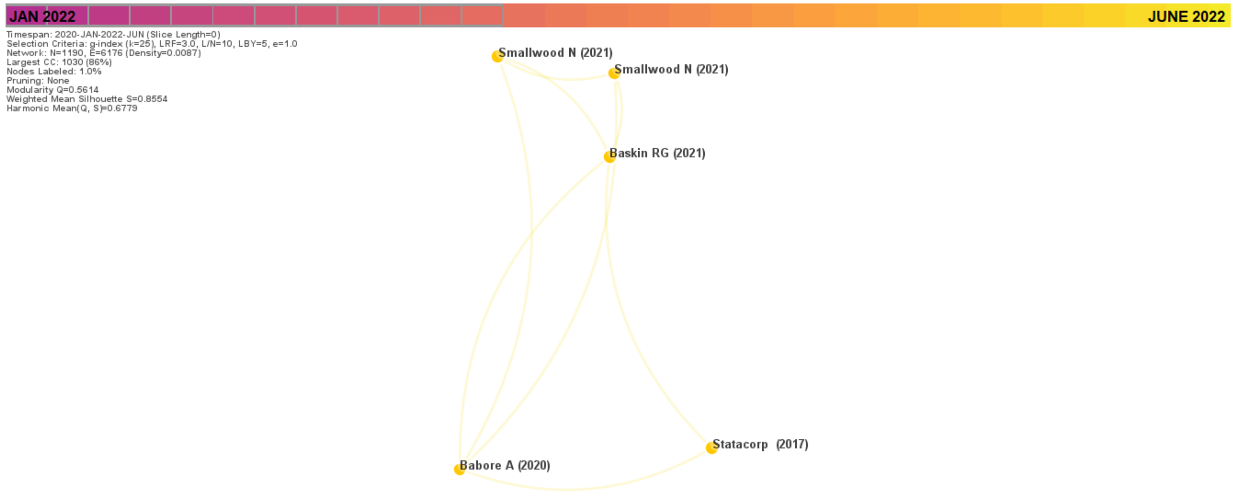


**Articles with highest centrality**

Babore 2020- Psychological effects of the COVID-2019 pandemic: Perceived stress and coping strategies among healthcare professionals

Smallwood 2021- Occupational Disruptions during the COVID-19 Pandemic and Their Association with Healthcare Workers' Mental Health

Smallwood 2021- High levels of psychosocial distress among Australian frontline healthcare workers during the COVID-19 pandemic: a cross-sectional survey

| | | | | | |
|---|---|---|---|---|---|
| 16 | 3 | 0.996 | 2020 | GRIEF DISORDER | bereavement (21.61, 1.0E-4); complicated grief (10.61, 0.005); outcomes (7.85, 0.01); latent class analysis (7.85, 0.01); death (6.81, 0.01) |

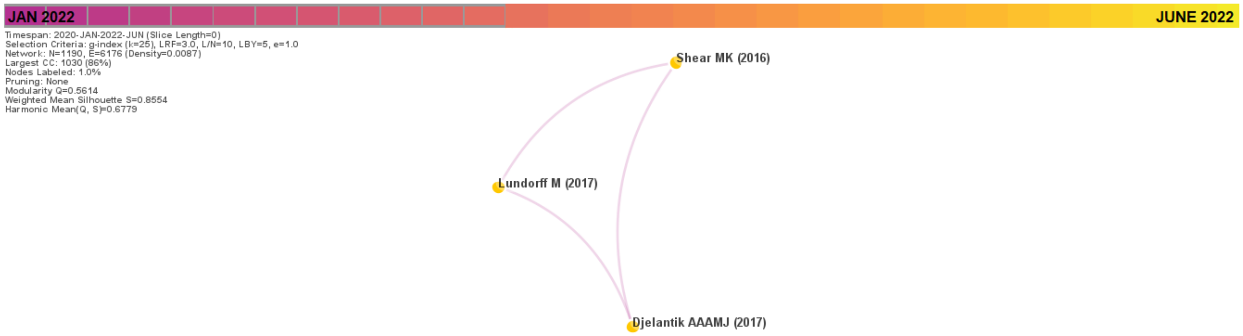


**Articles with highest centrality**

Shear 2016- Optimizing Treatment of Complicated Grief: A Randomized Clinical Trial

Djelantik 2017- Symptoms of prolonged grief, post-traumatic stress, and depression after loss in a Dutch community sample: A latent class analysis

Lundorff 2017- Prevalence of prolonged grief disorder in adult bereavement: A systematic review and meta-analysis

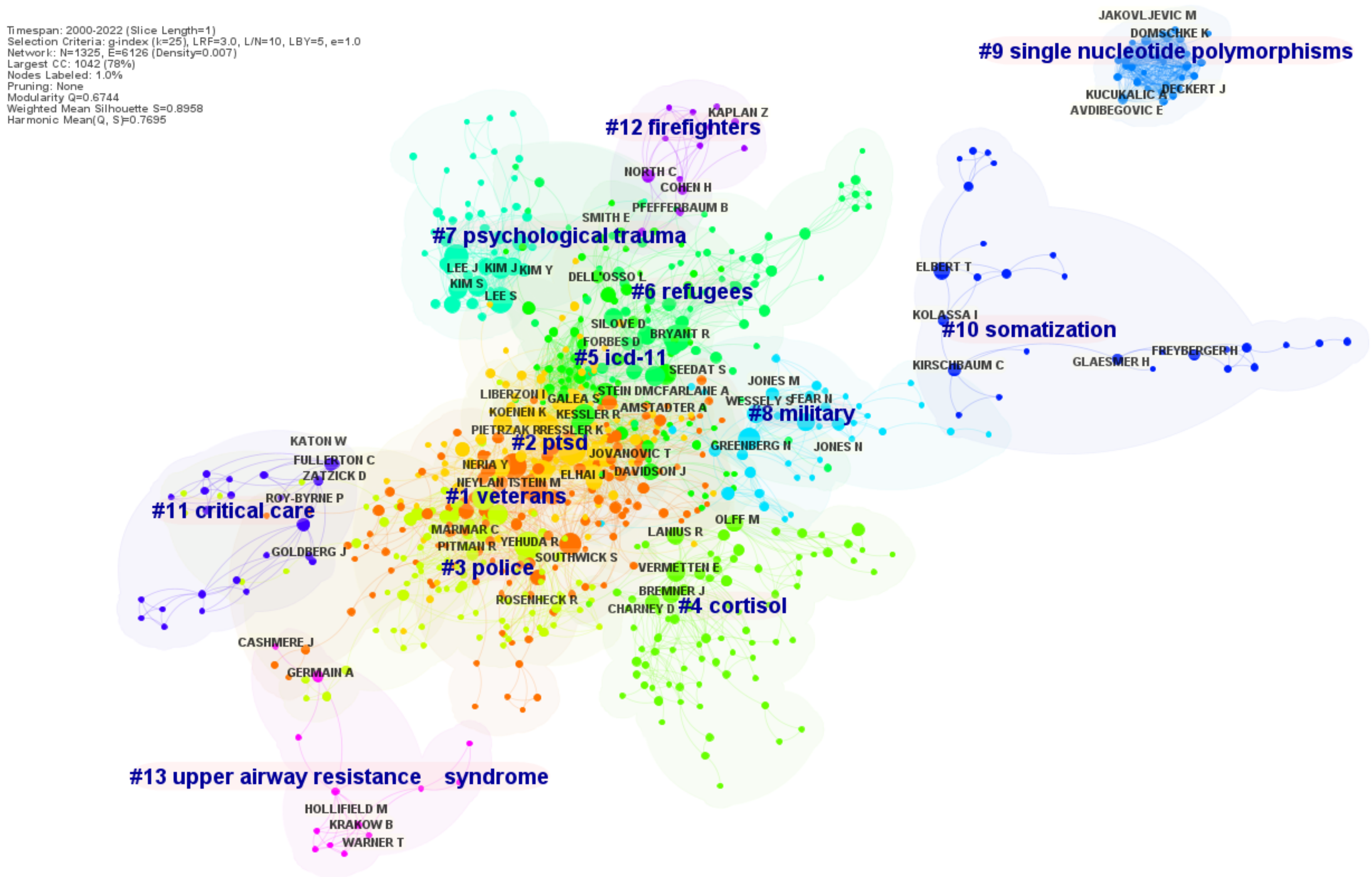


**Supplementary Fig. (7).** Co-cited co-authors network (2000-2022 time period) with cluster details.

| Cluster Details | | | | |
|---|---|---|---|---|
| **Cluster ID** | **Size** | **Silhouette Score** | **Mean Year** | **Top Terms (Log-likelihood Ratio, p-levels)** |
| 1 | 149 | 0.857 | 2011 | veterans (82.24, 1.0E-4); posttraumatic stress disorder (32.62, 1.0E-4); epidemiology (26.87, 1.0E-4); comorbidity (24.01, 1.0E-4); protective factors (19.87, 1.0E-4) |
| 2 | 125 | 0.855 | 2013 | ptsd (44.15, 1.0E-4); fear-potentiated startle (37.87, 1.0E-4); covid-19 (36.8, 1.0E-4); women (24.29, 1.0E-4); startle (22.7, 1.0E-4) |
| 3 | 103 | 0.888 | 2008 | police (29.39, 1.0E-4); propranolol (29.39, 1.0E-4); holocaust (24.48, 1.0E-4); conditioning (20.75, 1.0E-4); cortisol (20.65, 1.0E-4) |
| 4 | 88 | 0.915 | 2007 | cortisol (29.15, 1.0E-4); borderline personality disorder (21.57, 1.0E-4); public safety personnel (20.14, 1.0E-4); hpa-axis (20.14, 1.0E-4); covid-19 (18.94, 1.0E-4) |
| 5 | 85 | 0.867 | 2010 | icd-11 (28.95, 1.0E-4); complex ptsd (20.75, 1.0E-4); cptsd (19.58, 1.0E-4); parents (19.58, 1.0E-4); veterans (14.85, 0.001) |
| 6 | 75 | 0.872 | 2012 | refugees (83.91, 1.0E-4); refugee (27.13, 1.0E-4); adapt (24.66, 1.0E-4); covid-19 (19.99, 1.0E-4); war (19.72, 1.0E-4) |
| 7 | 49 | 0.953 | 2013 | psychological trauma (25.34, 1.0E-4); korea (25.34, 1.0E-4); sewol ferry disaster (25.34, 1.0E-4); ptsd (18.85, 1.0E-4); firefighters (16.52, 1.0E-4) |
| 8 | 38 | 0.964 | 2009 | military (64.87, 1.0E-4); alcohol misuse (40.52, 1.0E-4); deployment (39.43, 1.0E-4); military personnel (25.99, 1.0E-4); mental health (14.94, 0.001) |
| 9 | 30 | 0.998 | 2017 | single nucleotide polymorphisms (10.99, 0.001); slc6a3 (dat) gene (10.99, 0.001); drd4 vntr exon 3 (10.99, 0.001); rar-related orphan receptor a (10.99, 0.001); oxytocin receptor gene (10.99, 0.001) |
| 10 | 27 | 0.997 | 2009 | somatization (29.75, 1.0E-4); narrative exposure therapy (21.03, 1.0E-4); general population (16.59, 1.0E-4); somatization disorder (14.01, 0.001); syrian refugees (14.01, 0.001) |
| 11 | 23 | 0.984 | 2008 | critical care (30.97, 1.0E-4); outcome assessment (health care) (19.22, 1.0E-4); telehealth (13.71, 0.001); telepsychiatry (13.71, 0.001); military medicine (11.65, 0.001) |
| 12 | 14 | 0.994 | 2006 | firefighters (21.77, 1.0E-4); animal model (20.48, 1.0E-4); world trade center (20.27, 1.0E-4); fibromyalgia (13.79, 0.001); low-resolution electrical tomographic analysis (10.22, 0.005) |
| 13 | 13 | 0.987 | 2002 | upper airway resistance syndrome (13.26, 0.001); upper airway resistance (13.26, 0.001); sleep-disordered breathing (13.26, 0.001); obstructive sleep apnea (10.49, 0.005); sleep apnea (9.45, 0.005) |

**Supplementary Table 1. The top 10 most cited journals (2000-2022).**

| Journals with Most Articles | Initial Year | Impact Factor (2020-2021) | Total Articles (%) | Total Articles | Journal H-index | Journals with Most Co-citations | Total Citations |
|---|---|---|---|---|---|---|---|
| 1. Journal of Affective Disorders | 1979 | 4.83 | 2.1 | 919 | 205 | 1. Arch Gen Psychiatry (JAMA) | 21805 |
| 2. Psychiatry Research | 1979 | 3.22 | 1.9 | 800 | 147 | 2. American Journal of Psychiatry | 17713 |
| 3. Journal of Nervous and Mental Disease | 1874 | 1.73 | 1.7 | 725 | 128 | 3. Journal of Traumatic Stress | 14027 |
| 4. PLOS ONE | 2006 | 3.58 | 1.6 | 693 | 367 | 4. Biological Psychiatry | 12013 |
| 5. Frontiers in Psychiatry | 2010 | 5.43 | 1.4 | 630 | 81 | 5. PLOS ONE | 11144 |
| 6. Military Medicine | 1954 | 1.56 | 1.3 | 566 | 70 | 6. Journal of Clinical Psychiatry | 9817 |
| 7. Biological Psychiatry | 1969 | 12.8 | 1.2 | 539 | 333 | 7. Journal of Consulting and Clinical Psychology | 9749 |
| 8. Journal of Psychiatric Research | 1961 | 4.79 | 1.2 | 523 | 144 | 8. Journal of Nervous and Mental Disease | 9420 |
| 9. Depression and Anxiety | 1996 | 8.1 | 1.2 | 513 | 138 | 9. The LANCET | 9272 |
| 10. Psychological Medicine | 1970 | 7.7 | 1.1 | 492 | 220 | 10. Psychiatry Research | 9228 |

**Supplementary Table 2. Burstness analysis for countries, institutions, authors, references and keywords (1978-2022, 2000-2022 and 2020-2022).**

**A. Top 25 countries sorted by beginning year of citation burst (1978-2022)**

| Countries | Year | Strength | Begin | End | 1978 - 2022 |
|---|---|---|---|---|---|
| USA | 1978 | 57.25 | 1981 | 1993 | |
| ISRAEL | 1978 | 21.05 | 1986 | 2002 | |
| RUSSIA | 1978 | 5.33 | 1991 | 2006 | |
| CROATIA | 1978 | 20.06 | 1999 | 2012 | |
| BOSNIA & HERCEG | 1978 | 13.41 | 2006 | 2013 | |
| SERBIA | 1978 | 12.04 | 2009 | 2013 | |
| BRAZIL | 1978 | 8.18 | 2011 | 2012 | |
| THAILAND | 1978 | 4.97 | 2013 | 2015 | |
| PERU | 1978 | 4.33 | 2016 | 2017 | |
| JORDAN | 1978 | 6.41 | 2017 | 2022 | |
| PHILIPPINES | 1978 | 4.46 | 2017 | 2022 | |
| COLOMBIA | 1978 | 4.43 | 2017 | 2018 | |
| KOSOVO | 1978 | 6.41 | 2018 | 2019 | |
| IRAQ | 1978 | 4.94 | 2018 | 2022 | |
| IRAN | 1978 | 6.93 | 2019 | 2020 | |
| ETHIOPIA | 1978 | 6.9 | 2019 | 2022 | |
| CYPRUS | 1978 | 6.47 | 2019 | 2022 | |
| PALESTINE | 1978 | 6.12 | 2019 | 2022 | |
| VIETNAM | 1978 | 5.62 | 2019 | 2020 | |
| QATAR | 1978 | 5.47 | 2019 | 2022 | |
| PEOPLES R CHINA | 1978 | 149.84 | 2020 | 2022 | |
| PAKISTAN | 1978 | 11.73 | 2020 | 2022 | |
| MALAYSIA | 1978 | 9.53 | 2020 | 2022 | |
| BANGLADESH | 1978 | 6.76 | 2020 | 2022 | |
| NEPAL | 1978 | 4.39 | 2020 | 2022 | |

**B. Top 30 countries sorted by strength of citation bursts (1978-2022)**

| Countries | Year | Strength | Begin | End | 1978 - 2022 |
|---|---|---|---|---|---|
| PEOPLES R CHINA | 1978 | 149.84 | 2020 | 2022 | |
| USA | 1978 | 57.25 | 1981 | 1993 | |
| CROATIA | 1978 | 20.06 | 1999 | 2012 | |
| BOSNIA & HERCEG | 1978 | 13.41 | 2006 | 2013 | |
| SERBIA | 1978 | 12.04 | 2009 | 2013 | |
| PAKISTAN | 1978 | 11.73 | 2020 | 2022 | |
| ISRAEL | 1978 | 11.05 | 1986 | 2002 | |
| MALAYSIA | 1978 | 9.53 | 2020 | 2022 | |
| BRAZIL | 1978 | 8.18 | 2011 | 2012 | |
| IRAN | 1978 | 6.93 | 2019 | 2020 | |
| ETHIOPIA | 1978 | 6.9 | 2019 | 2022 | |
| BANGLADESH | 1978 | 6.76 | 2020 | 2022 | |
| CYPRUS | 1978 | 6.47 | 2019 | 2022 | |
| KOSOVO | 1978 | 6.41 | 2018 | 2019 | |
| JORDAN | 1978 | 6.41 | 2017 | 2022 | |
| PALESTINE | 1978 | 6.12 | 2019 | 2022 | |
| VIETNAM | 1978 | 5.62 | 2019 | 2020 | |
| QATAR | 1978 | 5.47 | 2019 | 2022 | |
| RUSSIA | 1978 | 5.33 | 1991 | 2006 | |
| THAILAND | 1978 | 4.97 | 2013 | 2015 | |
| IRAQ | 1978 | 4.94 | 2018 | 2022 | |
| PHILIPPINES | 1978 | 4.46 | 2017 | 2022 | |
| COLOMBIA | 1978 | 4.43 | 2017 | 2018 | |
| NEPAL | 1978 | 4.39 | 2020 | 2022 | |
| PERU | 1978 | 4.33 | 2016 | 2017 | |

## C. Top 30 institutions sorted by the beginning year of citation burst (2020-2022)

| Institutions | Year | Strength | Begin | End | 2000 - 2022 |
|---|---|---|---|---|---|
| Mt Sinai Sch Med | 2000 | 95.88 | 2000 | 2011 | |
| Med Univ S Carolina | 2000 | 64.4 | 2000 | 2015 | |
| Vet Affairs Med Ctr | 2000 | 43.5 | 2000 | 2008 | |
| Univ Texas | 2000 | 43.49 | 2000 | 2007 | |
| Univ Calif Los Angeles | 2000 | 28.75 | 2000 | 2010 | |
| NIMH | 2000 | 26.87 | 2000 | 2008 | |
| Duke Univ | 2000 | 26.34 | 2000 | 2008 | |
| Univ Zagreb | 2000 | 19.43 | 2000 | 2010 | |
| Univ Stellenbosch | 2000 | 17.97 | 2000 | 2009 | |
| Univ Med & Dent New Jersey | 2000 | 26.49 | 2001 | 2013 | |
| CUNY Mt Sinai Sch Med | 2000 | 21.99 | 2001 | 2007 | |
| Univ Munich | 2000 | 21.91 | 2001 | 2013 | |
| VA Med Ctr | 2000 | 20.05 | 2001 | 2008 | |
| Inst Psychiat | 2000 | 17.05 | 2001 | 2010 | |
| New York Acad Med | 2000 | 23.21 | 2002 | 2008 | |
| USA | 2000 | 20.04 | 2004 | 2012 | |
| Cent Inst Mental Hlth | 2000 | 19.11 | 2004 | 2012 | |
| Univ New S Wales | 2000 | 56.84 | 2005 | 2015 | |
| Univ Texas SW Med Ctr Dallas | 2000 | 27.94 | 2008 | 2015 | |
| USN | 2000 | 27.9 | 2008 | 2013 | |
| Univ So Calif | 2000 | 28.71 | 2009 | 2016 | |
| Howard Hughes Med Inst | 2000 | 26.24 | 2009 | 2015 | |
| Univ Ulster | 2000 | 19.1 | 2011 | 2017 | |
| Harvard TH Chan Sch Publ Hlth | 2000 | 27.11 | 2017 | 2022 | |
| Med Univ South Carolina | 2000 | 23.69 | 2017 | 2022 | |

## D. Top 30 institutions sorted by strength of citation burst (2020-2022)

| Institutions | Year | Strength | Begin | End | 2000 - 2022 |
|---|---|---|---|---|---|
| Mt Sinai Sch Med | 2000 | 95.88 | 2000 | 2011 | |
| Med Univ S Carolina | 2000 | 64.4 | 2000 | 2015 | |
| Univ New S Wales | 2000 | 56.84 | 2005 | 2015 | |
| Vet Affairs Med Ctr | 2000 | 43.5 | 2000 | 2008 | |
| Univ Texas | 2000 | 43.49 | 2000 | 2007 | |
| Univ Calif Los Angeles | 2000 | 28.75 | 2000 | 2010 | |
| Univ So Calif | 2000 | 28.71 | 2009 | 2016 | |
| Univ Texas SW Med Ctr Dallas | 2000 | 27.94 | 2008 | 2015 | |
| USN | 2000 | 27.9 | 2008 | 2013 | |
| Harvard TH Chan Sch Publ Hlth | 2000 | 27.11 | 2017 | 2022 | |
| NIMH | 2000 | 26.87 | 2000 | 2008 | |
| Univ Med & Dent New Jersey | 2000 | 26.49 | 2001 | 2013 | |
| Duke Univ | 2000 | 26.34 | 2000 | 2008 | |
| Howard Hughes Med Inst | 2000 | 26.24 | 2009 | 2015 | |
| Med Univ South Carolina | 2000 | 23.69 | 2017 | 2022 | |
| New York Acad Med | 2000 | 23.21 | 2002 | 2008 | |
| CUNY Mt Sinai Sch Med | 2000 | 21.99 | 2001 | 2007 | |
| Univ Munich | 2000 | 21.91 | 2001 | 2013 | |
| VA Med Ctr | 2000 | 20.05 | 2001 | 2008 | |
| USA | 2000 | 20.04 | 2004 | 2012 | |
| Univ Zagreb | 2000 | 19.43 | 2000 | 2010 | |
| Cent Inst Mental Hlth | 2000 | 19.11 | 2004 | 2012 | |
| Univ Ulster | 2000 | 19.1 | 2011 | 2017 | |
| Univ Stellenbosch | 2000 | 17.97 | 2000 | 2009 | |
| Inst Psychiat | 2000 | 17.05 | 2001 | 2010 | |

**E. Top 25 journals sorted by beginning year of citation burst (2000-2022)**

| Cited Journals | Year | Strength | Begin | End | 2000 - 2022 |
|---|---|---|---|---|---|
| AM J PSYCHIAT | 2000 | 648.02 | 2000 | 2008 | |
| J NERV MENT DIS | 2000 | 381.59 | 2000 | 2008 | |
| DIAGN STAT MAN MENT | 2000 | 300.99 | 2000 | 2008 | |
| ARCH GEN PSYCHIAT | 2000 | 231.56 | 2000 | 2006 | |
| BRIT J PSYCHIAT | 2000 | 216.53 | 2000 | 2006 | |
| PSYCHOSOM MED | 2000 | 203.53 | 2000 | 2008 | |
| PSYCHIAT CLIN N AM | 2000 | 202.7 | 2000 | 2010 | |
| J CONSULT CLIN PSYCH | 2000 | 192.07 | 2000 | 2006 | |
| BRIT MED J | 2000 | 190.97 | 2000 | 2009 | |
| J PSYCHIAT RES | 2000 | 170.7 | 2000 | 2011 | |
| STRUCTURED CLIN INTE | 2000 | 167.64 | 2000 | 2011 | |
| PSYCHIATR SERV | 2000 | 160.82 | 2000 | 2012 | |
| PSYCHOPHARMACOL BULL | 2000 | 149.15 | 2000 | 2010 | |
| SCI REP-UK | 2000 | 382.8 | 2019 | 2022 | |
| FRONT PSYCHOL | 2000 | 367.41 | 2019 | 2022 | |
| EUR J PSYCHOTRAUMATO | 2000 | 261.42 | 2019 | 2022 | |
| BMJ OPEN | 2000 | 227.74 | 2019 | 2022 | |
| NAT COMMUN | 2000 | 185.75 | 2019 | 2022 | |
| INT J MOL SCI | 2000 | 157.65 | 2019 | 2022 | |
| TRANSL PSYCHIAT | 2000 | 157.1 | 2019 | 2022 | |
| CURR PSYCHIAT REP | 2000 | 149.36 | 2019 | 2022 | |
| INT J ENV RES PUB HE | 2000 | 518.62 | 2020 | 2022 | |
| LANCET PSYCHIAT | 2000 | 443.06 | 2020 | 2022 | |
| FRONT PSYCHIATRY | 2000 | 438.88 | 2020 | 2022 | |
| JAMA NETW OPEN | 2000 | 304.01 | 2020 | 2022 | |

## F. Top 25 journals sorted by strength of citation burst (1920-2022)

| Cited Journals | Year | Strength | Begin | End | 2000 - 2022 |
|---|---|---|---|---|---|
| AM J PSYCHIAT | 2000 | 648.02 | 2000 | 2008 | |
| INT J ENV RES PUB HE | 2000 | 518.62 | 2020 | 2022 | |
| LANCET PSYCHIAT | 2000 | 443.06 | 2020 | 2022 | |
| FRONT PSYCHIATRY | 2000 | 438.88 | 2020 | 2022 | |
| SCI REP-UK | 2000 | 382.8 | 2019 | 2022 | |
| J NERV MENT DIS | 2000 | 381.59 | 2000 | 2008 | |
| FRONT PSYCHOL | 2000 | 367.41 | 2019 | 2022 | |
| JAMA NETW OPEN | 2000 | 304.01 | 2020 | 2022 | |
| DIAGN STAT MAN MENT | 2000 | 300.99 | 2000 | 2008 | |
| EUR J PSYCHOTRAUMATO | 2000 | 261.42 | 2019 | 2022 | |
| ARCH GEN PSYCHIAT | 2000 | 231.56 | 2000 | 2006 | |
| BMJ OPEN | 2000 | 227.74 | 2019 | 2022 | |
| BRIT J PSYCHIAT | 2000 | 216.53 | 2000 | 2006 | |
| PSYCHOSOM MED | 2000 | 203.53 | 2000 | 2008 | |
| PSYCHIAT CLIN N AM | 2000 | 202.7 | 2000 | 2010 | |
| J CONSULT CLIN PSYCH | 2000 | 192.07 | 2000 | 2006 | |
| BRIT MED J | 2000 | 190.97 | 2000 | 2009 | |
| NAT COMMUN | 2000 | 185.75 | 2019 | 2022 | |
| J PSYCHIAT RES | 2000 | 170.7 | 2000 | 2011 | |
| STRUCTURED CLIN INTE | 2000 | 167.64 | 2000 | 2011 | |
| PSYCHIATR SERV | 2000 | 160.82 | 2000 | 2012 | |
| INT J MOL SCI | 2000 | 157.65 | 2019 | 2022 | |
| TRANSL PSYCHIAT | 2000 | 157.1 | 2019 | 2022 | |
| CURR PSYCHIAT REP | 2000 | 149.36 | 2019 | 2022 | |
| PSYCHOPHARMACOL BULL | 2000 | 149.15 | 2000 | 2010 | |

**G. Top 25 co-authors sorted by year of citation burst (2000-2022)**

| Authors | Year | Strength | Begin | End | 2000 - 2022 |
|---|---|---|---|---|---|
| DAVIDSON J | 2000 | 34.53 | 2000 | 2008 | |
| YEHUDA R | 2000 | 32.04 | 2000 | 2007 | |
| CHARNEY D | 2000 | 25.81 | 2000 | 2010 | |
| PITMAN R | 2000 | 25.05 | 2000 | 2012 | |
| BREMNER J | 2000 | 25.01 | 2000 | 2010 | |
| FRUEH B | 2000 | 19.87 | 2000 | 2013 | |
| ROSENHECK R | 2000 | 17.73 | 2000 | 2014 | |
| KILPATRICK D | 2000 | 14.23 | 2000 | 2012 | |
| ORR S | 2000 | 12.45 | 2000 | 2009 | |
| NEMEROFF C | 2000 | 11.71 | 2000 | 2005 | |
| BRADY K | 2000 | 11.7 | 2000 | 2009 | |
| CREAMER M | 2000 | 13.59 | 2001 | 2011 | |
| CONNOR K | 2000 | 12.04 | 2001 | 2008 | |
| STEIN M | 2000 | 16.97 | 2002 | 2010 | |
| VERMETTEN E | 2000 | 16.86 | 2002 | 2013 | |
| WESSELY S | 2000 | 20.94 | 2003 | 2013 | |
| GALEA S | 2000 | 14.04 | 2004 | 2015 | |
| FEAR N | 2000 | 14.32 | 2007 | 2013 | |
| SMITH T | 2000 | 12.31 | 2007 | 2013 | |
| HOTOPF M | 2000 | 12.23 | 2007 | 2011 | |
| BREWIN C | 2000 | 11.96 | 2007 | 2011 | |
| FIGUEIRA I | 2000 | 11.8 | 2008 | 2015 | |
| BRADLEY B | 2000 | 19.57 | 2009 | 2016 | |
| PARK J | 2000 | 11.85 | 2016 | 2022 | |
| KIM H | 2000 | 13.47 | 2017 | 2022 | |

**H. Top 25 co-authors sorted by strength year of citation burst (2000-2022)**

| Authors | Year | Strength | Begin | End | 2000 - 2022 |
|---|---|---|---|---|---|
| DAVIDSON J | 2000 | 34.53 | 2000 | 2008 | |
| YEHUDA R | 2000 | 32.04 | 2000 | 2007 | |
| CHARNEY D | 2000 | 25.81 | 2000 | 2010 | |
| PITMAN R | 2000 | 25.05 | 2000 | 2012 | |
| BREMNER J | 2000 | 25.01 | 2000 | 2010 | |
| WESSELY S | 2000 | 20.94 | 2003 | 2013 | |
| FRUEH B | 2000 | 19.87 | 2000 | 2013 | |
| BRADLEY B | 2000 | 19.57 | 2009 | 2016 | |
| ROSENHECK R | 2000 | 17.73 | 2000 | 2014 | |
| STEIN M | 2000 | 16.97 | 2002 | 2010 | |
| VERMETTEN E | 2000 | 16.86 | 2002 | 2013 | |
| FEAR N | 2000 | 14.32 | 2007 | 2013 | |
| KILPATRICK D | 2000 | 14.23 | 2000 | 2012 | |
| GALEA S | 2000 | 14.04 | 2004 | 2015 | |
| CREAMER M | 2000 | 13.59 | 2001 | 2011 | |
| KIM H | 2000 | 13.47 | 2017 | 2022 | |
| ORR S | 2000 | 12.45 | 2000 | 2009 | |
| SMITH T | 2000 | 12.31 | 2007 | 2013 | |
| HOTOPF M | 2000 | 12.23 | 2007 | 2011 | |
| CONNOR K | 2000 | 12.04 | 2001 | 2008 | |
| BREWIN C | 2000 | 11.96 | 2007 | 2011 | |
| PARK J | 2000 | 11.85 | 2016 | 2022 | |
| FIGUEIRA I | 2000 | 11.8 | 2008 | 2015 | |
| NEMEROFF C | 2000 | 11.71 | 2000 | 2005 | |
| BRADY K | 2000 | 11.7 | 2000 | 2009 | |

**I. Top 30 co-occurrence keywords sorted by beginning year of citation burst (2017-2022)**

| Keywords | Year | Strength | Begin | End | 2017 - 2022 |
|---|---|---|---|---|---|
| national comorbidity survey | 2017 | 17.72 | 2017 | 2018 | □□□□□□ |
| randomized clinical trial | 2017 | 10.7 | 2017 | 2018 | □□□□□□ |
| confirmatory factor analysis | 2017 | 9.23 | 2017 | 2018 | □□□□□□ |
| administered ptsd scale | 2017 | 8.12 | 2017 | 2018 | □□□□□□ |
| dsm iv disorder | 2017 | 7.97 | 2017 | 2018 | □□□□□□ |
| social anxiety disorder | 2017 | 7.79 | 2017 | 2018 | □□□□□□ |
| anxietydisorder | 2017 | 7.74 | 2017 | 2018 | □□□□□□ |
| preterm birth | 2017 | 7.38 | 2017 | 2018 | □□□□□□ |
| new york city | 2017 | 7.38 | 2017 | 2018 | □□□□□□ |
| comorbidity survey replication | 2017 | 7.38 | 2017 | 2018 | □□□□□□ |
| psychometric evaluation | 2017 | 7.01 | 2017 | 2018 | □□□□□□ |
| women veteran | 2017 | 6.63 | 2017 | 2018 | □□□□□□ |
| german version | 2017 | 6.27 | 2017 | 2018 | □□□□□□ |
| acute psychosocial stress | 2017 | 6.27 | 2017 | 2018 | □□□□□□ |
| community sample | 2017 | 6.26 | 2017 | 2018 | □□□□□□ |
| young children | 2017 | 6.22 | 2017 | 2018 | □□□□□□ |
| critically ill patient | 2017 | 6.22 | 2017 | 2018 | □□□□□□ |
| prolonged grief | 2017 | 8.38 | 2019 | 2020 | □□□□□□ |
| stria terminali | 2017 | 6.77 | 2019 | 2020 | □□□□□□ |
| telemedicine | 2017 | 6.44 | 2019 | 2020 | □□□□□□ |
| psychological impact | 2017 | 30.62 | 2020 | 2022 | □□□□□□ |
| acute respiratory syndrome | 2017 | 27.03 | 2020 | 2022 | □□□□□□ |
| sar | 2017 | 23.6 | 2020 | 2022 | □□□□□□ |
| quarantine | 2017 | 11.25 | 2020 | 2022 | □□□□□□ |
| immune system | 2017 | 7.59 | 2020 | 2022 | □□□□□□ |
| hospital worker | 2017 | 7.22 | 2020 | 2022 | □□□□□□ |
| phq 9 | 2017 | 6.25 | 2020 | 2022 | □□□□□□ |
| toronto | 2017 | 6.18 | 2020 | 2022 | □□□□□□ |
| avoidance | 2017 | 6.18 | 2020 | 2022 | □□□□□□ |
| prediction | 2017 | 6.18 | 2020 | 2022 | □□▂□□□ |

**J. Top 30 co-occurrence keywords sorted by strength year of citation burst (2017-2022)**

| Keywords | Year | Strength | Begin | End | 2017 - 2022 |
|---|---|---|---|---|---|
| psychological impact | 2017 | 30.62 | 2020 | 2022 | □□□□□□ |
| acute respiratory syndrome | 2017 | 27.03 | 2020 | 2022 | □□□□□□ |
| sar | 2017 | 23.6 | 2020 | 2022 | □□□□□□ |
| national comorbidity survey | 2017 | 17.72 | 2017 | 2018 | □□□□□□ |
| quarantine | 2017 | 11.25 | 2020 | 2022 | □□□□□□ |
| randomized clinical trial | 2017 | 10.7 | 2017 | 2018 | □□□□□□ |
| confirmatory factor analysis | 2017 | 9.23 | 2017 | 2018 | □□□□□□ |
| prolonged grief | 2017 | 8.38 | 2019 | 2020 | □□□□□□ |
| administered ptsd scale | 2017 | 8.12 | 2017 | 2018 | □□□□□□ |
| dsm iv disorder | 2017 | 7.97 | 2017 | 2018 | □□□□□□ |
| social anxiety disorder | 2017 | 7.79 | 2017 | 2018 | □□□□□□ |
| anxietydisorder | 2017 | 7.74 | 2017 | 2018 | □□□□□□ |
| immune system | 2017 | 7.59 | 2020 | 2022 | □□□□□□ |
| preterm birth | 2017 | 7.38 | 2017 | 2018 | □□□□□□ |
| new york city | 2017 | 7.38 | 2017 | 2018 | □□□□□□ |
| comorbidity survey replication | 2017 | 7.38 | 2017 | 2018 | □□□□□□ |
| hospital worker | 2017 | 7.22 | 2020 | 2022 | □□□□□□ |
| psychometric evaluation | 2017 | 7.01 | 2017 | 2018 | □□□□□□ |
| stria terminali | 2017 | 6.77 | 2019 | 2020 | □□□□□□ |
| women veteran | 2017 | 6.63 | 2017 | 2018 | □□□□□□ |
| telemedicine | 2017 | 6.44 | 2019 | 2020 | □□□□□□ |
| german version | 2017 | 6.27 | 2017 | 2018 | □□□□□□ |
| acute psychosocial stress | 2017 | 6.27 | 2017 | 2018 | □□□□□□ |
| community sample | 2017 | 6.26 | 2017 | 2018 | □□□□□□ |
| phq 9 | 2017 | 6.25 | 2020 | 2022 | □□□□□□ |
| young children | 2017 | 6.22 | 2017 | 2018 | □□□□□□ |
| critically ill patient | 2017 | 6.22 | 2017 | 2018 | □□□□□□ |
| toronto | 2017 | 6.18 | 2020 | 2022 | □□□□□□ |
| avoidance | 2017 | 6.18 | 2020 | 2022 | □□□□□□ |
| prediction | 2017 | 6.18 | 2020 | 2022 | □□□□□□ |

**K. Top 25 references with strongest beginning year of citation busts (1978-2022)**

| References | Year | Strength | Begin | End | 1978 - 2022 |
|---|---|---|---|---|---|
| Kessler RC, 1995, ARCH GEN PSYCHIAT, V52, P1048, DOI 10.1001/archpsyc.1995.03950240066012, DOI | 1995 | 117.54 | 1996 | 2000 | |
| Bremner JD, 1995, AM J PSYCHIAT, V152, P973 | 1995 | 60.54 | 1996 | 2000 | |
| Breslau N, 1998, ARCH GEN PSYCHIAT, V55, P626, DOI 10.1001/archpsyc.55.7.626, DOI | 1998 | 66.54 | 1999 | 2003 | |
| Galea S, 2002, NEW ENGL J MED, V346, P982, DOI 10.1056/NEJMsa013404, DOI | 2002 | 76.67 | 2002 | 2007 | |
| Yehuda R, 2002, NEW ENGL J MED, V346, P108, DOI 10.1056/NEJMra012941, DOI | 2002 | 63.59 | 2002 | 2007 | |
| Gilbertson MW, 2002, NAT NEUROSCI, V5, P1242, DOI 10.1038/nn958, DOI | 2002 | 65.52 | 2003 | 2007 | |
| Hoge CW, 2004, NEW ENGL J MED, V351, P13, DOI 10.1056/NEJMoa040603, DOI | 2004 | 100.43 | 2005 | 2009 | |
| Kessler RC, 2005, ARCH GEN PSYCHIAT, V62, P593, DOI 10.1001/archpsyc.62.6.593, DOI | 2005 | 111.3 | 2006 | 2010 | |
| Hoge CW, 2006, JAMA-J AM MED ASSOC, V295, P1023, DOI 10.1001/jama.295.9.1023, DOI | 2006 | 77.43 | 2007 | 2011 | |
| Milliken CS, 2007, JAMA-J AM MED ASSOC, V298, P2141, DOI 10.1001/jama.298.18.2141, DOI | 2007 | 76.6 | 2008 | 2012 | |
| Cohen J, 2013, STAT POWER ANAL BEHA, V2nd, P0, DOI 10.4324/978020377158, DOI | 2013 | 61.35 | 2013 | 2018 | |
| Binder EB, 2008, JAMA-J AM MED ASSOC, V299, P1291, DOI 10.1001/jama.299.11.1291, DOI | 2008 | 75.32 | 2009 | 2013 | |
| Milad MR, 2009, BIOL PSYCHIAT, V66, P1075, DOI 10.1016/j.biopsych.2009.06.026, DOI | 2009 | 65.58 | 2010 | 2014 | |
| Shin LM, 2010, NEUROPSYCHOPHARMACOL, V35, P169, DOI 10.1038/npp.2009.83, DOI | 2010 | 64.19 | 2010 | 2015 | |
| Thomas JL, 2010, ARCH GEN PSYCHIAT, V67, P614, DOI 10.1001/archgenpsychiatry.2010.54, DOI | 2010 | 60.86 | 2011 | 2015 | |
| Pitman RK, 2012, NAT REV NEUROSCI, V13, P769, DOI 10.1038/nrn3339, DOI | 2012 | 97.41 | 2014 | 2017 | |
| Weathers FW, 2013, PTSD CHECKLIST DSM 5, V0, P0 | 2013 | 88.39 | 2015 | 2018 | |
| Hayes AF, 2018, METHODOLOGY SOCIAL S, V0, P0 | 2018 | 60.97 | 2018 | 2020 | |
| Blevins CA, 2015, J TRAUMA STRESS, V28, P489, DOI 10.1002/jts.22059, DOI | 2015 | 145.17 | 2017 | 2020 | |
| Passos IC, 2015, LANCET PSYCHIAT, V2, P1002, DOI 10.1016/S2215-0366(15)00309-0, DOI | 2015 | 60.45 | 2017 | 2020 | |
| Bovin MJ, 2016, PSYCHOL ASSESSMENT, V28, P1379, DOI 10.1037/pas0000254, DOI | 2016 | 100.89 | 2018 | 2022 | |
| Weathers FW, 2018, PSYCHOL ASSESSMENT, V30, P383, DOI 10.1037/pas0000486, DOI | 2018 | 71.87 | 2019 | 2022 | |
| Kessler RC, 2017, EUR J PSYCHOTRAUMATO, V8, P0, DOI 10.1080/20008198.2017.1353383, DOI | 2017 | 61.87 | 2019 | 2022 | |
| Lai JB, 2020, JAMA NETW OPEN, V3, P0, DOI 10.1001/jamanetworkopen.2020.3976, DOI | 2020 | 135.66 | 2020 | 2022 | |
| Brooks SK, 2020, LANCET, V395, P912 | 2020 | 100.2 | 2020 | 2022 | |

## L. Top 25 references sorted by strenghts of burst (1978-2022)

| References | Year | Strength | Begin | End | 1978 - 2022 |
|---|---|---|---|---|---|
| Blevins CA, 2015, J TRAUMA STRESS, V28, P489, DOI 10.1002/jts.22059, DOI | 2015 | 145.17 | 2017 | 2020 | |
| Lai JB, 2020, JAMA NETW OPEN, V3, P0, DOI 10.1001/jamanetworkopen.2020.3976, DOI | 2020 | 135.66 | 2020 | 2022 | |
| Kessler RC, 1995, ARCH GEN PSYCHIAT, V52, P1048, DOI 10.1001/archpsyc.1995.03950240066012, DOI | 1995 | 117.54 | 1996 | 2000 | |
| Kessler RC, 2005, ARCH GEN PSYCHIAT, V62, P593, DOI 10.1001/archpsyc.62.6.593, DOI | 2005 | 111.3 | 2006 | 2010 | |
| Bovin MJ, 2016, PSYCHOL ASSESSMENT, V28, P1379, DOI 10.1037/pas0000254, DOI | 2016 | 100.89 | 2018 | 2022 | |
| Hoge CW, 2004, NEW ENGL J MED, V351, P13, DOI 10.1056/NEJMoa040603, DOI | 2004 | 100.43 | 2005 | 2009 | |
| Brooks SK, 2020, LANCET, V395, P912 | 2020 | 100.2 | 2020 | 2022 | |
| Pitman RK, 2012, NAT REV NEUROSCI, V13, P769, DOI 10.1038/nrn3339, DOI | 2012 | 97.41 | 2014 | 2017 | |
| Weathers FW, 2013, PTSD CHECKLIST DSM 5, V0, P0 | 2013 | 88.39 | 2015 | 2018 | |
| Wang CY, 2020, INT J ENV RES PUB HE, V17, P0, DOI 10.3390/ijerph17051729, DOI | 2020 | 79.72 | 2020 | 2022 | |
| Hoge CW, 2006, JAMA-J AM MED ASSOC, V295, P1023, DOI 10.1001/jama.295.9.1023, DOI | 2006 | 77.43 | 2007 | 2011 | |
| Galea S, 2002, NEW ENGL J MED, V346, P982, DOI 10.1056/NEJMsa013404, DOI | 2002 | 76.67 | 2002 | 2007 | |
| Milliken CS, 2007, JAMA-J AM MED ASSOC, V298, P2141, DOI 10.1001/jama.298.18.2141, DOI | 2007 | 76.6 | 2008 | 2012 | |
| Binder EB, 2008, JAMA-J AM MED ASSOC, V299, P1291, DOI 10.1001/jama.299.11.1291, DOI | 2008 | 75.32 | 2009 | 2013 | |
| Weathers FW, 2018, PSYCHOL ASSESSMENT, V30, P383, DOI 10.1037/pas0000486, DOI | 2018 | 71.87 | 2019 | 2022 | |
| [anonymous] , 2013, DIAGNOSTIC STAT MANU, V17, P133, DOI 10.1176/appi.books.9780890425596, DOI | 2013 | 67.91 | 2014 | 2018 | |
| Breslau N, 1998, ARCH GEN PSYCHIAT, V55, P626, DOI 10.1001/archpsyc.55.7.626, DOI | 1998 | 66.54 | 1999 | 2003 | |
| Milad MR, 2009, BIOL PSYCHIAT, V66, P1075, DOI 10.1016/j.biopsych.2009.06.026, DOI | 2009 | 65.58 | 2010 | 2014 | |
| Gilbertson MW, 2002, NAT NEUROSCI, V5, P1242, DOI 10.1038/nn958, DOI | 2002 | 65.52 | 2003 | 2007 | |
| Pappa S, 2020, BRAIN BEHAV IMMUN, V88, P901 | 2020 | 64.38 | 2020 | 2022 | |
| Shin LM, 2010, NEUROPSYCHOPHARMACOL, V35, P169, DOI 10.1038/npp.2009.83, DOI | 2010 | 64.19 | 2010 | 2015 | |
| Yehuda R, 2002, NEW ENGL J MED, V346, P108, DOI 10.1056/NEJMra012941, DOI | 2002 | 63.59 | 2002 | 2007 | |
| Kessler RC, 2017, EUR J PSYCHOTRAUMATO, V8, P0, DOI 10.1080/20008198.2017.1353383, DOI | 2017 | 61.87 | 2019 | 2022 | |
| Cohen J, 2013, STAT POWER ANAL BEHA, V2nd, P0, DOI 10.4324/978020377158, DOI | 2013 | 61.35 | 2013 | 2018 | |
| Hayes AF, 2018, METHODOLOGY SOCIAL S, V0, P0 | 2018 | 60.97 | 2018 | 2020 | |

**M. Top 25 cited references with strongest beginning of citation burst (2020-2022)**

| References | Year | Strength | Begin | End | 2020-JAN - 2022-JUN |
|---|---|---|---|---|---|
| Blevins C, 2015, J TRAUMA STRESS, V28, P489, DOI 10.1002/jts.22059, DOI | 2015-JAN | 39.4 | 2020-JAN | 2020-DEC | □□□□□□□□□□□□□□□□□□□□□□□□□□□□□□□□ |
| Shalev A, 2017, NEW ENGL J MED, V376, P2459, DOI 10.1056/NEJMra1612499, DOI | 2017-JAN | 11.03 | 2020-JAN | 2020-JUN | □□□□□□□□□□□□□□□□□□□□□□□□□□□□□□□□ |
| Yehuda R, 2015, NAT REV DIS PRIMERS, V1, P0, DOI 10.1038/nrdp.2015.57, DOI | 2015-JAN | 7.88 | 2020-JAN | 2020-NOV | □□□□□□□□□□□□□□□□□□□□□□□□□□□□□□□□ |
| Liberzon I, 2016, NEURON, V92, P14, DOI 10.1016/j.neuron.2016.09.039, DOI | 2016-JAN | 6.85 | 2020-JAN | 2020-OCT | □□□□□□□□□□□□□□□□□□□□□□□□□□□□□□□□ |
| Duncan L, 2018, MOL PSYCHIATR, V23, P666, DOI 10.1038/mp.2017.77, DOI | 2018-JAN | 6.56 | 2020-JAN | 2020-APR | □□□□□□□□□□□□□□□□□□□□□□□□□□□□□□□□ |
| Bogic M, 2015, BMC INT HEALTH HUM R, V15, P0, DOI 10.1186/s12914-015-0064-9, DOI | 2015-JAN | 13.04 | 2020-FEB | 2020-OCT | □□□□□□□□□□□□□□□□□□□□□□□□□□□□□□□□ |
| Parker A, 2015, CRIT CARE MED, V43, P1121, DOI 10.1097/CCM.0000000000000882, DOI | 2015-JAN | 7.18 | 2020-FEB | 2020-NOV | □□□□□□□□□□□□□□□□□□□□□□□□□□□□□□□□ |
| Li Z, 2020, BRAIN BEHAV IMMUN, V88, P916, DOI 10.1016/j.bbi.2020.03.007, DOI | 2020-JAN | 7.83 | 2020-JUN | 2020-DEC | □□□□□□□□□□□□□□□□□□□□□□□□□□□□□□□□ |
| Liu S, 2020, LANCET PSYCHIAT, V7, P0, DOI 10.1016/S2215-0366(20)30077-8, DOI | 2020-JAN | 7.32 | 2020-AUG | 2020-DEC | □□□□□□□□□□□□□□□□□□□□□□□□□□□□□□□□ |
| Wang C, 2020, INT J ENV RES PUB HE, V17, P0, DOI 10.3390/ijerph17072459, DOI | 2020-JAN | 6.94 | 2020-SEP | 2021-JAN | □□□□□□□□□□□□□□□□□□□□□□□□□□□□□□□□ |
| Kang L, 2020, BRAIN BEHAV IMMUN, V87, P11, DOI 10.1016/j.bbi.2020.03.028, DOI | 2020-JAN | 7.71 | 2020-OCT | 2021-JAN | □□□□□□□□□□□□□□□□□□□□□□□□□□□□□□□□ |
| Tang W, 2020, J AFFECT DISORDERS, V274, P1, DOI 10.1016/j.jad.2020.05.009, DOI | 2020-JAN | 6.96 | 2020-NOV | 2021-JUN | □□□□□□□□□□□□□□□□□□□□□□□□□□□□□□□□ |
| Wang C, 2020, LANCET, V395, P470, DOI 10.1016/S0140-6736(20)30185-9, DOI | 2020-JAN | 6.68 | 2020-DEC | 2021-FEB | □□□□□□□□□□□□□□□□□□□□□□□□□□□□□□□□ |
| Rossi R, 2020, JAMA NETW OPEN, V3, P0, DOI 10.1001/jamanetworkopen.2020.10185, DOI | 2020-JAN | 7.7 | 2021-JAN | 2022-JAN | □□□□□□□□□□□□□□□□□□□□□□□□□□□□□□□□ |
| Tan B, 2020, ANN INTERN MED, V173, P317, DOI 10.7326/M20-1083, DOI | 2020-JAN | 6.65 | 2021-JAN | 2021-MAR | □□□□□□□□□□□□□□□□□□□□□□□□□□□□□□□□ |
| Luo M, 2020, PSYCHIAT RES, V291, P0, DOI 10.1016/j.psychres.2020.113190, DOI | 2020-JAN | 7.39 | 2021-FEB | 2021-JUN | □□□□□□□□□□□□□□□□□□□□□□□□□□□□□□□□ |
| Pfefferbaum B, 2020, NEW ENGL J MED, V383, P510, DOI 10.1056/NEJMp2008017, DOI | 2020-JAN | 7.39 | 2021-FEB | 2021-JUN | □□□□□□□□□□□□□□□□□□□□□□□□□□□□□□□□ |
| Yang Y, 2020, LANCET PSYCHIAT, V7, P0, DOI 10.1016/S2215-0366(20)30079-1, DOI | 2020-JAN | 7.12 | 2021-MAY | 2021-SEP | □□□□□□□□□□□□□□□□□□□□□□□□□□□□□□□□ |
| Pappa S, 2020, BRAIN BEHAV IMMUN, V88, P901 | 2020-JAN | 11.57 | 2021-JUN | 2022-APR | □□□□□□□□□□□□□□□□□□□□□□□□□□□□□□□□ |
| Carmassi C, 2020, PSYCHIAT RES, V292, P0, DOI 10.1016/j.psychres.2020.113312, DOI | 2020-JAN | 8.54 | 2021-JUN | 2022-APR | □□□□□□□□□□□□□□□□□□□□□□□□□□□□□□□□ |
| R D, 2019, R LANGUAGE ENV STAT, V0, P0 | 2019-JAN | 7.92 | 2021-JUN | 2022-MAR | □□□□□□□□□□□□□□□□□□□□□□□□□□□□□□□□ |
| De P, 2020, J AFFECT DISORDERS, V275, P48, DOI 10.1016/j.jad.2020.06.022, DOI | 2020-JAN | 6.69 | 2021-JUN | 2022-APR | □□□□□□□□□□□□□□□□□□□□□□□□□□□□□□□□ |
| Cloitre M, 2018, ACTA PSYCHIAT SCAND, V138, P536, DOI 10.1111/acps.12956, DOI | 2018-JAN | 6.97 | 2021-OCT | 2022-MAY | □□□□□□□□□□□□□□□□□□□□□□□□□□□□□□□□ |
| Vindegaard N, 2020, BRAIN BEHAV IMMUN, V89, P531, DOI 10.1016/j.bbi.2020.05.048, DOI | 2020-JAN | 9.66 | 2021-DEC | 2022-MAR | □□□□□□□□□□□□□□□□□□□□□□□□□□□□□□□□ |
| Xiong J, 2020, J AFFECT DISORDERS, V277, P55, DOI 10.1016/j.jad.2020.08.001, DOI | 2020-JAN | 6.76 | 2022-MAR | 2022-JUN | □□□□□□□□□□□□□□□□□□□□□□□□□□□□□□□ |

**N. Top 25 references sorted by strengths of burst (2020-2022)**

| References | Year | Strength | Begin | End | 2020-JAN - 2022-JUN |
|---|---|---|---|---|---|
| Blevins C, 2015, J TRAUMA STRESS, V28, P489, DOI 10.1002/jts.22059, DOI | 2015-JAN | 39.4 | 2020-JAN | 2020-DEC | □□□□□□□□□□□□□□□□□□□□□□□□□□□□□□□□ |
| Bogic M, 2015, BMC INT HEALTH HUM R, V15, P0, DOI 10.1186/s12914-015-0064-9, DOI | 2015-JAN | 13.04 | 2020-FEB | 2020-OCT | □□□□□□□□□□□□□□□□□□□□□□□□□□□□□□□□ |
| Pappa S, 2020, BRAIN BEHAV IMMUN, V88, P901 | 2020-JAN | 11.57 | 2021-JUN | 2022-APR | □□□□□□□□□□□□□□□□□□□□□□□□□□□□□□□□ |
| Shalev A, 2017, NEW ENGL J MED, V376, P2459, DOI 10.1056/NEJMra1612499, DOI | 2017-JAN | 11.03 | 2020-JAN | 2020-JUN | □□□□□□□□□□□□□□□□□□□□□□□□□□□□□□□□ |
| Vindegaard N, 2020, BRAIN BEHAV IMMUN, V89, P531, DOI 10.1016/j.bbi.2020.05.048, DOI | 2020-JAN | 9.66 | 2021-DEC | 2022-MAR | □□□□□□□□□□□□□□□□□□□□□□□□□□□□□□□□ |
| Carmassi C, 2020, PSYCHIAT RES, V292, P0, DOI 10.1016/j.psychres.2020.113312, DOI | 2020-JAN | 8.54 | 2021-JUN | 2022-APR | □□□□□□□□□□□□□□□□□□□□□□□□□□□□□□□□ |
| R D, 2019, R LANGUAGE ENV STAT, V0, P0 | 2019-JAN | 7.92 | 2021-JUN | 2022-MAR | □□□□□□□□□□□□□□□□□□□□□□□□□□□□□□□□ |
| Yehuda R, 2015, NAT REV DIS PRIMERS, V1, P0, DOI 10.1038/nrdp.2015.57, DOI | 2015-JAN | 7.88 | 2020-JAN | 2020-NOV | □□□□□□□□□□□□□□□□□□□□□□□□□□□□□□□□ |
| Li Z, 2020, BRAIN BEHAV IMMUN, V88, P916, DOI 10.1016/j.bbi.2020.03.007, DOI | 2020-JAN | 7.83 | 2020-JUN | 2020-DEC | □□□□□□□□□□□□□□□□□□□□□□□□□□□□□□□□ |
| Kang L, 2020, BRAIN BEHAV IMMUN, V87, P11, DOI 10.1016/j.bbi.2020.03.028, DOI | 2020-JAN | 7.71 | 2020-OCT | 2021-JAN | □□□□□□□□□□□□□□□□□□□□□□□□□□□□□□□□ |
| Rossi R, 2020, JAMA NETW OPEN, V3, P0, DOI 10.1001/jamanetworkopen.2020.10185, DOI | 2020-JAN | 7.7 | 2021-JAN | 2022-JAN | □□□□□□□□□□□□□□□□□□□□□□□□□□□□□□□□ |
| Luo M, 2020, PSYCHIAT RES, V291, P0, DOI 10.1016/j.psychres.2020.113190, DOI | 2020-JAN | 7.39 | 2021-FEB | 2021-JUN | □□□□□□□□□□□□□□□□□□□□□□□□□□□□□□□□ |
| Pfefferbaum B, 2020, NEW ENGL J MED, V383, P510, DOI 10.1056/NEJMp2008017, DOI | 2020-JAN | 7.39 | 2021-FEB | 2021-JUN | □□□□□□□□□□□□□□□□□□□□□□□□□□□□□□□□ |
| Liu S, 2020, LANCET PSYCHIAT, V7, P0, DOI 10.1016/S2215-0366(20)30077-8, DOI | 2020-JAN | 7.32 | 2020-AUG | 2020-DEC | □□□□□□□□□□□□□□□□□□□□□□□□□□□□□□□□ |
| Parker A, 2015, CRIT CARE MED, V43, P1121, DOI 10.1097/CCM.0000000000000882, DOI | 2015-JAN | 7.18 | 2020-FEB | 2020-NOV | □□□□□□□□□□□□□□□□□□□□□□□□□□□□□□□□ |
| Yang Y, 2020, LANCET PSYCHIAT, V7, P0, DOI 10.1016/S2215-0366(20)30079-1, DOI | 2020-JAN | 7.12 | 2021-MAY | 2021-SEP | □□□□□□□□□□□□□□□□□□□□□□□□□□□□□□□□ |
| Cloitre M, 2018, ACTA PSYCHIAT SCAND, V138, P536, DOI 10.1111/acps.12956, DOI | 2018-JAN | 6.97 | 2021-OCT | 2022-MAY | □□□□□□□□□□□□□□□□□□□□□□□□□□□□□□□□ |
| Tang W, 2020, J AFFECT DISORDERS, V274, P1, DOI 10.1016/j.jad.2020.05.009, DOI | 2020-JAN | 6.96 | 2020-NOV | 2021-JUN | □□□□□□□□□□□□□□□□□□□□□□□□□□□□□□□□ |
| Wang C, 2020, INT J ENV RES PUB HE, V17, P0, DOI 10.3390/ijerph17072459, DOI | 2020-JAN | 6.94 | 2020-SEP | 2021-JAN | □□□□□□□□□□□□□□□□□□□□□□□□□□□□□□□□ |
| Liberzon I, 2016, NEURON, V92, P14, DOI 10.1016/j.neuron.2016.09.039, DOI | 2016-JAN | 6.85 | 2020-JAN | 2020-OCT | □□□□□□□□□□□□□□□□□□□□□□□□□□□□□□□□ |
| Xiong J, 2020, J AFFECT DISORDERS, V277, P55, DOI 10.1016/j.jad.2020.08.001, DOI | 2020-JAN | 6.76 | 2022-MAR | 2022-JUN | □□□□□□□□□□□□□□□□□□□□□□□□□□□□□□□ |
| De P, 2020, J AFFECT DISORDERS, V275, P48, DOI 10.1016/j.jad.2020.06.022, DOI | 2020-JAN | 6.69 | 2021-JUN | 2022-APR | □□□□□□□□□□□□□□□□□□□□□□□□□□□□□□□□ |
| Wang C, 2020, LANCET, V395, P470, DOI 10.1016/S0140-6736(20)30185-9, DOI | 2020-JAN | 6.68 | 2020-DEC | 2021-FEB | □□□□□□□□□□□□□□□□□□□□□□□□□□□□□□□□ |
| Tan B, 2020, ANN INTERN MED, V173, P317, DOI 10.7326/M20-1083, DOI | 2020-JAN | 6.65 | 2021-JAN | 2021-MAR | □□□□□□□□□□□□□□□□□□□□□□□□□□□□□□□□ |
| Duncan L, 2018, MOL PSYCHIATR, V23, P666, DOI 10.1038/mp.2017.77, DOI | 2018-JAN | 6.56 | 2020-JAN | 2020-APR | □□□□□□□□□□□□□□□□□□□□□□□□□□□□□□□□ |

**Supplementary Table 3. Top 10 articles with the strongest centrality divergence scores for the 2021-2022 networks.**

| Number of Current Citations in the World (June 2022) | Modularity Divergence (Modularity Change Rate) | Centrality Divergence | Authors, Year | Journal | Title | DOI |
|---|---|---|---|---|---|---|
| January 2021- June 2022 | | | | | | |
| 129 | 97.8 | 0.16 | Ettorre et al. 2021 | Int. J. Environ. Res. Public Health | Post-Traumatic Stress Symptoms in Healthcare Workers Dealing with the COVID-19 Pandemic: A Systematic Review | 10.3390/ijerph18020601 |
| 43 | 95.5 | 0.11 | Zhang et al. 2022 | J Clin Invest | Genetic evidence suggests posttraumatic stress disorder as a subtype of major depressive disorder | 10.1172/JCI145942 |
| 56 | 91.8 | 0.05 | Chatzittofis et al. 2021 | Int. J. Environ. Res. Public Health | Impact of the COVID-19 Pandemic on the Mental Health of Healthcare Workers | 10.3390/ijerph18041435 |
| 32 | 90.2 | 0.04 | Murphy et al. 2022 | BMJ Military Health | Exploring the impact of COVID-19 and restrictions to daily living as a result of social distancing within veterans with pre-existing mental health difficulties | 10.1136/bmjmilitary-2020-001622 |
| 56 | 89.6 | 0.03 | Havaei et al. 2022 | Healthcare | Nurses' Workplace Conditions Impacting Their Mental Health during COVID-19: A Cross-Sectional Survey Study | 10.3390/healthcare9010084 |
| 53 | 86.5 | 0.01 | Varghese et al. 2021 | J Glob Health | Decline in the mental health of nurses across the globe during COVID-19: A systematic review and meta-analysis | 10.7189/jogh.11.05009 |
| 49 | 82.3 | 0.01 | Imran et al. 2021 | BMJ | Psychological impact of COVID-19 pandemic on postgraduate trainees: a cross-sectional survey | 10.1136/postgradmedj-2020-138364 |
| 38 | 81.2 | 0.00 | Drane et al. 2020 | CNS Spectr | A framework for understanding the pathophysiology of functional neurological disorder | 10.1017/S1092852920001789 |
| 54 | 72 | 0.00 | Lamb et al. 2021 | Occup Environ Med | Psychosocial impact of the COVID-19 pandemic on 4378 UK healthcare workers and ancillary staff: initial baseline data from a cohort study collected during the first wave of the pandemic | 10.1136/oemed-2020-107276 |
| 31 | 65.2 | 0.00 | Highland et al. 2021 | Pharmacol Rev | Hydroxynorketamines: Pharmacology and Potential Therapeutic Applications | 10.1124/pharmrev.120.000149 |

*The modularity of a network is a measure of the overall structure of the network. Its range is between −1 and 1. The modularity change rate of a scientific paper measures the relative structural change due to the information from the published paper with reference to a baseline network.*
*The centrality divergence metric measures the structural variation caused by an article a in terms of the divergence of the distribution of betweenness centrality CB(vi) of nodes vi in the baseline network. The centrality divergence metric is potentially valuable for detecting boundary-spanning activities at interdisciplinary levels.*

**Supplementary Table 4. The top countries and institutions, ranked by centrality and citation counts (1978-2022 and 2000-2022 period).**

| 1978-2022 | | | |
|---|---|---|---|
| **Countries Ranked by Centrality[a]** | **Degree of Centrality** | **Countries Ranked by Citation Counts** | **Total Number of Citations** |
| 1. United States of America | 0.73 | 1. United States of America | 20,156 |
| 2. Australia | 0.24 | 2. United Kingdom | 4,158 |
| 3. United Kingdom | 0.17 | 3. Germany | 3,334 |
| 4. France | 0.16 | 4. Australia | 2,728 |
| 5. Canada | 0.14 | 5. Canada | 2,418 |
| 6. Italy | 0.11 | 6. Peoples Republic of China | 2,243 |
| 7. Netherlands | 0.09 | 7. Netherlands | 1,996 |
| 8. Germany | 0.09 | 8. Italy | 1,508 |
| 9. Russia | 0.09 | 9. France | 1,411 |
| 10. Spain | 0.09 | 10. Israel | 1,223 |
| **2000-2022** | | | |
| **Institutions Ranked by centraLity** | **Degree of Centrality** | **Institutions Ranked by Citation Counts** | **Total Number of Citations** |
| 1. Duke University | 0.05 | 1. Harvard University | 1611 |
| 2. Harvard University | 0.04 | 2. University of Columbia | 872 |
| 3. Emory University | 0.04 | 3. Yale University | 866 |
| 4. University of Pennsylvania | 0.04 | 4. Boston University | 849 |
| 5. Technische Universität Dresden | 0.04 | 5. Kings College London | 843 |
| 6. King's College London | 0.04 | 6. University of Washington | 778 |
| 7. Columbia University | 0.03 | 7. Emory University | 740 |
| 8. Washington University | 0.03 | 8. University of Michigan | 689 |
| 9. Yale University | 0.03 | 9. University of California San Diego | 665 |
| 10. University California Los Angeles | 0.03 | 10. Duke University | 608 |

a Betweenness centrality scores are normalized to the unit interval of [0,1]. A node of high betweenness centrality is usually one that connects two or more large groups of nodes. A node with a strong betweenness centrality score has a great influence on a network.
